# Syntheses and Antibiotic Evaluation of* 2*-{[(*2R,4R*)-*4*-Carboxy-*2*-hydroxypyrrolidin-*1*-yl]carbonyl}benzene-*1,5*-dicarboxylic Acids and* 2*-Carbamoylbenzene-*1,5*-dicarboxylic Acid Analogues

**DOI:** 10.1155/2016/9346585

**Published:** 2016-02-14

**Authors:** Abdulrazaq Tukur, Isaac Asusheyi Bello, Neil Anthony Koorbanally, James Dama Habila

**Affiliations:** ^1^Department of Chemistry, Ahmadu Bello University, Zaria 810001, Nigeria; ^2^School of Chemistry and Physics, University of KwaZulu-Natal, Private Bag X 54001, Durban 4000, South Africa

## Abstract

Our search for new antibiotics led to the syntheses and biological evaluation of new classes of dicarboxylic acid analogues. The syntheses involve nucleophilic addition of different substituted benzylamine, aniline, alkylamine, and 4-hydroxyl-L-proline with carbamoylbenzoic acid. The results of the antimicrobial activity as indicated by the zone of inhibition (ZOI) showed that** Z**_**10**_ is the most active against* Pseudomonas aeruginosa* (32 mm) and least active against* Candida stellatoidea* (27 mm) and Vancomycin Resistant* Enterococci (VRE)* (27 mm), while** Z**_**7**_ shows the least zone of inhibition (22 mm) against Methicillin Resistant* Staphylococcus aureus (MRSA)*. The minimum inhibition concentration (MIC) determination reveals that** Z**_**10**_ inhibits the growth of tested microbes at a low concentration of 6.25 *μ*g/mL, while** Z**_**9**_ and** Z**_**12**_ inhibits the growth of most microbes at a concentration of 12.5 *μ*g/mL, recording the least MIC. The Minimum Bactericidal/Fungicidal Concentration (MBC/MFC) results revealed that** Z**_**10**_ has the highest bactericidal/fungicidal effect on the test microbes, at a concentration of 12.5 *μ*g/mL, with the exception of* Candida stellatoidea *and Vancomycin Resistant* Enterococci (VRE)* with MBC/MFC of 25 *μ*g/mL. The result of this investigation reveals the potential of the target compounds (**Z**_**1**–**3**,**5**,**7**–**12**_) in the search for new antimicrobial agents.

## 1. Introduction

Development of novel bioactive drugs in chemical warfare against bacteria, fungi, and other infectious diseases has become an important and challenging task for the synthetic and medicinal chemists. Many research programs are tailored towards the design and synthesis of new drugs, for their chemotherapeutic application. The emergence of antimicrobial resistance threatens the effective prevention and treatment of an ever increasing range of infections caused by bacteria, parasites, virus, and fungi. New resistance mechanisms are emerging and spreading globally; the appearance and widespread use of fake and substandard drugs have further compounded the problem [1]. The HIV epidemic around the world has led to an increase in the number of immunocompromised patients, which in turn has led to an increase in the number of systemic bacterial and fungal infections [2]. Compounds containing carboxylic acid functional groups are playing a major role in the field of medicine. Generally, they play an active and critical role in the biochemistry of human or animal physiology. They have been involved in studies as antibacterial [3], anti-inflammatory [4], antiplatelet [5], antimicrobial [6], anticancer [7], antifungal [8], and analgesic and antiseptic [9].

Currently, there are more than 450 clinically approved drugs containing a free carboxylic acid group [10]. To the best of our knowledge no biological studies and syntheses of these compounds have been reported. We present here the syntheses of different substituted carboxylic and dicarboxylic acid analogues, with electron withdrawing and donating groups (OH, OCH_3_, CH_3_, NO_2_, and F) and explored their potentials as antibacterial and antifungal drugs.

## 2. Materials and Methods

### 2.1. General Experimental Details

All chemicals and solvents were purchased from Sigma-Aldrich (Germany) and used as purchased without further purification. Thin-layer chromatography was performed using precoated silica gel 60 (F_254_) from MERCK (Germany). Spots on the TLC plates were visualized under UV light (254 nm and 366 nm) and by heating with 10% sulphuric acid in MeOH. The melting point was recorded using a Gallenkamp melting point apparatus. The UV-VIS analysis was carried out on a Perkin Elmer Lambda 35 UV/VIS Spectrometer, scanning from 200 to 400 nm. Absorbance was measured with a 1 cm quartz cell. The infrared spectra of the solid products were recorded on a Perkin Elmer Spectrum 100 FTIR spectrometer using ATR sampling accessory. ^1^H and ^13^C-NMR spectra were recorded using Bruker Avance^III^ 400 MHz Spectrometer at room temperature (400 MHz for ^1^H and 100 MHz for ^13^C), using TMS as reference. Chemical shift values (*δ*) were reported in parts per million (ppm) relative to TMS and coupling constants are given in Hz. The solvent used for these measurements was deuterated DMSO. Multiplicities are given as follows: singlet (s), doublet (d), doublet of doublets (dd), triplet (t), and multiplet (m).

### 2.2. General Procedure for the Synthesis of** Z**_**1–3,5,7–12**_

**Scheme 1:**
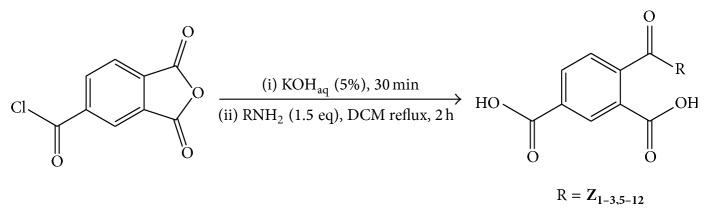
Preparation of carbamoylbenzoic and carbamoyldibenzoic acid.

A solution of 1,3-dioxo-2-benzofuran-5-carbonyl chloride (5 g, 23.8 mmol) was weighed into a round bottom flask containing 100 mL of KOH_aq_ (5%) and then stirred for 30 min; the solid product (1,3-dioxo-2-benzofuran-5-carboxylic acid) formed was then filtered under suction. The products were pure enough for further reaction without further purification; this acid (0.3 g, 1.56 mmol) was further transferred to a 10 mL round bottom flask containing dichloromethane (7 mL) and a magnetic stirrer and 1.5 equiv. of different classes of amine were each added individually to the respective flasks and the mixture refluxed for at least two hours. The heat was then removed and the reaction stirred for a further 30 min. The reaction mixtures were allowed to stand at room temperature for a further 30 min; the solid precipitates formed were then filtered under suction and washed thoroughly with dichloromethane to remove any excess amines (Scheme 1). After drying, they were recrystallized from dichloromethane to give the purified products (Table 1).

*4-[(4-Fluorobenzyl)carbamoyl]benzene-1,5-dicarboxylic Acid (* ***Z***_***1***_). Bone white solid powder (99% yield) was prepared according to the general procedure from 1,3-dioxo-2-benzofuran-5-carboxylic acid** 2** (0.3 g, 1.56 mmol), 4-fluorobenzylamine (0.26 g, 2.1 mmol), and DCM (7 mL) as solvent and purified by recrystallization with DCM. Melting point 173–175°C, UV analysis *λ*_max_(log⁡*ε*), 245 (4.39), 300 (3.92). IR (cm^−1^); 2872 (COOH), 1603 and 1536 (CONH), 896 (=CH) and 828–808 (C=C). ^1^H-NMR (400 MHz, DMSO-d6) *δ*_H_ 8.72 (d, 1H,* J* = 1.32 Hz, H-3), 8.18 (d, 1H,* J* = 8.08 Hz, H-6), 7.96 (d, 1H,* J* = 8.12 Hz, H-4), 7.54 (q, 2H,* J* = 2.60, 5.68 Hz, H-2′, 6′), 7.26 (d, 2H,* J* = 8.84 Hz, H-3′, 5′), 4.03 (s, 2H, H-10). ^13^C-NMR (100 MHz, DMSO-d6); 168.33 (C-7), 167.88 (C-9), 167.81 (C-8), 161.94 (d, *J*_CF_ = 242.73 Hz, C-4′), 137.21 (C-2), 136.82 (C-1), 134.43 (C-5), 133.57 (C-6), 132.39 (C-4), 131.25 (d, *J*_CF_ = 3.08 Hz, C-1′), 131.02 (d, *J*_CF_ = 8.28 Hz, C-2′, 6′), 130.46 (C-3), 115.32 (d, *J*_CF_ = 21.46 Hz, C-3′, 5′), 41.64 (C-10). GC-MS (*m/z*, rel. int.) 317.1 [M]^+^ (74), 299.1 (100), 244.1 (24), 122.1 (23), LRMS 313.3 [M − Na]^+^ for C_16_H_12_FNO_5_, calculated mass 317.3 (see Appendixes  1–7 available online as Supplementary Material at http://dx.doi.org/10.1155/2016/9346585).

*4-[(3-Fluorobenzyl)carbamoyl]benzene-1,5-dicarboxylic Acid (* ***Z***_***2***_). Bone white solid powder (55% yield) was prepared according to the general procedure from 1,3-dioxo-2-benzofuran-5-carboxylic acid** 2** (0.3 g, 1.56 mmol), 3-fluorobenzylamine (0.26 g, 2.1 mmol), and DCM (7 mL) as solvent and purified by recrystallization with DCM. Melting point 200-201°C, UV analysis (*λ*_max_(log⁡*ε*)_ethanol_), 250 (4.26), 300 (3.48). IR (cm^−1^); 2868 (COOH), 1271 and 1246 (O-C), 1691 and 1501 (CONH), 1691 and 1574 (C=C) and 913 (=CH). ^1^H-NMR (400 MHz, DMSO-d6) *δ*_H_ 8.72 (s, 1H, H-6), 8.18 (d, 1H,* J* = 8.04 Hz, H-3), 7.96 (dd, 1H,* J* = 1.80, 8.04 Hz, H-4), 7.46–7.40 (m, 1H, H-5′), 7.33–7.27 (m, 3H, H-2′, H-6′, N-H), 7.33–7.27 (m, 3H, H-2′, H-6′, N-H), 7.18–7.14 (m, 1H,* J* = 2.46, 8.66 Hz, H-4′), 4.00 (s, 2H, H-10). ^13^C-NMR (100 MHz, DMSO-d6); 168.21 (C-7), 167.74 (C-9), 167.69 (C-8), 162.04 (d, *J*_CF_ = 241.96 Hz, C-3′), 139.36 (d, *J*_CF_ = 8.53 Hz, C-1′), 136.99 (C-1), 134.51 (C-2), 133.58 (C-3), 133.58 (C-5), 132.42 (C-6), 130.43 (d, *J*_CF_ = 8.36 Hz, C-5′), 130.42 (C-4), 124.44 (d, *J*_CF_ = 2.71 Hz, C-6′), 115.12 (d, *J*_CF_ = 21.67 Hz, C-2′), 114.59 (d, *J*_CF_ = 20.73 Hz, C-4′), 42.35 (C-10). GC-MS (*m/z*, rel. int.) 317.1 [M]^+^ (72), 299.1 (100), 226.1 (24), 122.1 (21), LRMS 317.1 [M − Na]^+^ for C_16_H_12_FNO_5_ calculated 317.3 (Appendixes  8–15).

*2-[(2-Fluorobenzyl)carbamoyl]benzene-1,5-dicarboxylic Acid (* ***Z***_***3***_). Bone white solid powder (35% yield) was prepared according to the general procedure from 1,3-dioxo-2-benzofuran-5-carboxylic acid** 2** (0.3 g, 1.56 mmol), 2-fluorobenzylamine (0.26 g, 2.1 mmol), and DCM (7 mL) as solvent and purified by recrystallization with DCM. Melting point 198–200°C, UV analysis *λ*_max_(log⁡*ε*), 245 (4.24), 300 (3.43). IR (cm^−1^); 2839 (COOH), 1272 and 1248 (O-C), 1684 and 1574 (CONH), 1613 and 1500 (C=C) and 907 (=CH).t6 ^1^H-NMR (400 MHz, DMSO-d6) *δ*_H_ 8.71 (s, 1H, H-6), 8.15 (d, 1H,* J* = 8.07 Hz, H-3), 7.94 (dd, 1H,* J* = 1.76, 8.07 Hz, H-4), 7.56–7.53 (m, 1H, Ar-H; H-4′, N-H), 7.39–7.34 (m, 1H, Ar-H; H-6′), 7.24–7.19 (m, 2H, Ar-H; H-3′, H-5′), 3.98 (s, 1H, H-10). ^13^C-NMR (100 MHz, DMSO-d6); 168.85 (C-7), 168.03 (C-9), 167.95 (C-8), 160.15 (d, *J*_CF_ = 243.57 Hz, C-2′), 138.53 (C-2), 136.36 (C-1), 134.31 (C-5), 133.53 (C-6), 132.22 (C-3), 130.42 (C-4), 130.42 (d, *J*_CF_ = 3.99 Hz, C-4′), 129.76 (d, *J*_CF_ = 8.13 Hz, C-6′), 124.78 (d, *J*_CF_ = 14.61 Hz, C-1′), 124.44 (d, *J*_CF_ = 3.44 Hz, C-5′), 115.15 (d, *J*_CF_ = 21.14 Hz, C-3′), 36.77 (d, *J*_CF_ = 4.24 Hz, C-10). GC-MS (*m/z*, rel. int.) 317.1 [M]^+^ (91), 299.1 (100), 244.1 (32), 122.1 (24), LRMS 313.1 [M − Na]^+^ for C_16_H_12_FNO_5_, calculated mass 317.3 (Appendixes  16–23).

*2-*{*[(2R,4R)-4-Carboxy-2-hydroxypyrrolidin-1-yl]carbonyl*}*benzene-1,5-dicarboxylic Acid (* ***Z***_***5***_). White solid powder (78% yield) was prepared according to the general procedure from 1,3-dioxo-2-benzofuran-5-carboxylic acid** 2** (0.3 g, 1.56 mmol), 4-hydroxy-L-proline (0.28 g, 2.1 mmol), and DCM (7 mL) as solvent and purified by recrystallization with DCM. Melting point 187–180°C, UV analysis *λ*_max_(log⁡*ε*), 245 (4.02), 300 (3.18). IR (cm^−1^); 2855 (COOH), 1285 and 1250 (O-C), 1689 and 1574 (CONH), 1639 and 1502 (C=C) and 916 (=CH). ^1^H-NMR (400 MHz, DMSO-d6) *δ*_H_ 8.43 (s, 1H, H-6), 8.05 (dd, 1H,* J* = 1.4, 8.04 Hz, H-4), 7.96 (dd, 1H,* J* = 2.92, 7.72 Hz, H-3), 4.41 (t, H,* J* = 4.32 Hz, H-2′), 4.31 (t, 1H,* J* = 8.52 Hz, H-4′), 3.32 [(dd, 1H,* J* = 4.04, 12.08 Hz, H^c^-5′); 3.06 (d, 1H,* J* = 12.08 Hz, H^d^-5′)], 2.21 [(dd, 1H,* J* = 7.72, 13.40 Hz, H^a^-3′); 2.06 (m, 1H,* J* = 4.32, 10.8 Hz, H^b^-3′)]. ^13^C-NMR (100 MHz, DMSO-d6); 170.41 (C-7), 167.92 (C-10), 167.34 (C-9), 166.21 (C-8), 137.87 (C-2), 133.22 (C-1), 132.29 (C-5), 131.25 (C-4), 131.03 (C-6), 130.28 (C-3), 68.75 (C-2′), 57.98 (C-4′), 53.23 (C-5′), 37.31 (C-3′). GC-MS (*m/z*, rel. int.) 323.1 [M − 4]^+^ (100), 226.1 (29), 282.1 (18), 122.1 (13), LRMS 324.3 [M − Na]^+^ for C_14_H_13_NO_8_, calculated mass 324.3 (Appendixes  24–31). 

*2-[(2,4-Difluorobenzyl)carbamoyl]benzene-1,5-dicarboxylic Acid (* ***Z***_***7***_). Pale yellow solid powder (90% yield) was prepared according to the general procedure from 1,3-dioxo-2-benzofuran-5-carboxylic acid** 2** (0.3 g, 1.56 mmol), 2,4-difluorobenzylamine (0.3 g, 2.1 mmol) and toluene (7 mL) as solvent and purified by recrystallization with DCM. Melting point 152–154°C, UV analysis *λ*_max_(log⁡*ε*), 245 (3.86), 300 (2.95). IR (cm^−1^); 2878, 2631 (COOH), 1273 and 1297 (O-C_acid_), 1687 and 1561 (CONH), 1603 and 1507 (C=C) and 847 (=CH). ^1^H-NMR (400 MHz, DMSO-d6) *δ*_H_ 8.74 (d, H,* J* = 1.80 Hz, H-6), 8.24 (d, H,* J* = 8.12 Hz, H-4), 8.01 (dd, H,* J* = 1.88, 8.08 Hz, H-3), 7.64 (q, H,* J* = 8.52, 15.16 Hz, H-6′), 7.35 (td, H,* J* = 2.48, 1.96, 2.52, 10.0 Hz, H-5′), 7.20 (td, H,* J* = 2.32, 2.12, 2.48, 8.36 Hz, H-3′), 4.07 (s, 2H, H-10). ^13^C-NMR (100 MHz, DMSO-d6); 167.46 (C-7), 167.46 (C-9), 167.16 (C-8), 161.34 (q, *J*_CF_ = 12.22, 246.21 Hz, C-4′), 160.55 (q, *J*_CF_ = 12.43, 249.81 Hz, C-2′), 138.05 (C-2), 134.73 (C-5), 133.70 (C-1), 133.63 (C-6), 132.77 (C-4), 132.69 (q, *J*_CF_ = 5.65, 10.06 Hz, C-6′), 117.89 (d, *J*_CF_ = 3.49 Hz, C-1′), 111.77 (q, *J*_CF_ = 3.65, 17.61 Hz, C-5′), 104.04 (t, *J*_CF_ = 25.63 Hz, C-3′), 35.41 (d, *J*_CF_ = 3.58 Hz, C-10). GC-MS (*m/z*, rel. int.) 331.1 [M + 4]^+^ (100), 244.1 (32), 300.1 (16), 103.1 (10), LRMS 333.1 [M − Na]^+^ for C_16_H_11_F_2_NO_5_, calculated mass 335.3 (Appendixes  32–39).

*2-(Butylcarbamoyl)benzene-1,5-dicarboxylic Acid (* ***Z***_***8***_). Bone white solid powder (54% yield) was prepared according to the general procedure from 1,3-dioxo-2-benzofuran-5-carboxylic acid** 2** (0.3 g, 1.56 mmol), butylamine (0.15 g, 2.1 mmol), and DCM (7 mL) as solvent and purified by recrystallization with DCM. Melting point 118–120°C, UV analysis *λ*_max_(log⁡*ε*), 245 (4.08), 300 (3.26). IR (cm^−1^); 3061, 2959 (COOH), 1239 and 1267 (O-C_acid_), 1654 and 1562 (CONH), 1622 and 1516 (C=C) and 780 (=CH). ^1^H-NMR (400 MHz, DMSO-d6) *δ*_H_ 8.71 (s, 1H, H-6), 8.19 (d, 1H,* J* = 7.96 Hz, H-3), 7.97 (d, 1H,* J* = 7.96 Hz, H-4), 2.81 (t, 2H,* J* = 7.16 Hz, H-1′), 1.53 (m, 2H,* J* = 6.92, 7.16 Hz, H-2′), 1.34 (q, 2H,* J* = 7.16 Hz, H-3′), 0.88 (t, 3H,* J* = 7.16 Hz, H-4′). ^13^C-NMR (100 MHz, DMSO-d6); 167.99 (C-7), 167.84 (C-9), 167.79 (C-8), 137.03 (C-2), 136.52 (C-1), 134.45 (C-5), 133.55 (C-6), 132.47 (C-3), 130.48 (C-4), 38.44 (C-1′), 29.12 (C-2′), 19.07 (C-3′), 13.43 (C-4′). GC-MS (*m/z*, rel. int.) 261.1 [M + 4]^+^ (41), 218.1 (100), 185.1 (74), 261.1 (41), LRMS 261.1 [M − Na]^+^ for C_13_H_15_NO_5_, calculated mass 265.3 (Appendixes  40–46). 

*2-[(3-Methoxypropyl)carbamoyl]benzene-1,5-dicarboxylic Acid (* ***Z***_***9***_). White solid powder (24% yield) was prepared according to the general procedure from 1,3-dioxo-2-benzofuran-5-carboxylic acid** 2** (0.3 g, 1.56 mmol), 3-methoxypropylamine (0.19 g, 2.1 mmol), and DCM (7 mL) as solvent and purified by recrystallization with DCM. Melting point 135-136°C, UV analysis *λ*_max_(log⁡*ε*), 245 (3.68), 300 (2.48). IR (cm^−1^); 3136, 2932 (COOH), 1356 and 1297 (O-C_acid_), 1622 and 1529 (CONH), 1477 (C=C) and 752 (=CH). ^1^H-NMR (400 MHz, DMSO-d6) *δ*_H_ 8.67 (d, H,* J* = 1.24 Hz), 8.12 (d, H,* J* = 8.00 Hz, H-4), 7.91 (dd, H,* J* = 1.20, 8.00 Hz, H-3), 3.39 (t, 2H,* J* = 6.00 Hz, H-3′), 3.22 (s, H, H-4′), 2.86 (t, 2H,* J* = 7.28 Hz, H-1′), 1.82 (qt, 2H,* J* = 6.48, 7.00, 13.48 Hz, H-2′). ^13^C-NMR (100 MHz, DMSO-d6); 169.04 (C-7), 168.19 (C-9), 168.08 (C-8), 139.91 (C-2), 135.83 (C-1), 134.15 (C-5), 133.45 (C-6), 132.05 (C-4), 130.34 (C-3), 68.92 (C-3′), 57.87 (C-4′), 36.52 (C-1′), 27.44 (C-2′). GC-MS (*m/z*, rel. int.) 281.1 [M]^+^ (28), 207.1 (100), 32.1 (93), 45 (77), LRMS 277.1 [M − Na]^+^ for C_13_H_15_NO_6_, calculated mass 281.3 (Appendixes  47–54). 

*2-(Benzylcarbamoyl)benzene-1,5-dicarboxylic Acid (* ***Z***_***10***_). Cream coarse powder (65% yield) was prepared according to the general procedure from 1,3-dioxo-2-benzofuran-5-carboxylic acid** 2** (0.3 g, 1.56 mmol), benzylamine (0.23 g, 2.1 mmol), and DCM (7 mL) as solvent and purified by recrystallization with DCM. Melting point 163–165°C, UV analysis *λ*_max_(log⁡*ε*), 245 (3.78), 300 (2.78). IR (cm^−1^); 3396, 3034 (COOH), 1361 and 1288 (O-C_acid_), 1622 and 1551 (CONH), 1499 (C=C) and 693 (=CH). ^1^H-NMR (400 MHz, DMSO-d6) *δ*_H_ 8.72 (d, 1H,* J* = 1.60 Hz, H-6), 8.15 (d, 1H,* J* = 8.04 Hz, H-3), 7.94 (dd, 1H,* J* = 1.60, 8.04 Hz, H-4), 7.46–7.31 (d, 5H, Ar-H, H-2′,6′3′5′4′), 4.00 (s, 2H, H-10). ^13^C-NMR (100 MHz, DMSO-d6); 169.03 (C-7), 168.15 (C-9), 168.04 (C-8), 139.38 (C-1′), 136.05 (C-2, C-5), 134.23 (C-1), 133.52 (C-6), 132.14 (C-3), 130.40 (C-4), 128.46 (C-2′, C-6′), 128.45 (C-3′, C-5′), 127.91 (C-4′). GC-MS (*m/z*, rel. int.) 295.1 [M + 4]^+^ (100), 278 (34), 208.1 (25), 104.1 (24), LRMS 295.1 [M − Na]^+^ for C_16_H_13_NO_5_, calculated mass 299.3 (Appendixes  55–62). 

*2-[(4,6-Dimethylpyridin-2-yl)carbamoyl]benzene-1,5-dicarboxylic Acid (* ***Z***_***11***_). Bone white powder (79% yield) was prepared according to the general procedure from 1,3-dioxo-2-benzofuran-5-carboxylic acid** 2** (0.3 g, 1.56 mmol), 2-amino-4,6-dimethylpyridine (0.26 g, 2.1 mmol), and DCM (7 mL) as solvent and purified by recrystallization with DCM. Melting point 189-190°C, UV analysis *λ*_max_(log⁡*ε*), 245 (4.31), 300 (3.88). IR (cm^−1^); 3331, 3097 (COOH), 1343 and 1296 (O-C_acid_), 1675 and 1534 (CONH), 1604 and 1477 (C=C) and 752 (=CH). ^1^H-NMR (400 MHz, DMSO-d6) *δ*_H_ 8.58 (d, 1H,* J* = 1.48 Hz, H-6), 8.09 (d, 1H,* J* = 8.04 Hz, H-4), 8.04 (dd, 1H,* J* = 1.68, 8.04 Hz, H-3), 6.47 (s, 2H, H-3′H-5′), 2.33 (s, 3H, 4′, 6′-CH_3_). ^13^C-NMR (100 MHz, DMSO-d6); 167.93 (C-7), 167.63 (C-9), 166.79 (C-8), 154.86 (C-2′), 154.81 (C-6′), 147.41 (C-4′), 138.32 (C-2), 134.24 (C-1), 132.57 (C-5), 132.28 (C-6), 131.49 (C-4), 130.80 (C-3), 113.24 (C-5′), 108.20 (C-3′), 21.12 (6′-CH_3_), 19.14 (4′-CH_3_). GC-MS (*m/z*, rel. int.) 314.2 [M]^+^ (5), 32.1 (100), 43 (65), 207.1 (20), 77.1 (18), LRMS 304.1 [M + Na]^+^ for C_16_H_14_N_2_O_5_, calculated mass 314.3 (Appendixes  63–70). 

*2-[(2-Nitrophenyl)carbamoyl]benzene-1,5-dicarboxylic Acid (* ***Z***_***12***_). Pale brown powder (36% yield) was prepared according to the general procedure from 1,3-dioxo-2-benzofuran-5-carboxylic acid** 2** (0.3 g, 1.56 mmol), 2-nitrobenzylamine (0.29 g, 2.1 mmol), and DCM (7 mL) as solvent and purified by recrystallization with DCM. Melting point 204-205°C, UV analysis *λ*_max_(log⁡*ε*), 245 (4.26), 300 (3.48). IR (cm^−1^); 3317, 3062 (COOH), 1290 and 1249 (O-C_acid_), 1679 and 1574 (CONH), 1500 (C=C) and 752 (=CH). ^1^H-NMR (400 MHz, DMSO-d6) *δ*_H_ 8.21 (d, 3H,* J* = 1.48 Hz, H-4,5′,6), 8.12 (dd, 2H,* J* = 1.56, 7.96 Hz, H-3, H-3′), 7.75 (d, 2H,* J* = 7.96 Hz, H-4′, 6′). ^13^C-NMR (100 MHz, DMSO-d6); 168.41 (C-7), 167.47 (C-9), 165.95 (C-8), 137.34 (C-2), 132.42 (C-1), 131.76 (C-4, 5′,6), 129.29 (C-3, C-3′), 128.58 (C-4′, 6′). GC-MS (*m/z*, rel. int.) 331.1 [M − H]^+^ (94), 32.1 (100), 44 (71), 207.1 (67), 244.1 (39), LRMS 333.2 [M − 3H]^+^ for C_15_H_10_N_2_O_7_, calculated mass 330.3 (Appendixes  71–78).

## 3. Biological Assay

### 3.1. Clinical Isolate

The test compounds (**Z**_**1**–**3**,**5**,**7**–**12**_) were evaluated on the following isolates, obtained from the Department of Medical Microbiology, Ahmadu Bello University Teaching Hospital Zaria, Nigeria (ABUTH):* Candida stellatoidea, Candida tropicalis, Candida krusei, Candida albicans, Shigella dysenteriae, Salmonella typhi, Klebsiella pneumonia, Pseudomonas aeruginosa, Proteus vulgaris, Escherichia coli, Corynebacterium ulcerans, Streptococcus pyogenes, Staphylococcus aureus, *Vancomycin Resistant* Enterococci (VRE), *and Methicillin Resistant* Staphylococcus aureus (MRSA)*.

### 3.2. Antimicrobial Susceptibility Test

The cork and bore diffusion method as reported by Karou et al. [11] was used to determine the antimicrobial activity of the test compounds. Pure cultures of the organism were inoculated on to Mueller Hinton Agar (MERCK) and incubated for 24 h at 38°C for bacteria and 48 h at 34°C for fungi. About 5 discrete colonies were aseptically transferred using sterile wire loops into tubes containing sterile normal saline (0.85% NaCl) and were adjusted to a turbidity of 0.5 McFarland Standard. The suspensions were then inoculated on the surface of sterile Mueller-Hinton Agar plates using sterile cotton swabs. A sterile 6 mm diameter Cork borer was used to make holes (wells) into the set of inoculated Mueller-Hinton Agar. The wells were filled with different concentration of the test compounds. The plates were then incubated; all the tests were performed in triplicate and the antimicrobial activities were determined as mean diameter of inhibition zone (mm) produced by the test compounds.

### 3.3. Minimum Inhibition Concentration (MIC)

The MIC was determined for the compounds using microbroth dilution method in accordance with National Committee for Clinical Laboratory Standard [12]. Serial dilution of the least concentration of the compounds that showed activity was prepared using test tubes containing 9 mL of double strength nutrient broth (OXOID). The test tubes were inoculated with the suspension of the standardized inoculum and incubated at 38°C for 24 h. MICs were recorded as the lowest concentration of the compounds showing no visible growth (turbidity) in the broth.

### 3.4. Minimum Bactericidal and Minimum Fungicidal Concentration (MBC/MFC)

The MBC/MFC was determined by aseptically inoculating aliquots of culture, from the minimum inhibition concentration (MIC) tubes that showed no growth, on sterile nutrient Agar (OXOID) plates incubated at 38°C for bacteria and 34°C for fungi for 48 h. The MBC/MFC was recorded as the lowest concentration of compounds showing no bacterial/fungal growth at all.

## 4. Results and Discussion

### 4.1. Chemistry

Our synthetic approach involved two steps (Scheme 1). In the first step, the acid chloride is hydrolysed by the aqueous KOH to form the corresponding carboxylic acid. The reaction takes place at room temperature under stirring condition for 30 min. The second step involves nucleophilic addition of the substituted amine, which attack the anhydride ring, breaking it open to form the second carboxylic acid group and an amide. This reaction was carried out in DCM under reflux condition for at least 2 h. The overall yields are given in the experimental section and they ranges between 35 and 99%.

The structures of the compounds were confirmed by the use of ^1^H and ^13^C NMR with application of 2D NMR where necessary.

### 4.2. Biological Results

The synthesised compounds were tested against eleven bacteria including two resistance bacteria, Methicillin Resistant* Staphylococcus aureus (MRSA), *Vancomycin Resistant* Enterococci (VRE),* and four fungi. The test compounds had significant zones of inhibitions against all tested organism as compared to the standard drug. Compound** Z**_**10**_ had the best activity among the compounds tested, with zone of inhibition ranging from 32 to 27 mm on the test microbes, but was not able to inhibit five bacteria (*S. dysenteriae, P. aeruginosa, P. vulgaris, E. coli, *and* S. pyogenes*) (Table 2). This was the compound without any substitution on the benzyl ring. Compounds** Z**_**7**_ and** Z**_**11**_ had zones ranging from 30 to 22 mm, while** Z**_**1**_,** Z**_**2**_, and** Z**_**5**_ were in the range between 29 and 24 mm. The zone of inhibition of** Z**_**3**_,** Z**_**9**_, and** Z**_**12**_ was in the range between 28 and 20 mm, as compared to the zone of the standard drugs (32 to 40 mm).

Minimum inhibitory concentration (MIC) results (Table 3) reveal that a low concentration of 6.25 *μ*g/mL of the test compounds (**Z**_**1**–**3**_,** Z**_**5**,**7**–**12**_) inhibited the growth of the resistance bacteria (*MRSA*); the only exception was** Z**_**1**_ which inhibited at 12.5 *μ*g/mL. Three of the compounds (**Z**_**2**,**5**,**10**_) inhibited the resistance* VRE* at a concentration of 6.25 *μ*g/mL while** Z**_**8**_,** Z**_**9**_, and** Z**_**12**_ inhibited* VRE* at a higher concentration of 12.5 *μ*g/mL. Generally, other test microbes have shown MIC ranging between 6.25 and 50 *μ*g/mL. These test compounds were also found to be both bactericidal and fungicidal at a concentration of 12.5 *μ*g/mL, as recorded by the minimum bactericidal/fungicidal concentration (MBC/MFC) analysis (Table 4).

## 5. Conclusion

The different substituted test compounds were successfully synthesised and their structures were confirmed by NMR analysis. Generally,* 2-(benzylcarbamoyl)benzene-1,5-dicarboxylic acid* (**Z**_**10**_) showed a better activity against resistance bacteria* MRSA* and* VRE* and other microbes. Although the test compounds were not as active as the standard drugs, sparfloxacin and fluconazole, the compounds may be employed in situations where there is resistance to antimicrobial drugs. Compound** Z**_**10**_ is therefore a lead candidate in the search for an antimicrobial agent.

## Supplementary Material

The supplementary materials Appendix 1-78 shows the spectroscopy results for the synthesized compounds. Compound Z_1_ for example has its spectral data in Appendix 1-7, where Appendix 1 is the UV Spectrum, Appendix 2 is the 1H NMR spectrum, Appendix 3 is the ^13^C NMR spectrum, Appendix 4 is the DEPT-90, DEPT-135 and decouple ^13^C NMR Spectra combined. Appendix 5 shows the COSY spectrum, Appendix 6 is the HSQC spectrum and Appendix 7 is the NOESY spectrum. A similar arrangement is obtainable for the other compounds.

## Figures and Tables

**Table 1:** The synthesised compounds and their percentage yield (%).

Sample	R	Product	Yield (%)
**Z**_**1**_	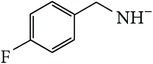	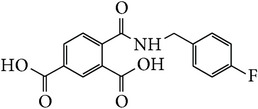	99

**Z**_**2**_	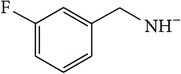	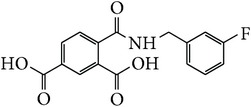	55

**Z**_**3**_	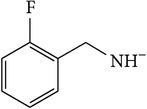	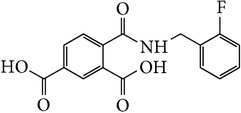	35

**Z**_**5**_	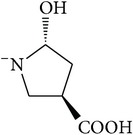	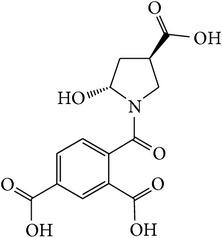	78

**Z**_**7**_	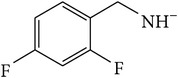	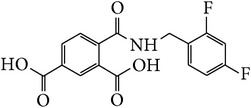	90

**Z**_**8**_	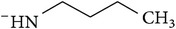	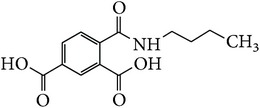	54

**Z**_**9**_	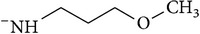	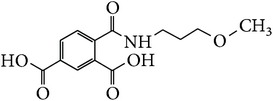	24

**Z**_10_	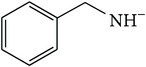	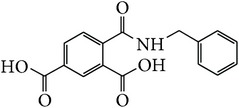	65

**Z**_11_	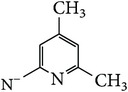	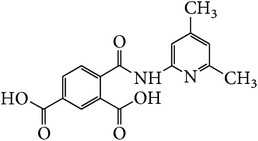	79

**Z**_12_	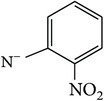	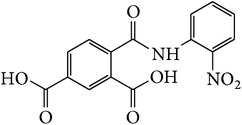	36

**Table 2:** Zone of inhibition (mm).

Test organism	**Z**_**1**_	**Z**_**2**_	**Z**_**3**_	**Z**_**5**_	**Z**_**7**_	**Z**_**8**_	**Z**_**9**_	**Z**_**10**_	**Z**_**11**_	**Z**_**12**_	Sp	Fl
*MRSA*	26	28	—	—	22	28	—	29	28	—	35	—
*VRE*	—	24	—	29	—	24	20	27	—	24	—	—
*S*. *aureus*	27	29	24	—	24	27	—	29	29	26	37	—
*S*. *pyogenes*	25	—	27	27	—	—	24	—	—	24	34	—
*C*. *ulcerans*	—	24	23	—	26	24	28	30	—	—	32	—
*E*. *coli*	29	27	26	29	26	—	—	—	27	25	37	—
*P*. *vulgaris*	—	—	26	28	—	—	25	—	25	27	—	—
*P*. *aeruginosa*	—	24	—	24	30	31	28	32	—	24	35	—
*K*. *pneumonia*	27	29	28	—	—	—	25	29	30	27	39	—
*S*. *typhi*	—	27	26	—	29	26	26	30	—	24	42	—
*S*. *dysenteriae*	29	—	—	29	25	—	27	—	24	—	40	—
*C*. *albicans*	24	—	27	24	—	28	24	29	29	27	—	35
*C*. *krusei*	27	28	—	26	25	24	22	28	24	27	—	35
*C*. *tropicalis*	—	26	—	24	—	26	26	29	23	24	—	30
*C*. *stellatoidea*	26	27	28	29	27	28	24	27	27	23	—	34

—: not determined.

**Table 3:** Minimum inhibitory concentration (MIC) (*μ*g/mL).

Test organism	**Z**_**1**_	**Z**_**2**_	**Z**_**3**_	**Z**_**5**_	**Z**_**7**_	**Z**_**8**_	**Z**_**9**_	**Z**_**10**_	**Z**_**11**_	**Z**_**12**_
*MRS*	12.50	6.2	—	—	6.2	6.2	—	6.2	6.2	—
*VRE*	—	6.2	—	6.2	—	12.5	12.5	6.2	—	12.5
*S. aureus*	6.2	12.5	12.5	—	6.2	6.2	—	6.2	6.2	12.5
*S. pyogenes*	12.5	6.2	6.2	6.2	—	—	12.5	—	—	12.5
*C. ulcerans*	—	12.5	12.5	—	12.5	—	6.2	6.2	—	—
*E. coli*	6.2	6.2	12.5	6.2	12.5	12.5	6.2	—	6.2	12.5
*P. vulgaris*	—	—	12.5	6.2	12.5	—	—	—	12.5	6.2
*P. aeruginosa*	—	12.5	—	12.5	—	—	12.5	6.2	—	12.5
*K. pneumonia*	6.2	6.2	6.2	—	6.2	6.2	6.2	6.2	6.2	6.2
*S. typhi*	—	—	12.5	—	—	—	12.5	6.2	—	12.5
*S. dysenteriae*	6.2	6.2	—	6.2	6.2	12.5	12.5	—	12.5	—
*C. albicans*	12.5	—	6.2	12.5	12.5	6.2	12.5	6.2	6.2	6.2
*C. krusei*	6.2	6.2	—	12.5	—	12.5	12.5	6.2	12.5	6.2
*C. tropicalis*	—	12.5	—	12.5	6.2	12.5	12.5	6.2	12.5	12.5
*C. stellatoidea*	12.5	6.2	6.2	6.2	—	6.2	12.5	6.2	6.2	12.5

—: no MIC.

**Table 4:** Minimum bactericidal/fungicidal concentration (MBC/MFC) (*μ*g/mL).

Test organism	**Z**_**1**_	**Z**_**2**_	**Z**_**3**_	**Z**_**5**_	**Z**_**7**_	**Z**_**8**_	**Z**_**9**_	**Z**_**10**_	**Z**_**11**_	**Z**_**12**_
*MRS*	25	12.5	—	—	50	12.5	—	12.5	12.5	—
*VRE*	—	50	—	12.5	—	50	50	25	—	50
*S. aureus*	25	12.5	50	—	50	25	—	12.5	12.5	25
*S. pyogenes*	25	—	25	25	—	—	25	—	—	25
*C. ulcerans*	—	25	50	—	25	—	25	—	—	—
*E. coli*	12.5	25	25	12.5	25	50	12.5	12.5	25	25
*P. vulgaris*	—	—	25	12.5	25	—	—	—	25	25
*P. aeruginosa*	—	25	—	50	—	—	25	—	—	25
*K. pneumonia*	25	12.5	12.5	—	12.5	12.5	12.5	12.5	12.5	25
*S. typhi*	—	—	25	—	—	—	25	12.5	—	50
*S. dysenteriae*	12.5	25	—	12.5	12.5	25	25	12.5	25	—
*C. albicans*	50	—	25	25	—	12.5	50	12.5	12.5	25
*C. krusei*	25	12.5	—	25	25	25	50	12.5	50	25
*C. tropicalis*	—	25	—	50	—	25	25	12.5	50	50
*C. stellatoidea*	25	25	12.5	12.5	25	12.5	50	25	25	50

—: no MBC/MFC.
