# Supplementary material for: Syntheses and Antibiotic Evaluation of 2-{[(2R,4R)-4-Carboxy-2-hydroxypyrrolidin-1-yl]carbonyl}benzene-1,5-dicarboxylic Acids and 2-Carbamoylbenzene-1,5-dicarboxylic Acid Analogues
Source: Int J Med Chem. 2016 Feb 14;2016:9346585. doi: 10.1155/2016/9346585 (PMC4769762; doi:10.1155/2016/9346585)
Supplement: Supplementary file 1 — The supplementary materials Appendix 1-78 shows the spectroscopy results for the synthesized compounds. Compound Z1 for example has its spectral data in Appendix 1-7, where Appendix 1 is the UV Spectrum, Appendix 2 is the 1H NMR spectrum, Appendix 3 is the 13C NMR spectrum, Appendix 4 is the DEPT-90, DEPT-135 and decouple 13C NMR Spectra combined. Appendix 5 shows the COSY spectrum, Appendix 6 is the HSQC spectrum and Appendix 7 is the NOESY spectrum. A similar arrangement is obtainable for the other compounds. [file 9346585.f1.pdf]

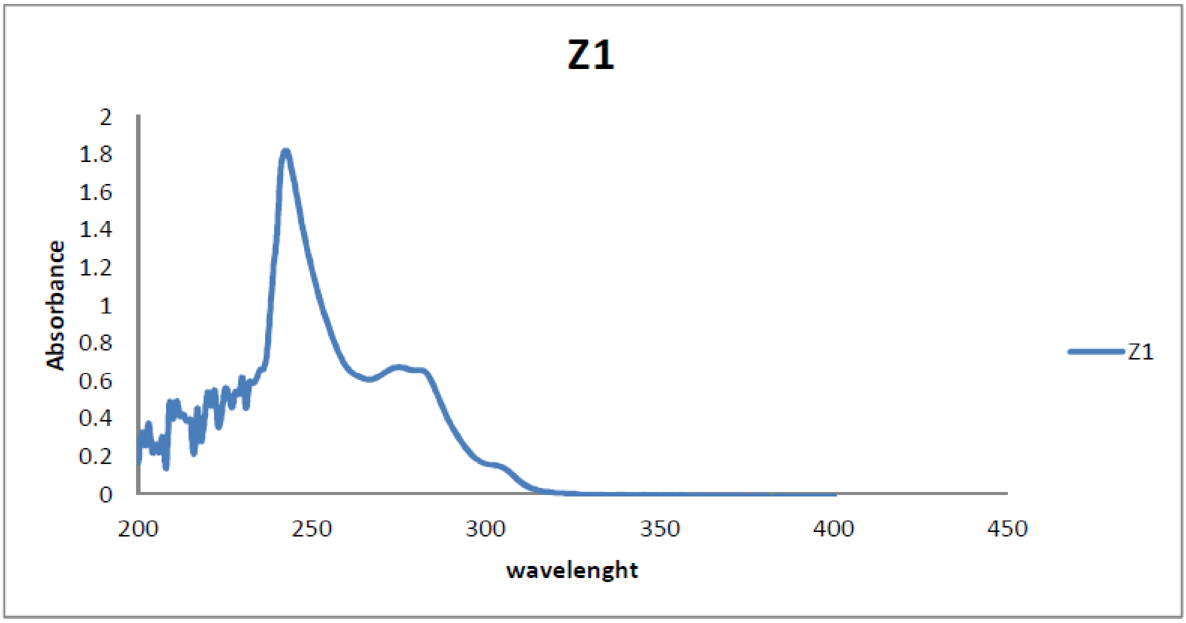

Appendix 1: UV spectrum of $\mathbf{Z_1}$

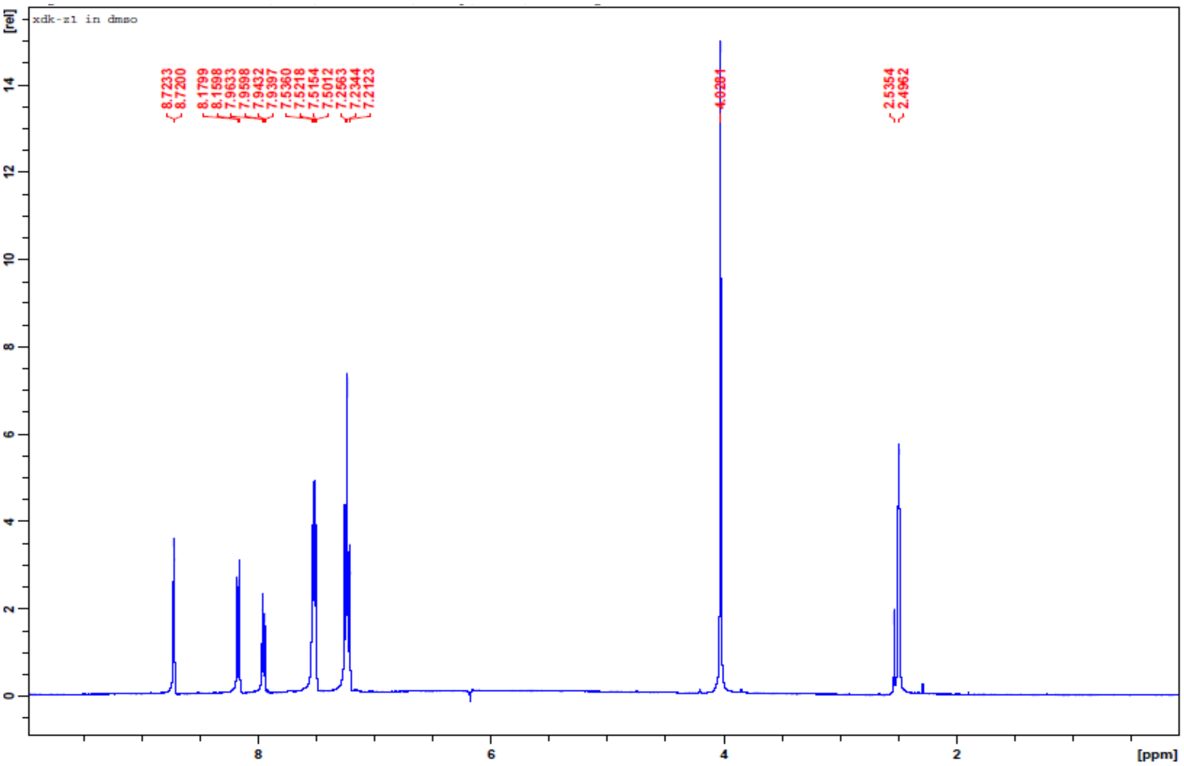

Appendix 2: ^{1}H-NMR spectrum of Z_1

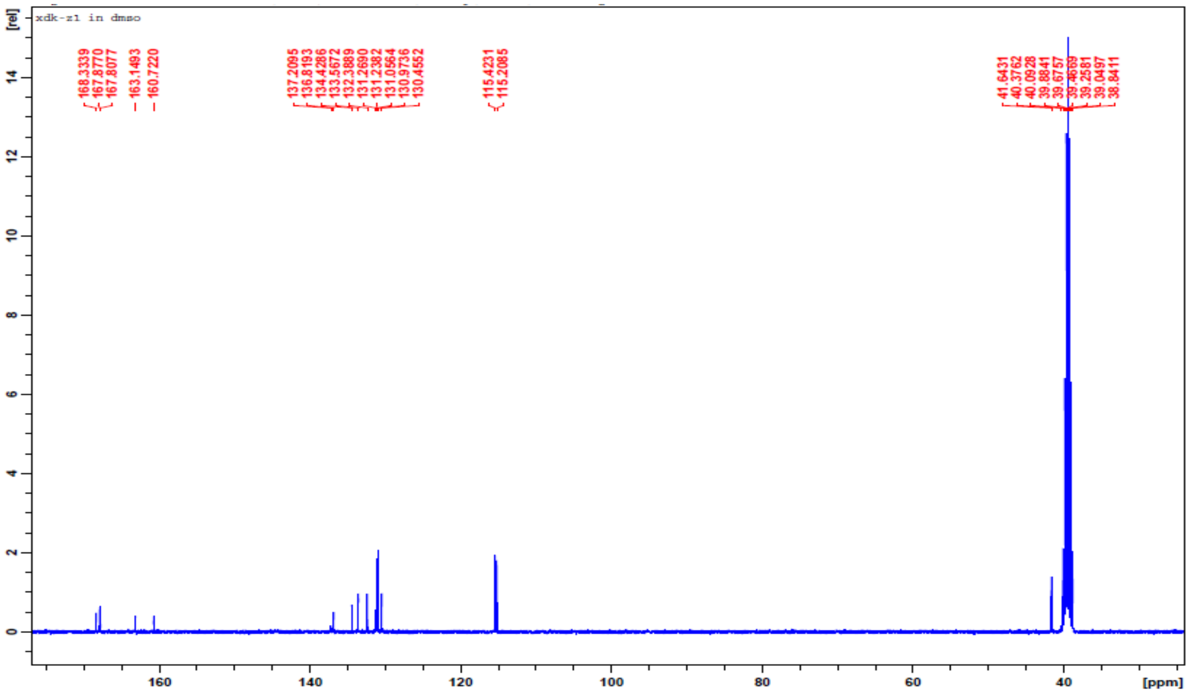

Appendix 3: ^{13}C-NMR spectrum of Z_1

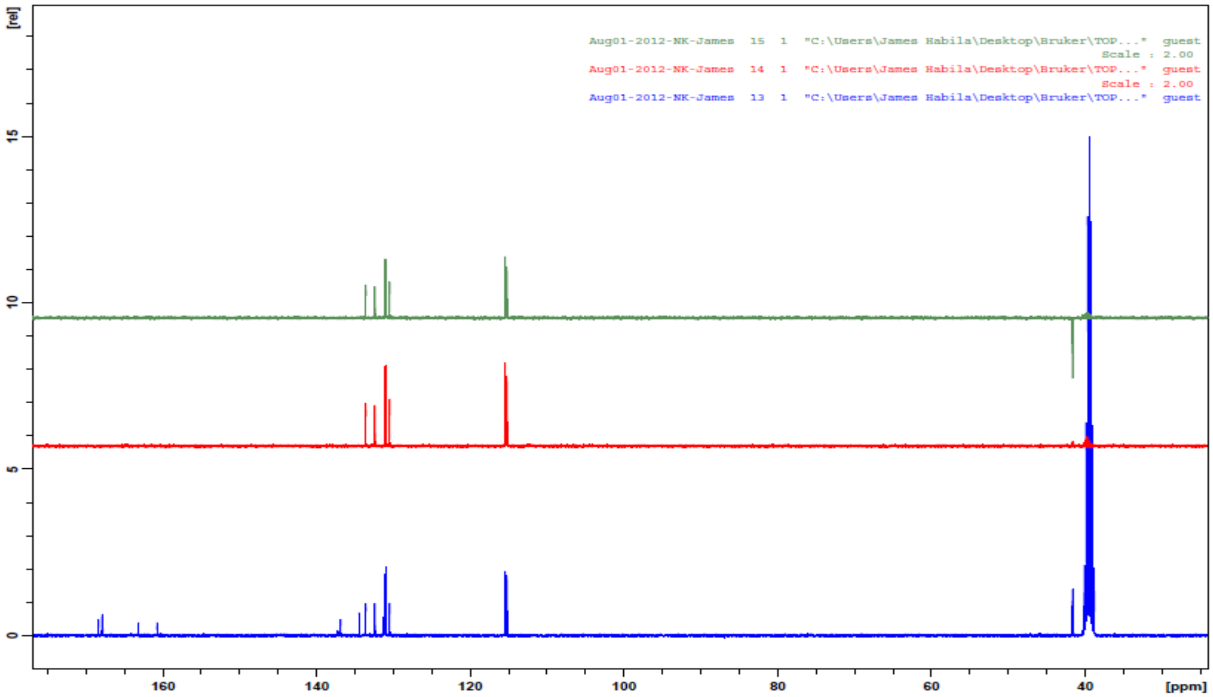

Appendix 4: DEPT spectrum of Z_1

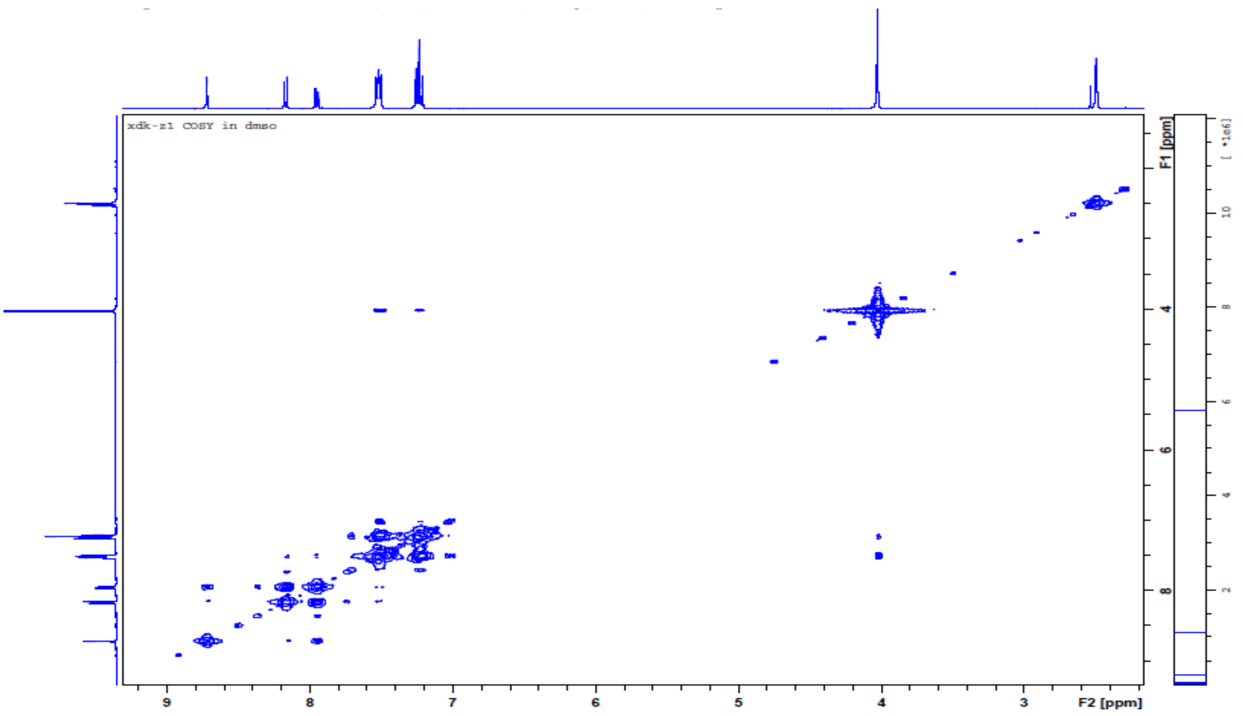

Appendix 5: COSY spectrum of $\mathbf{Z_1}$

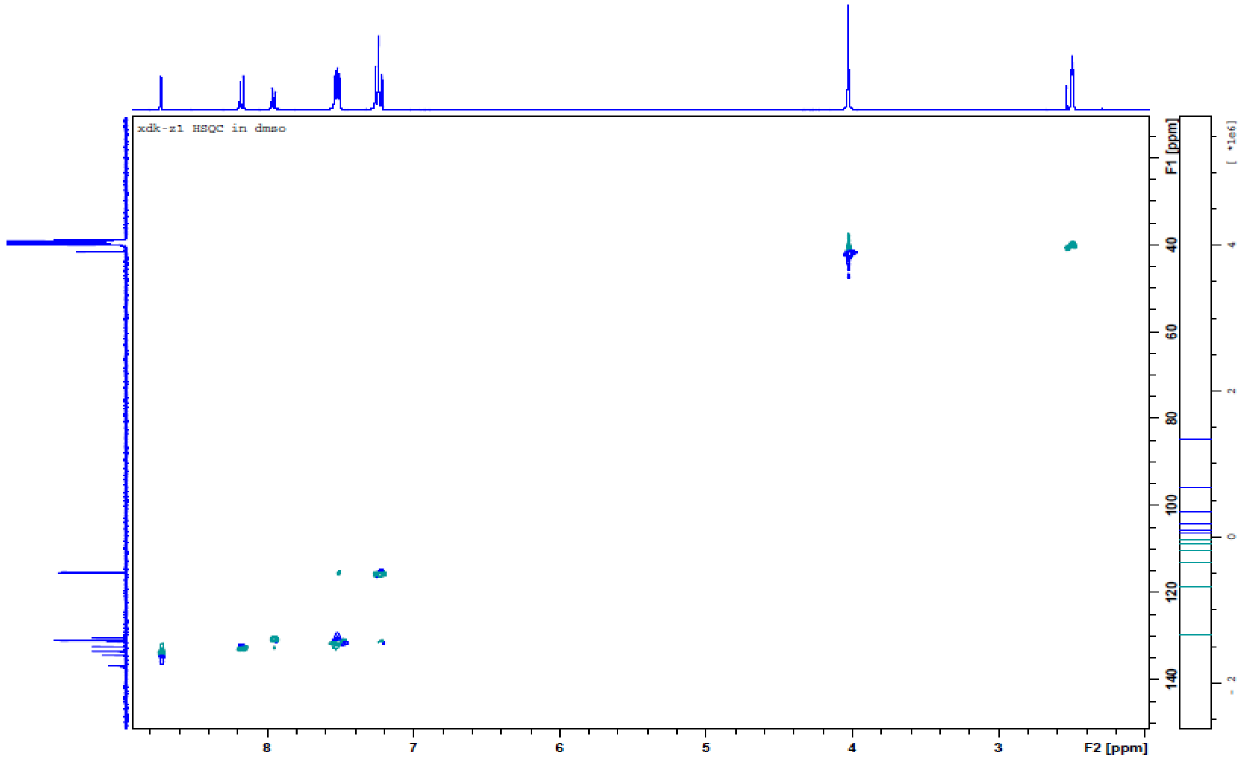

Appendix 6: HSQC spectrum of Z_1

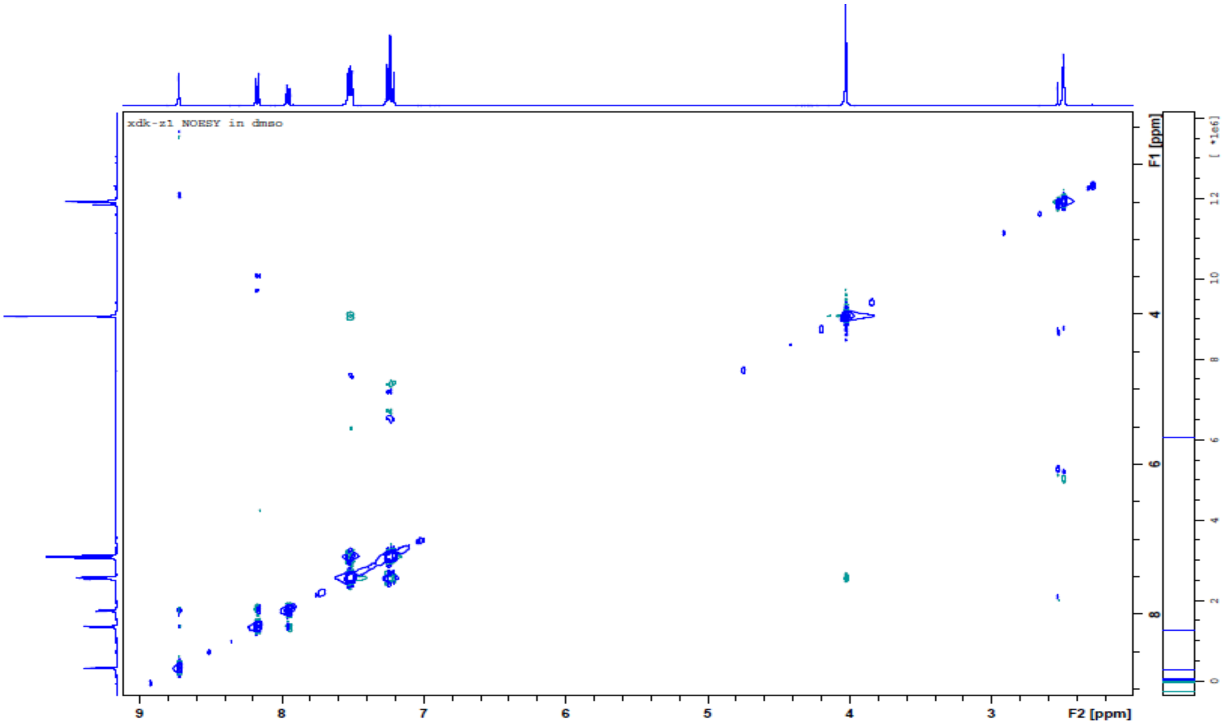

Appendix 7: NOESY spectrum of Z_1

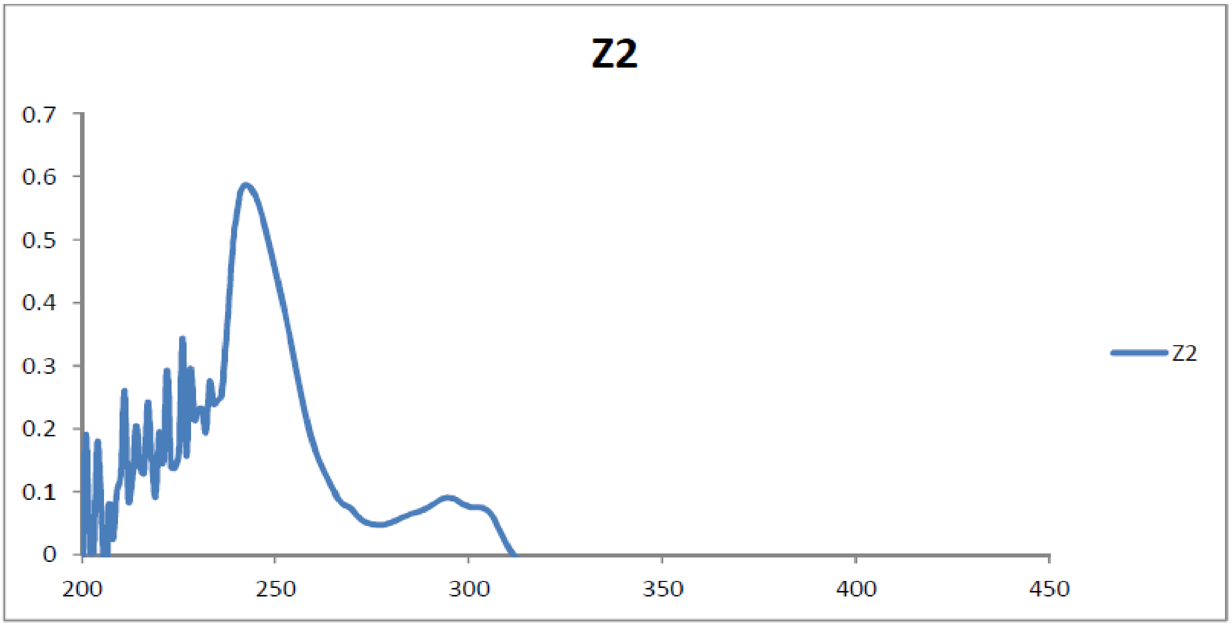

Appendix 8: UV spectrum of $\mathbf{Z_2}$

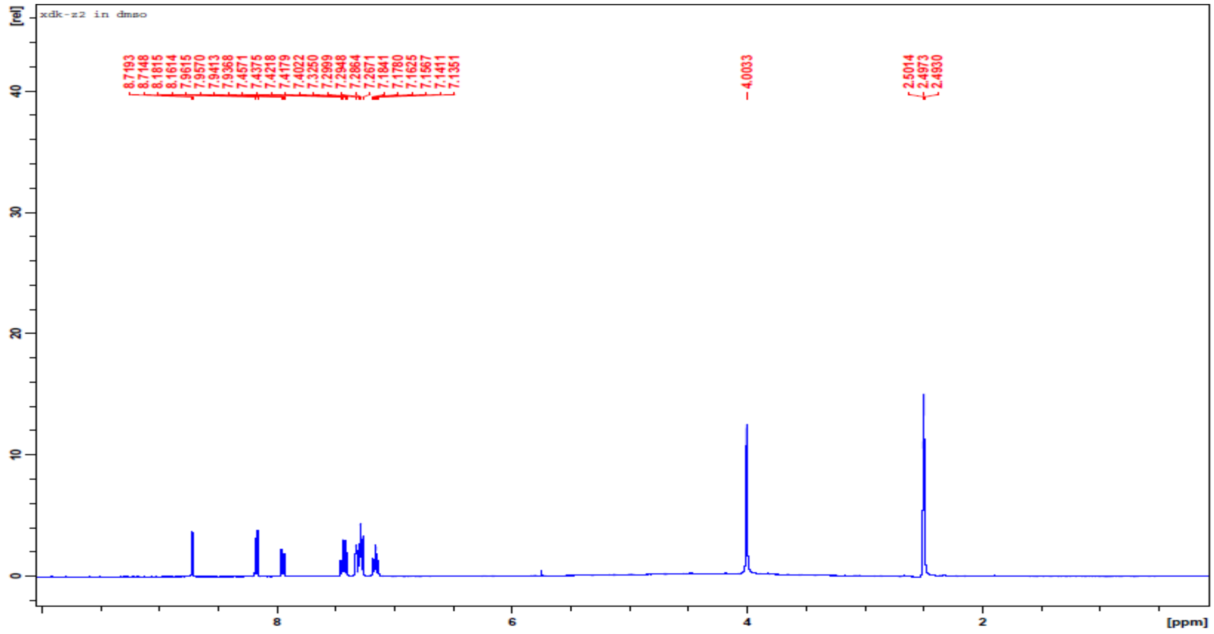

Appendix 9: ^{1}H-NMR spectrum of Z_2

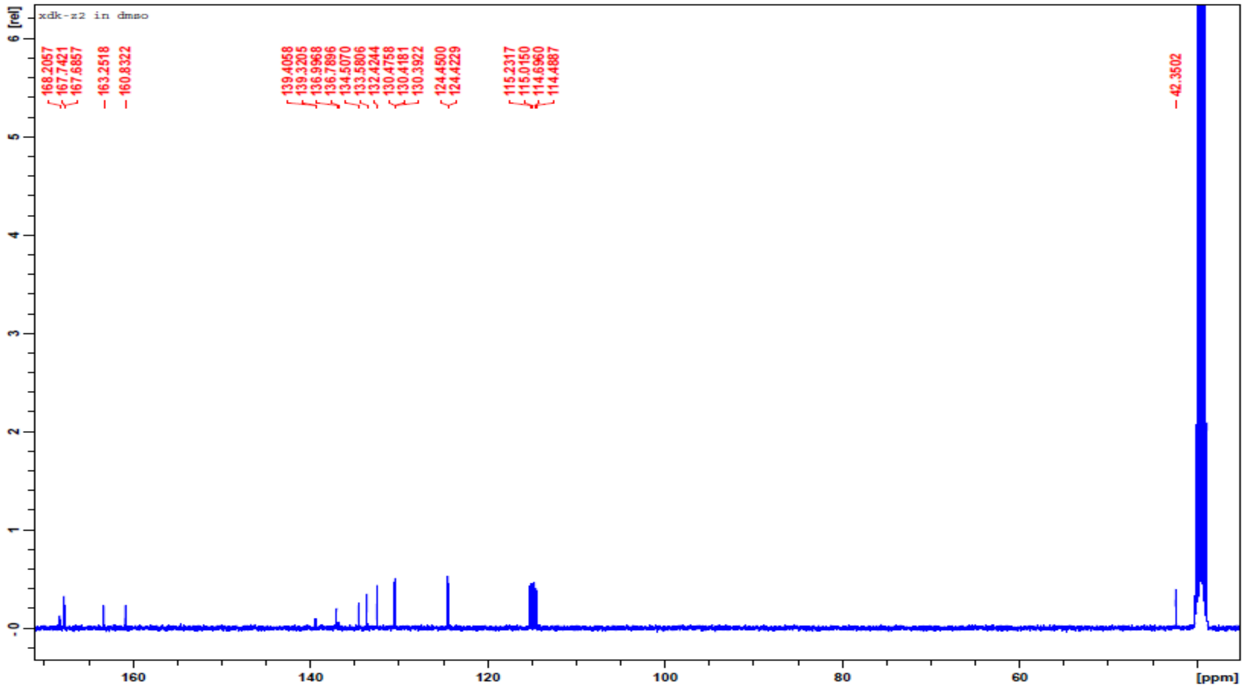

Appendix 10: ^{13}C-NMR spectrum of Z_2

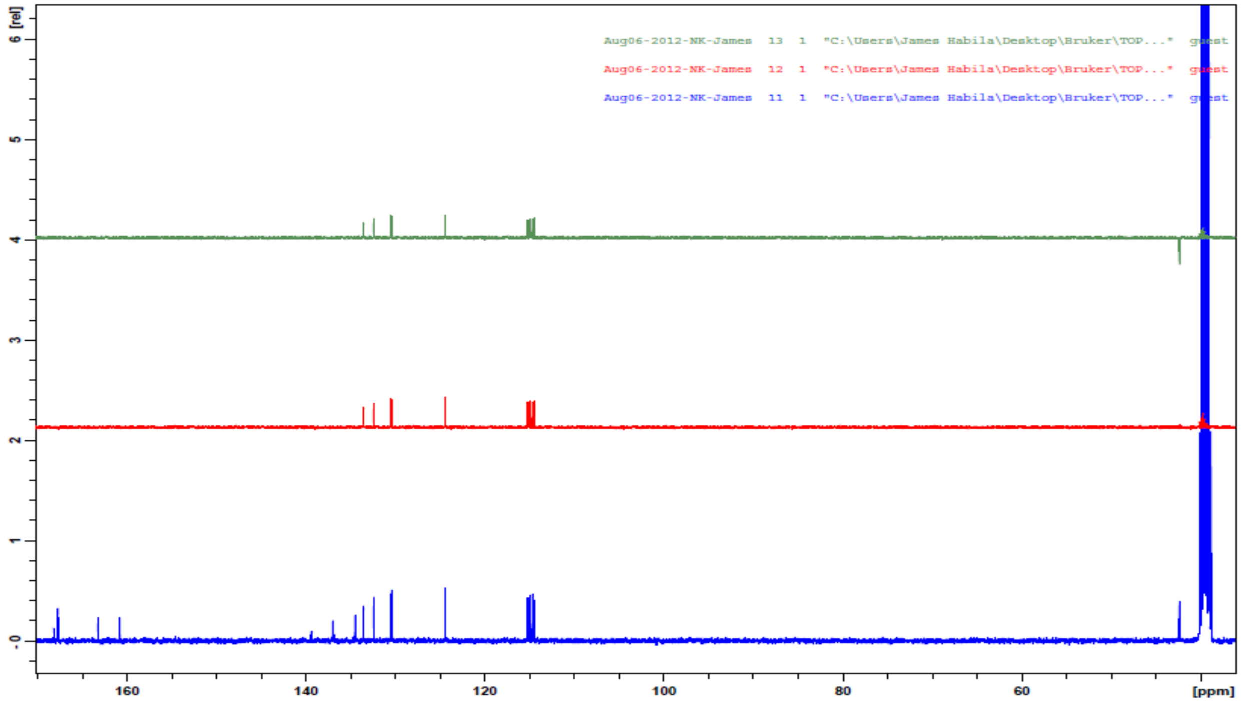

Appendix11: DEPT spectrum of Z_2

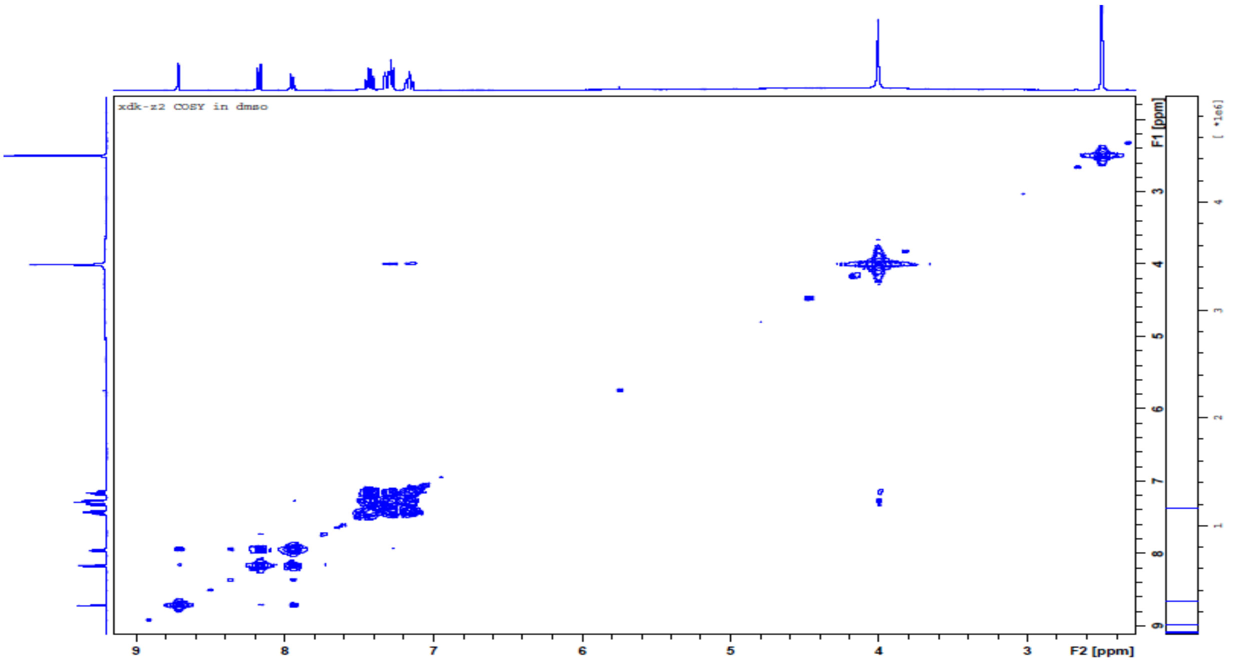

Appendix 12: COSY spectrum of Z_2

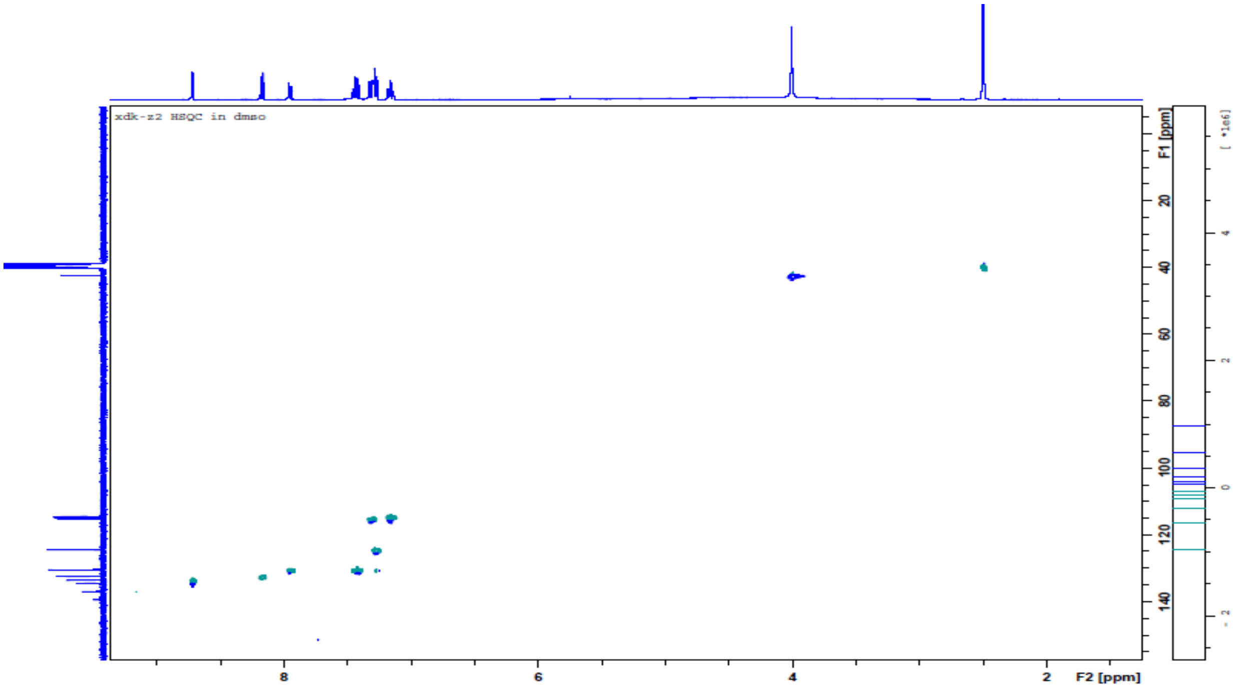

Appendix 13: HSQC spectrum of Z_2

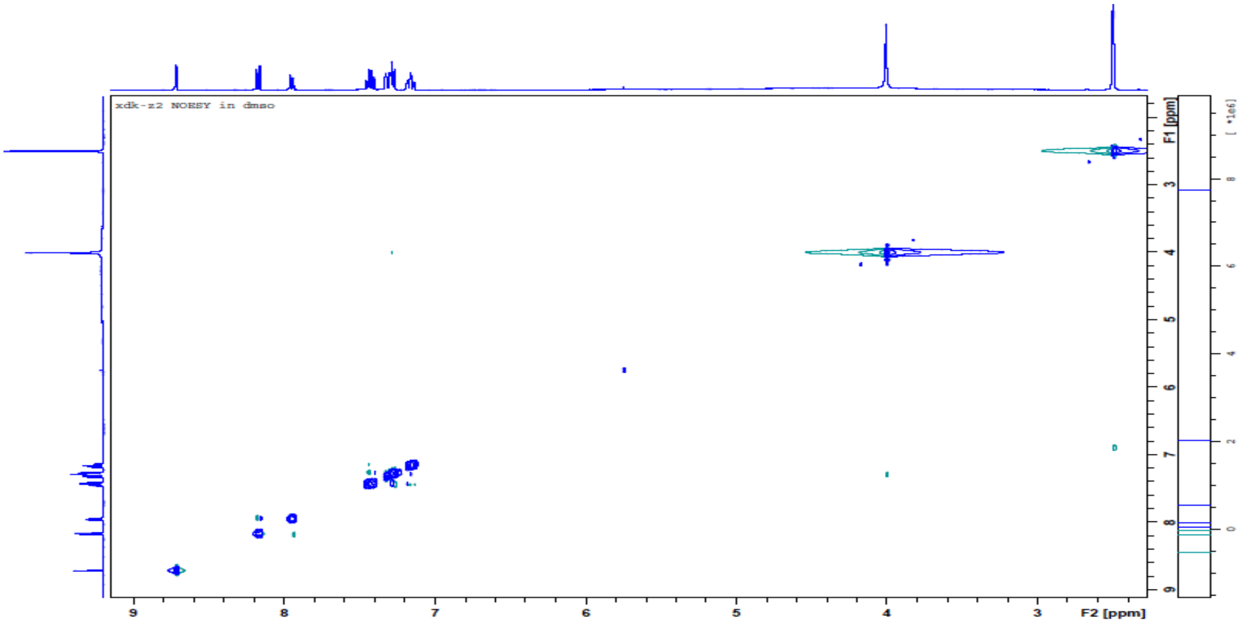

Appendix 14: NOESY spectrum of Z_2

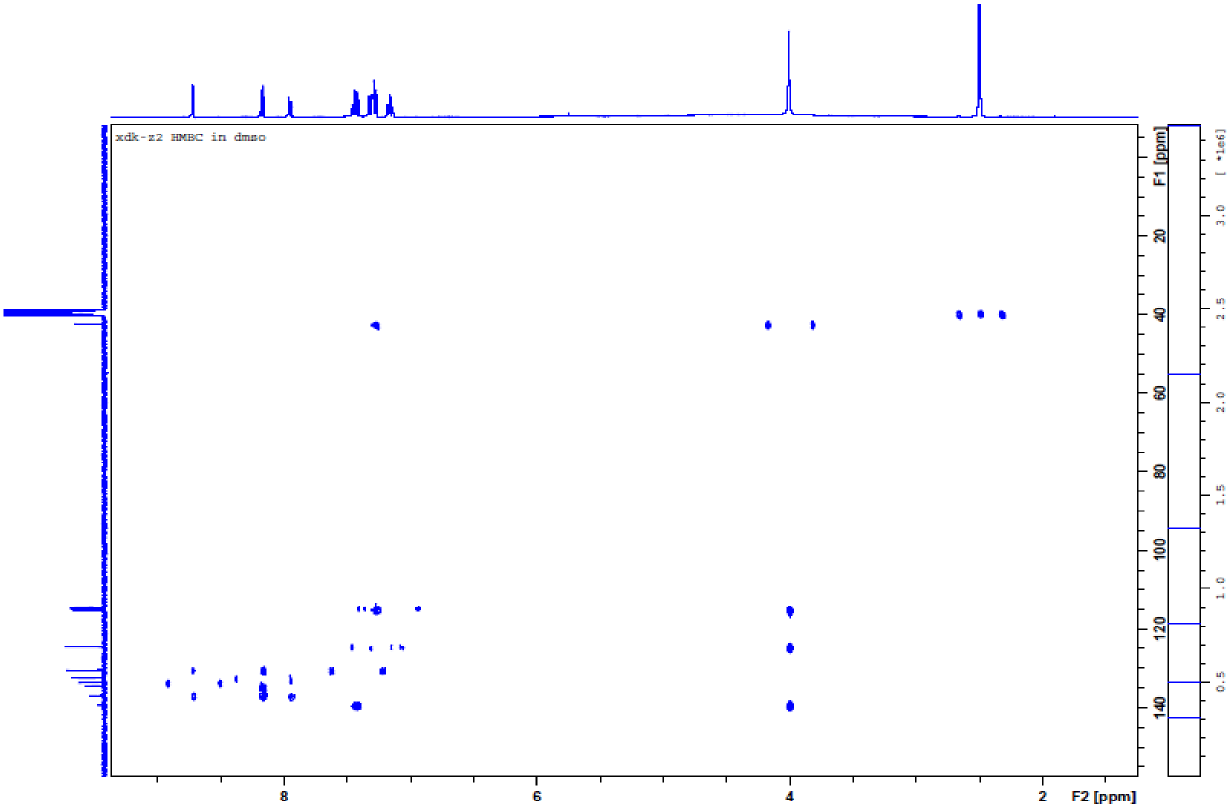

Appendix 15: HMBC spectrum of Z_2

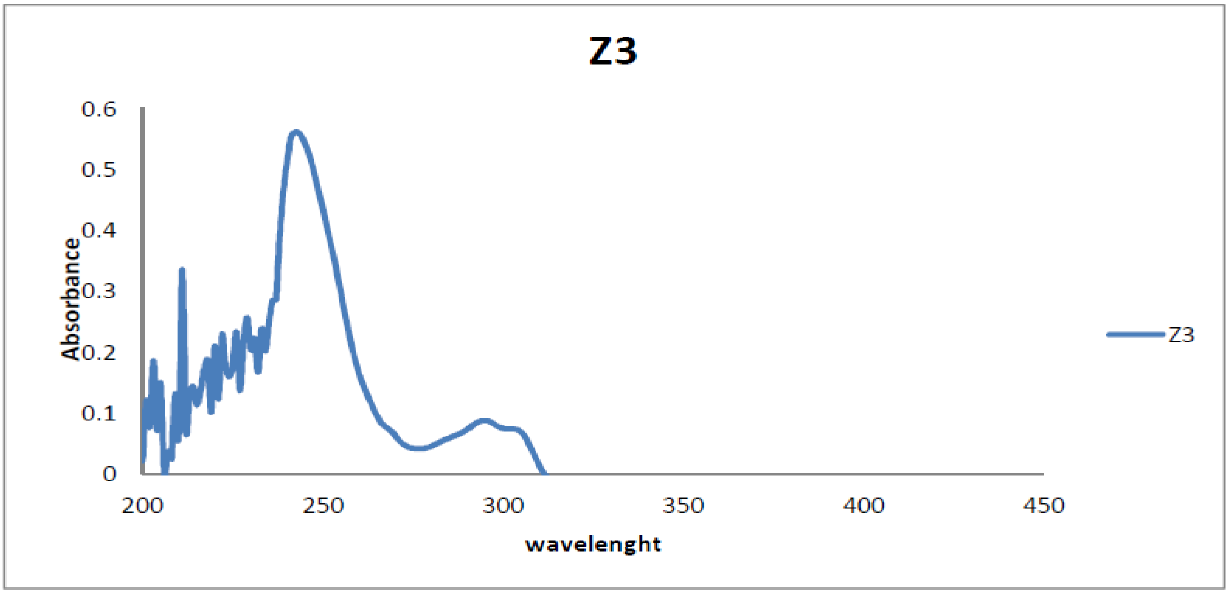

Appendix 16: UV Spectrum of Z_3

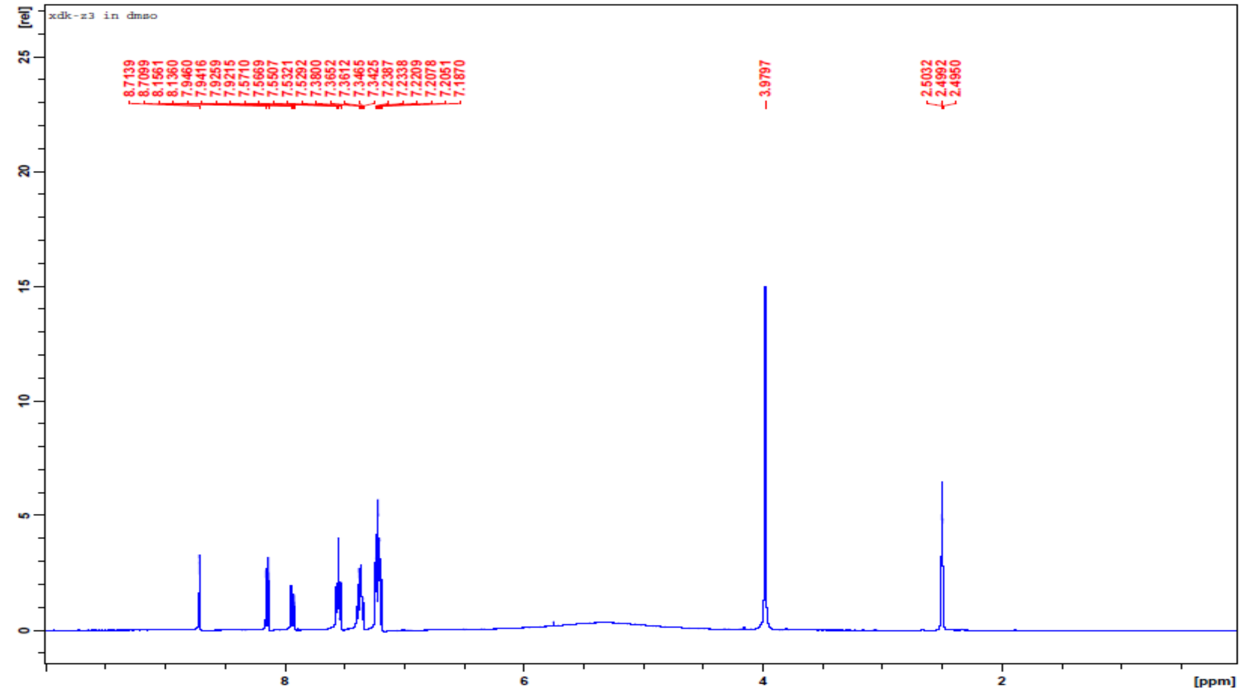

Appendix 17; ^{1}HNMR Spectrum of Z_3

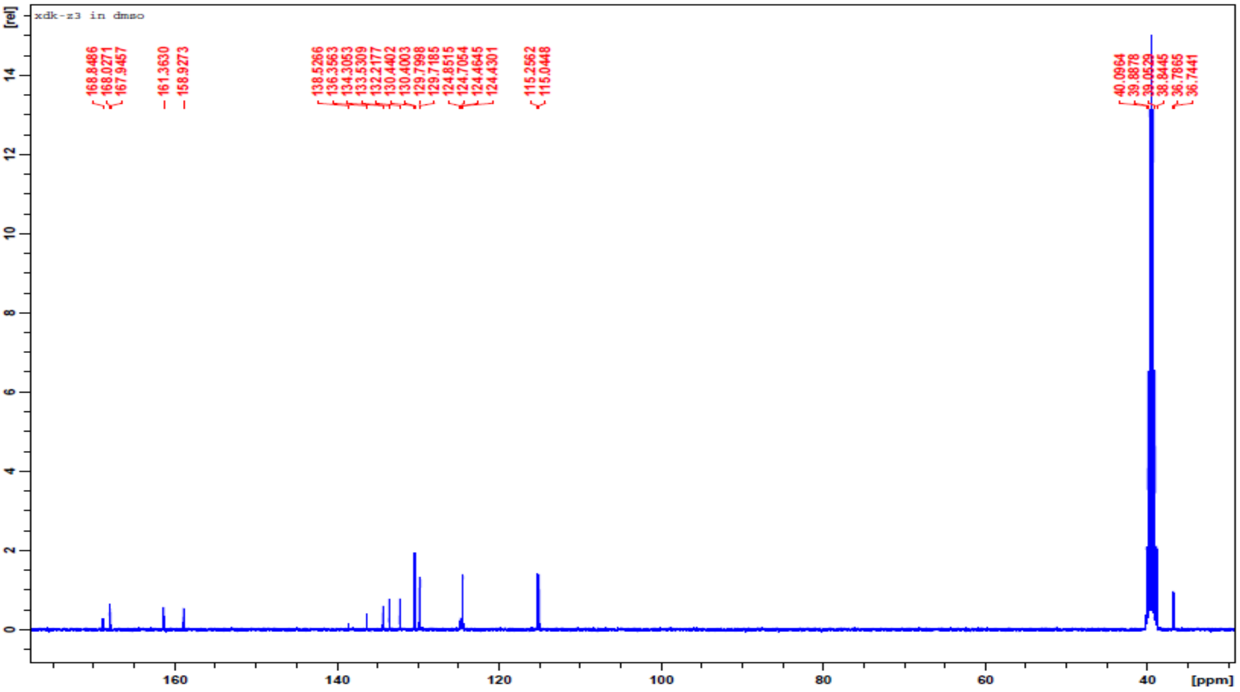

Appendix 18: ^{13}C NMR Spectrum of Z_3

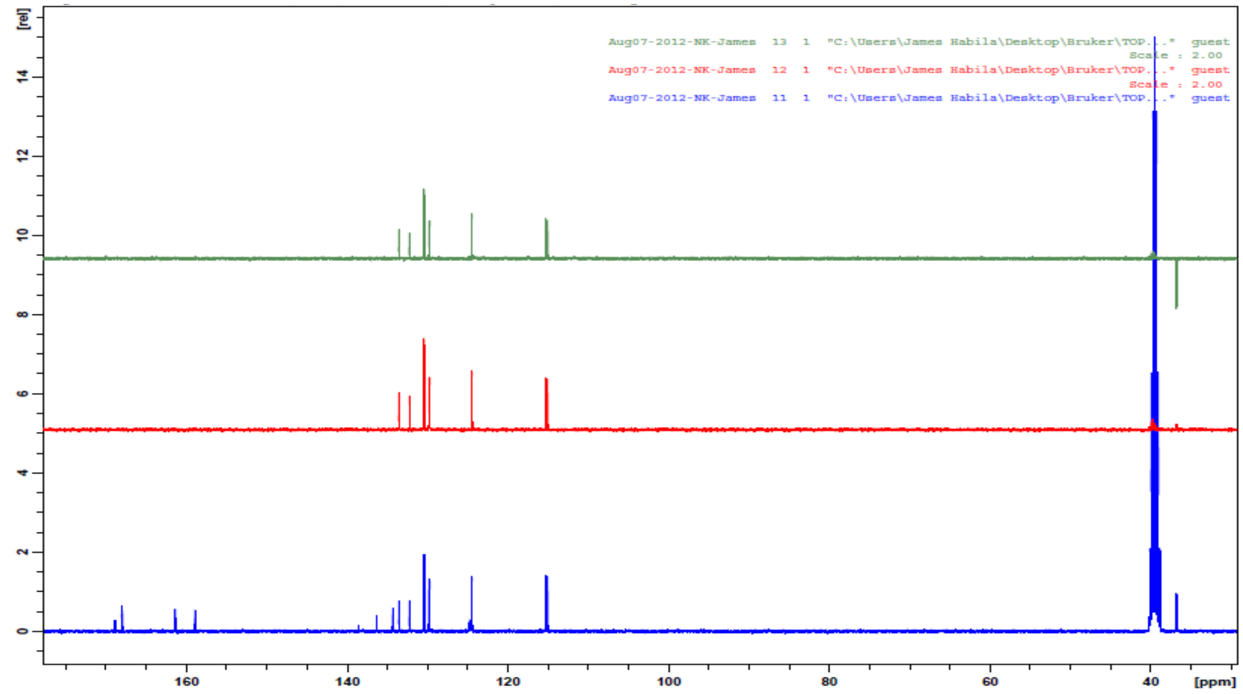

Appendix19: DEPT Spectrum of Z_3

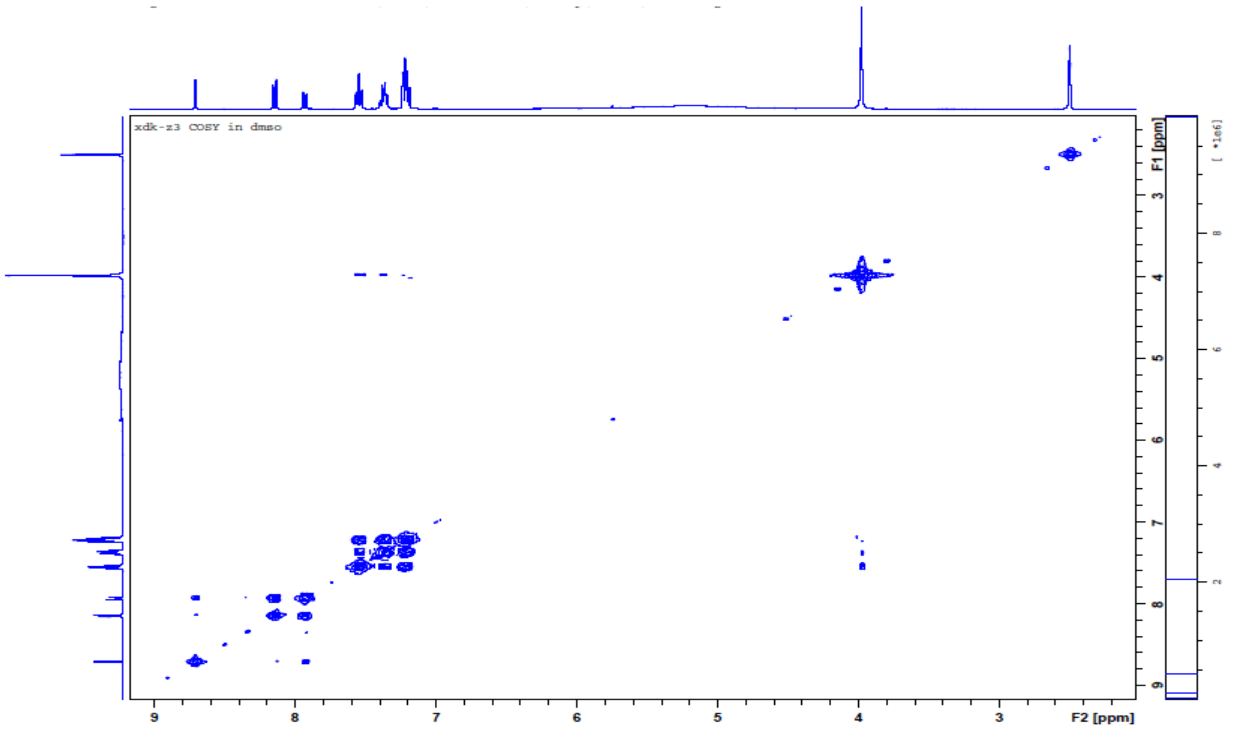

Appendix 20: COSY spectrum of Z_3

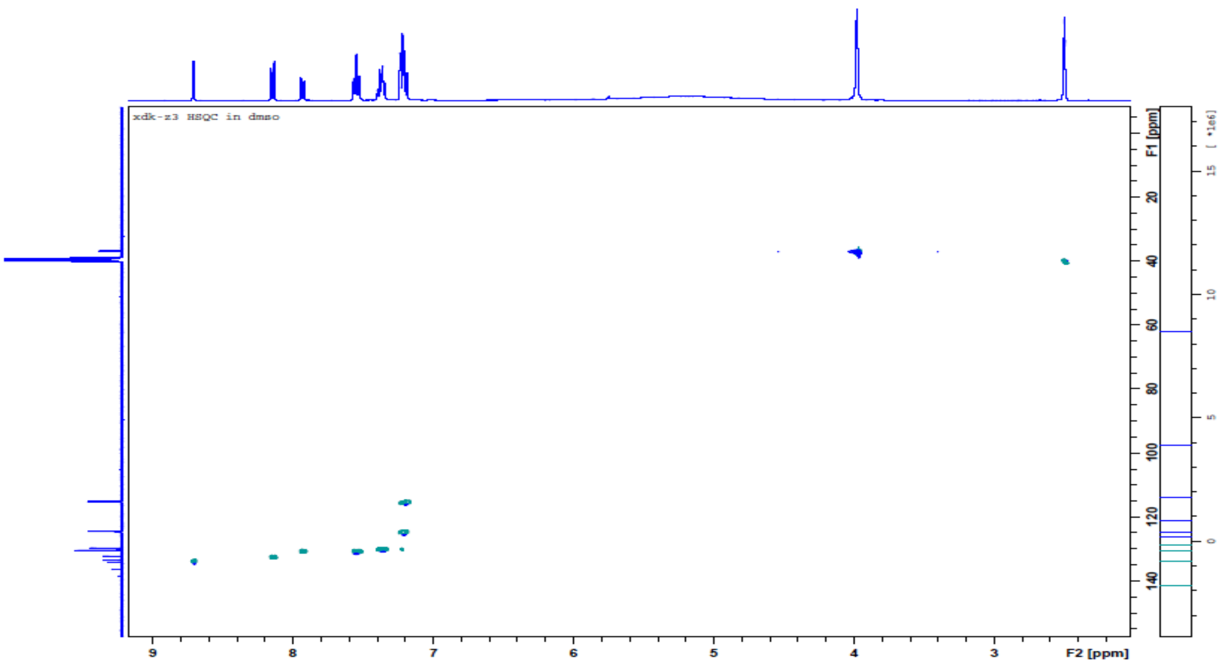

Appendix 21: HSQC spectrum of Z_3

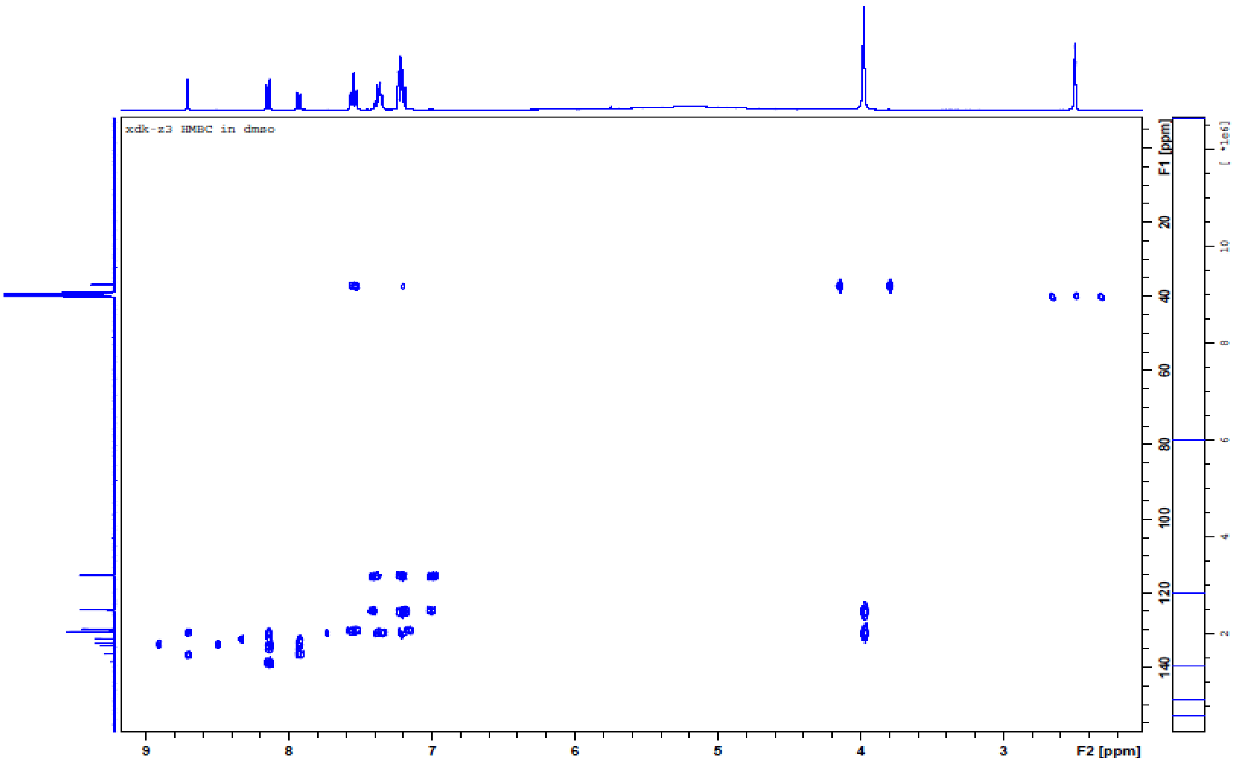

Appendix 22: HMBC spectrum of Z_3

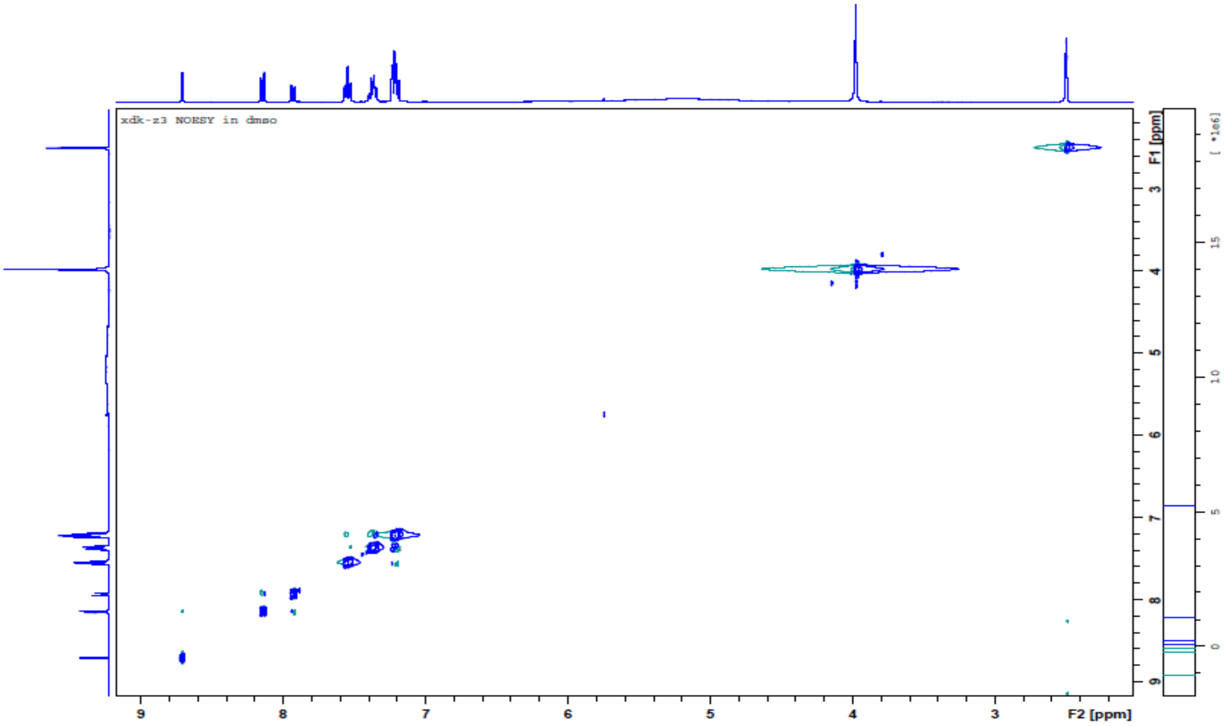

Appendix 23: NOESY spectrum of Z_3

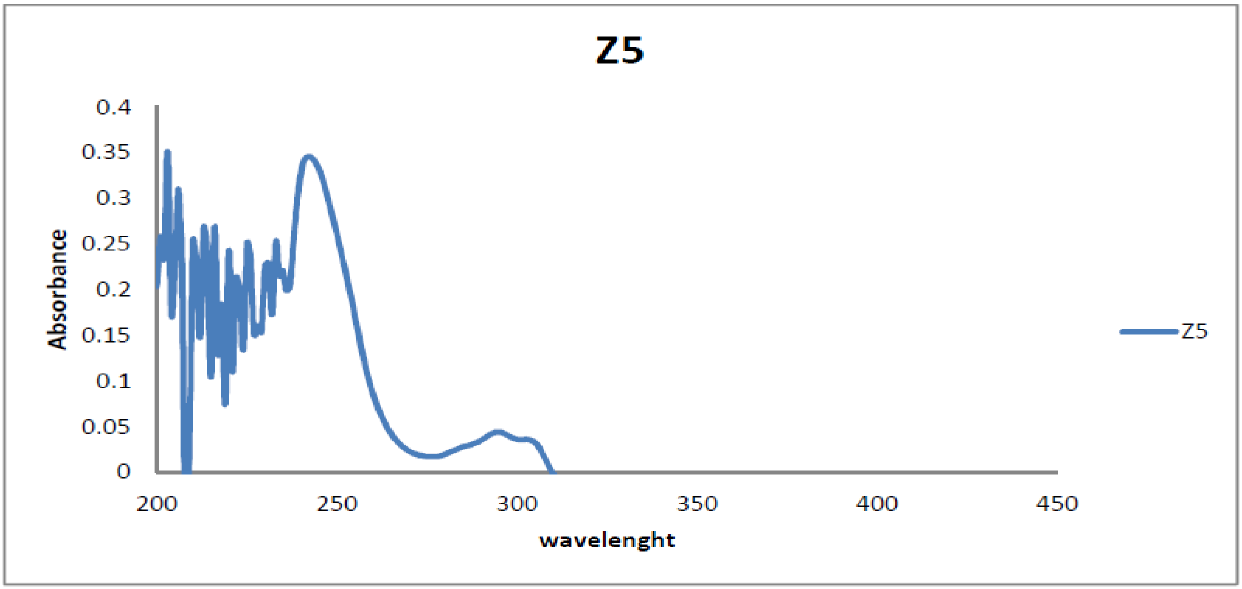

Appendix 24: UV Spectrum of Z_5

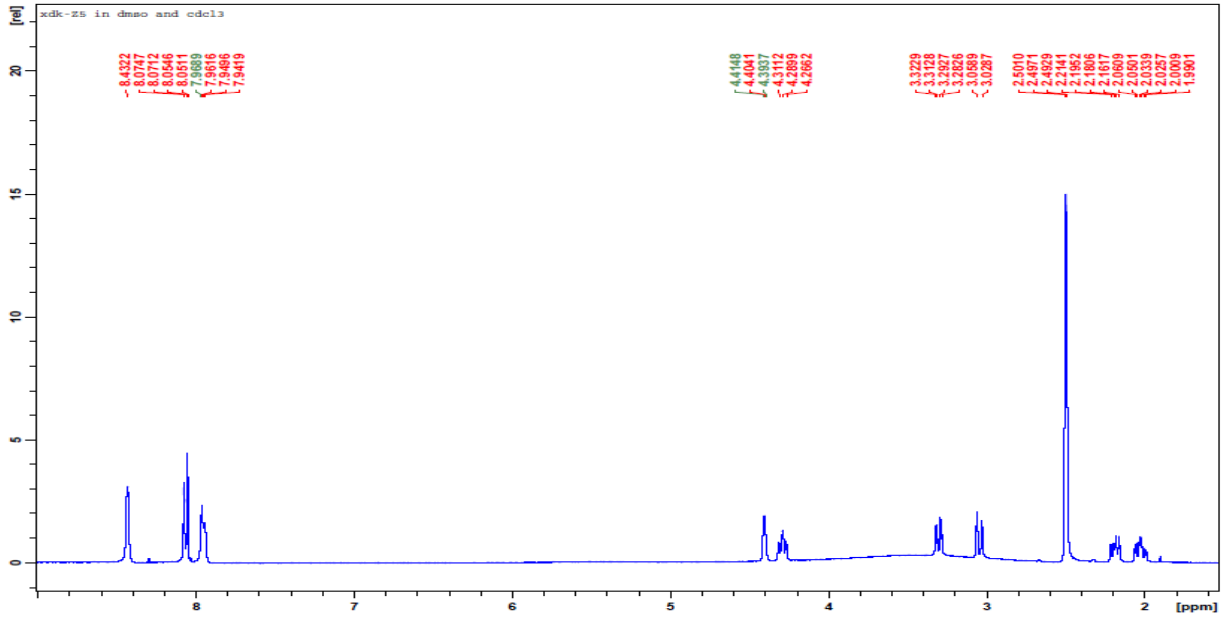

Appendix 25: ^{1}H NMR spectrum of Z_5

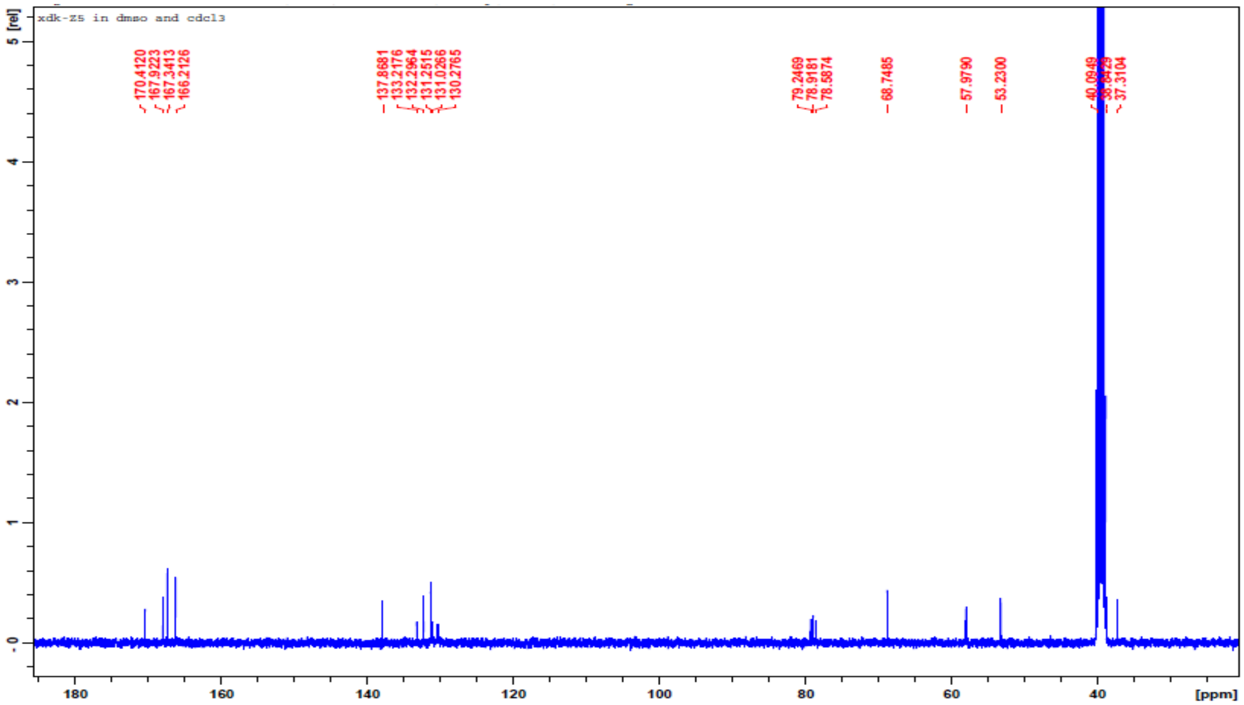

Appendix 26: ^{13}C NMR Spectrum of Z_5

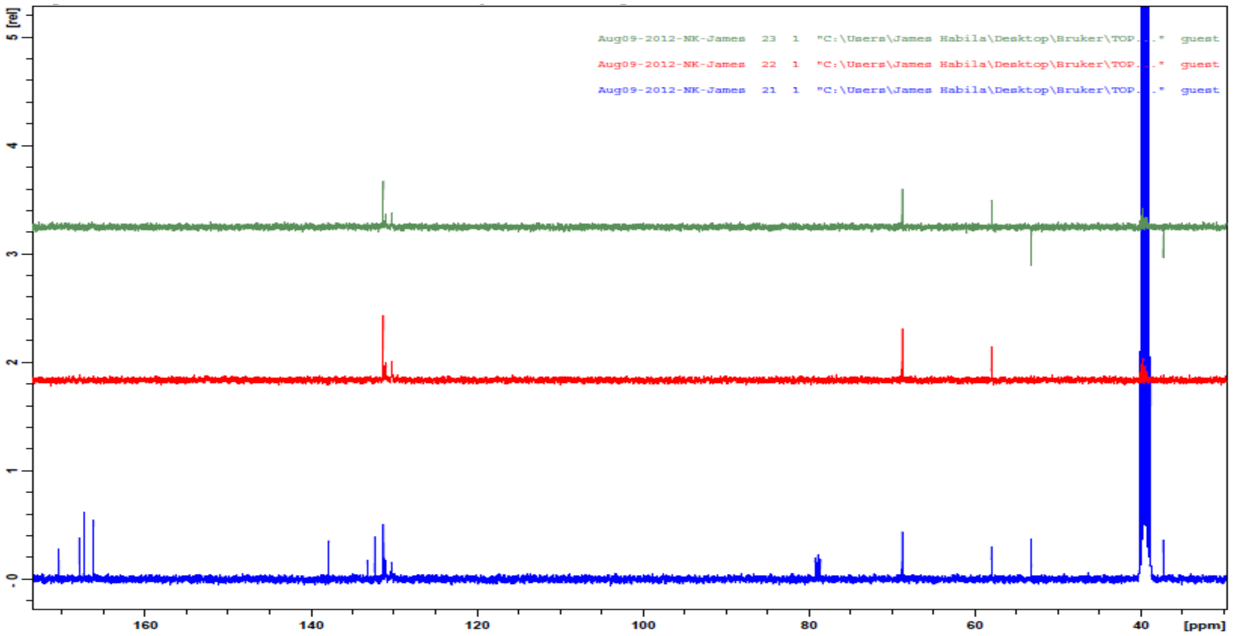

Appendix 27: DEPT spectrum of Z_5

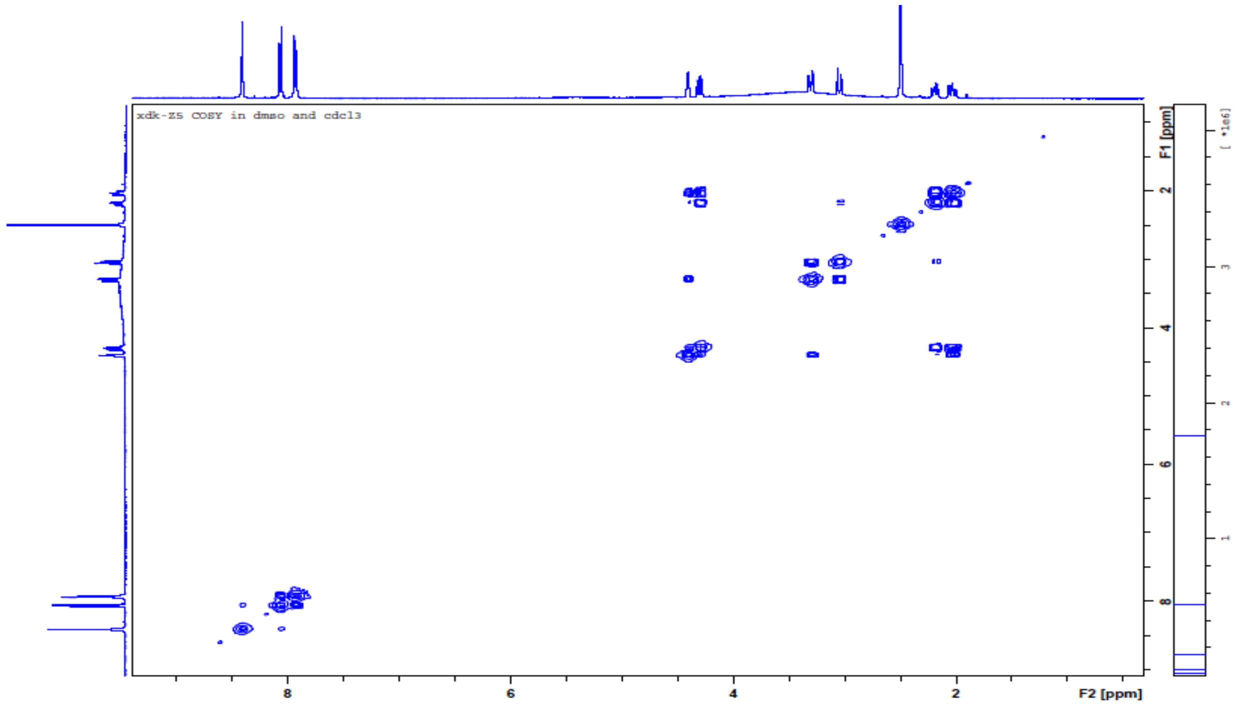

Appendix 28: COSY spectrum of Z_5

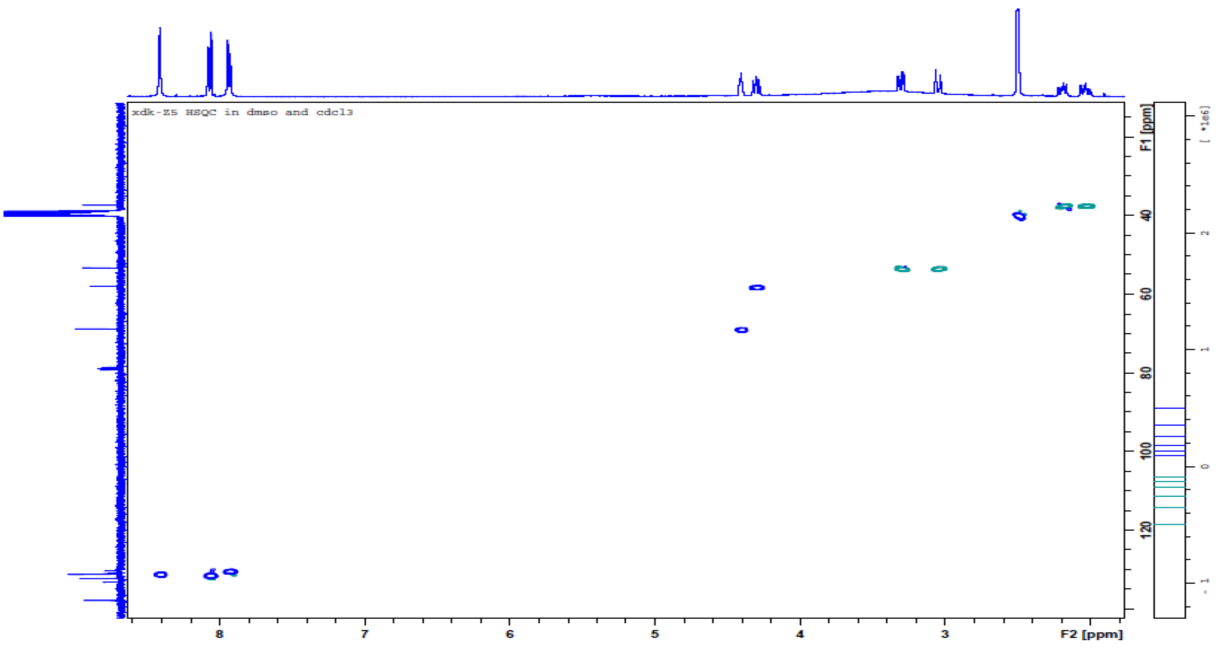

Appendix 29: HSQC spectrum of Z_3

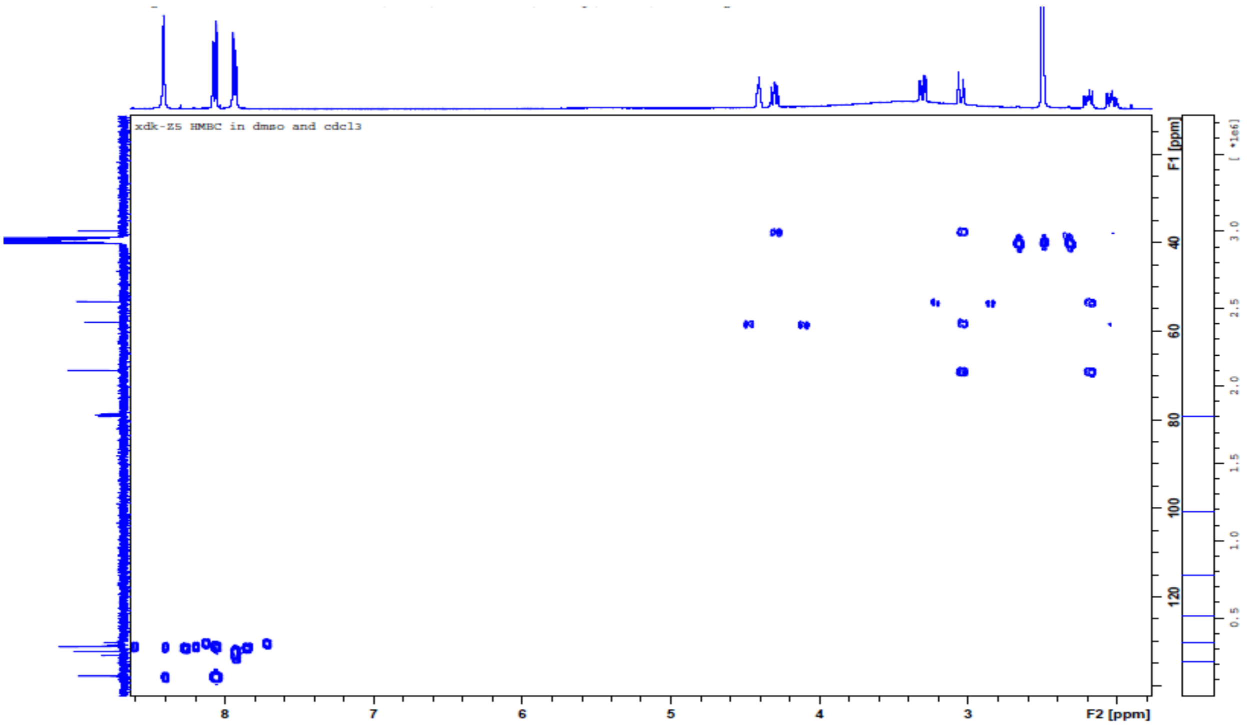

Appendix 30: HMBC spectrum of Z_5

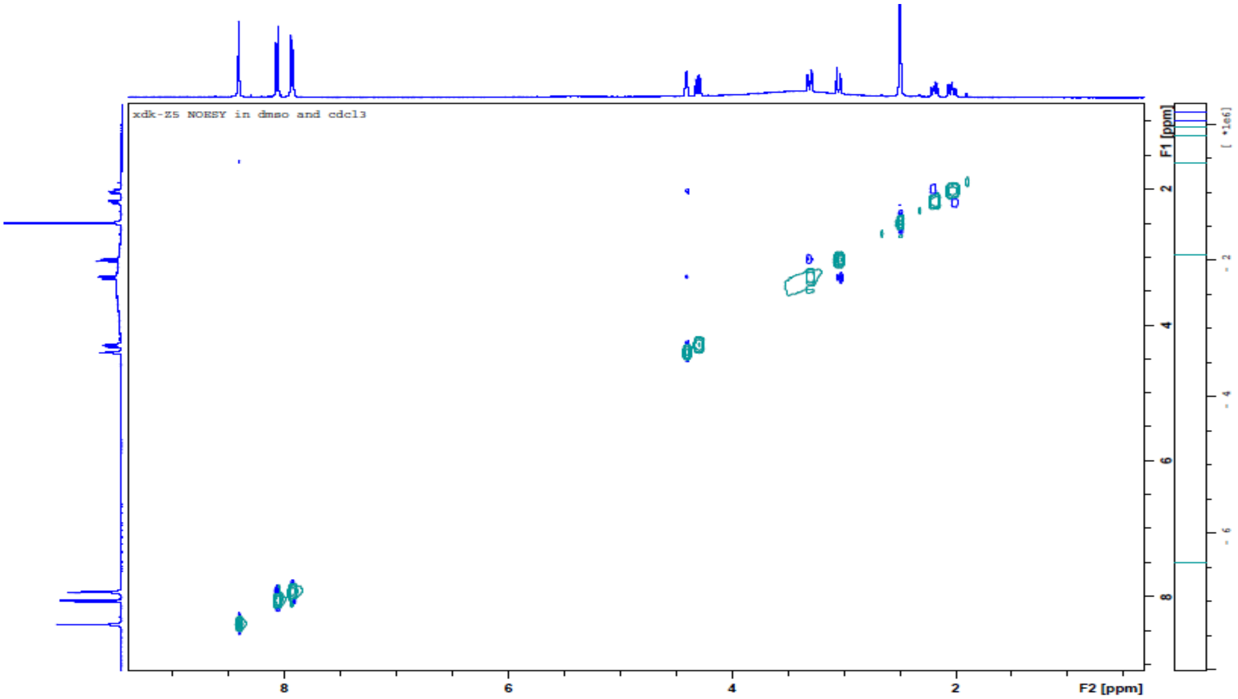

Appendix 31: NOESY spectrum of Z_5

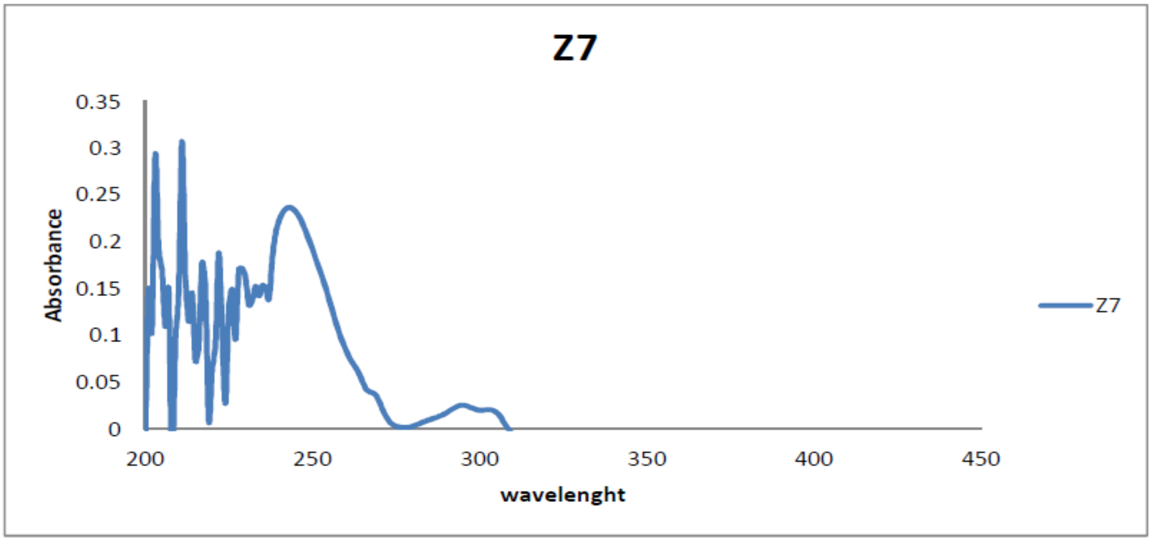

Appendix 32: UV spectrum of Z_7

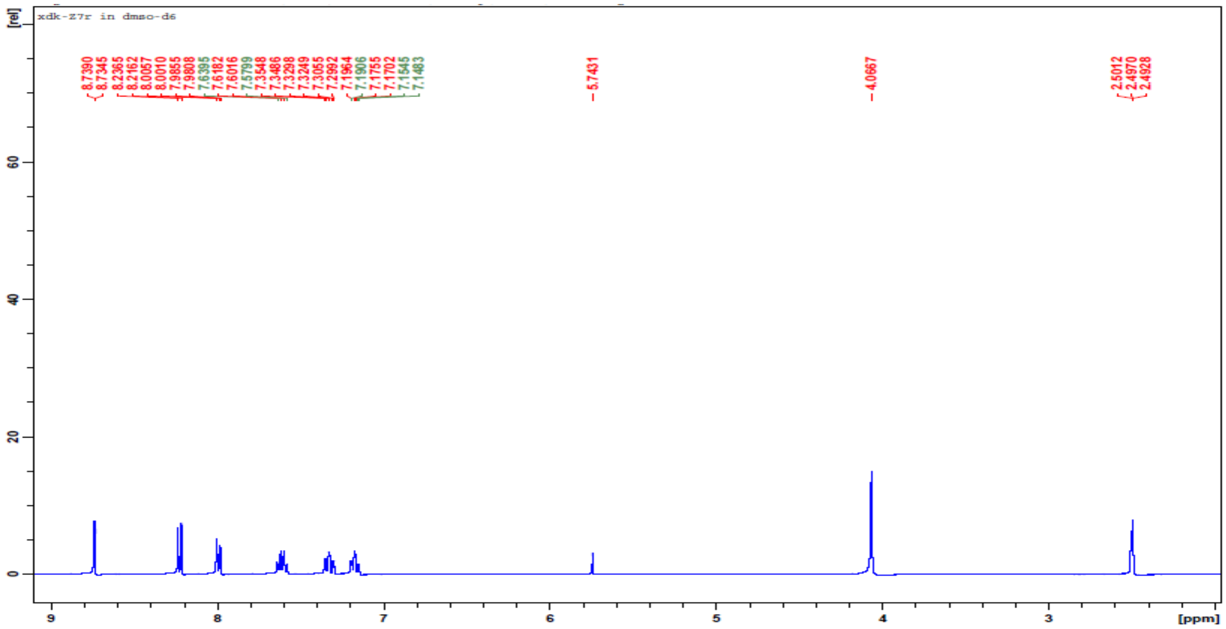

Appendix 33: ^{1}H NMR spectrum of Z_7

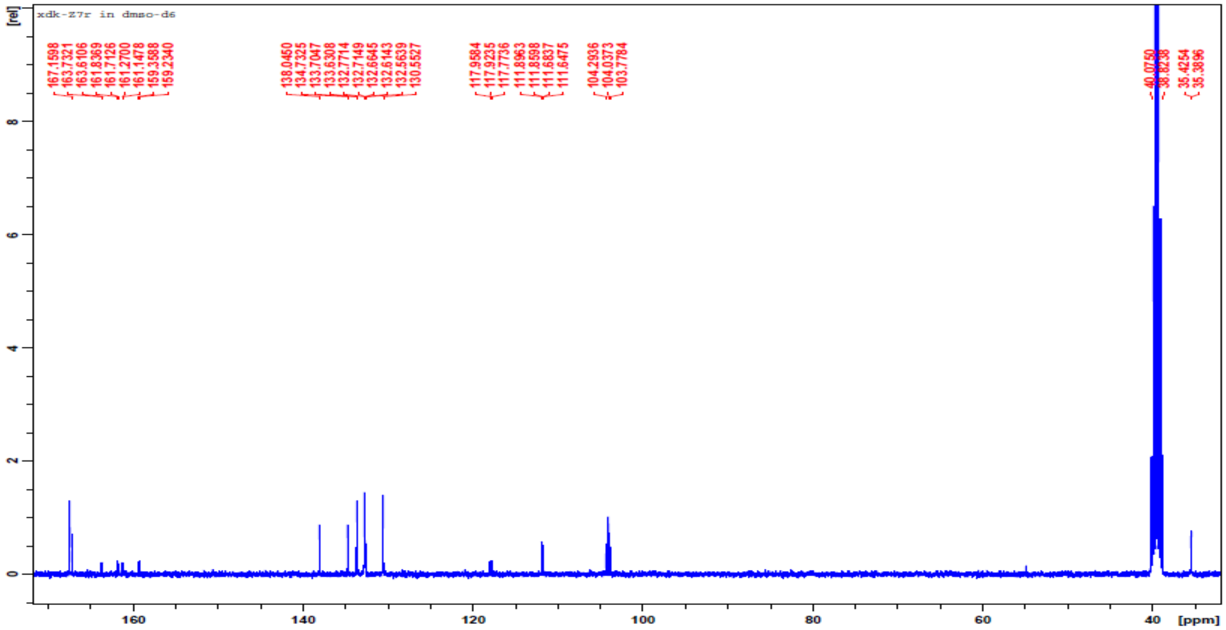

Appendix 34: ^{13}C NMR spectrum of Z_7

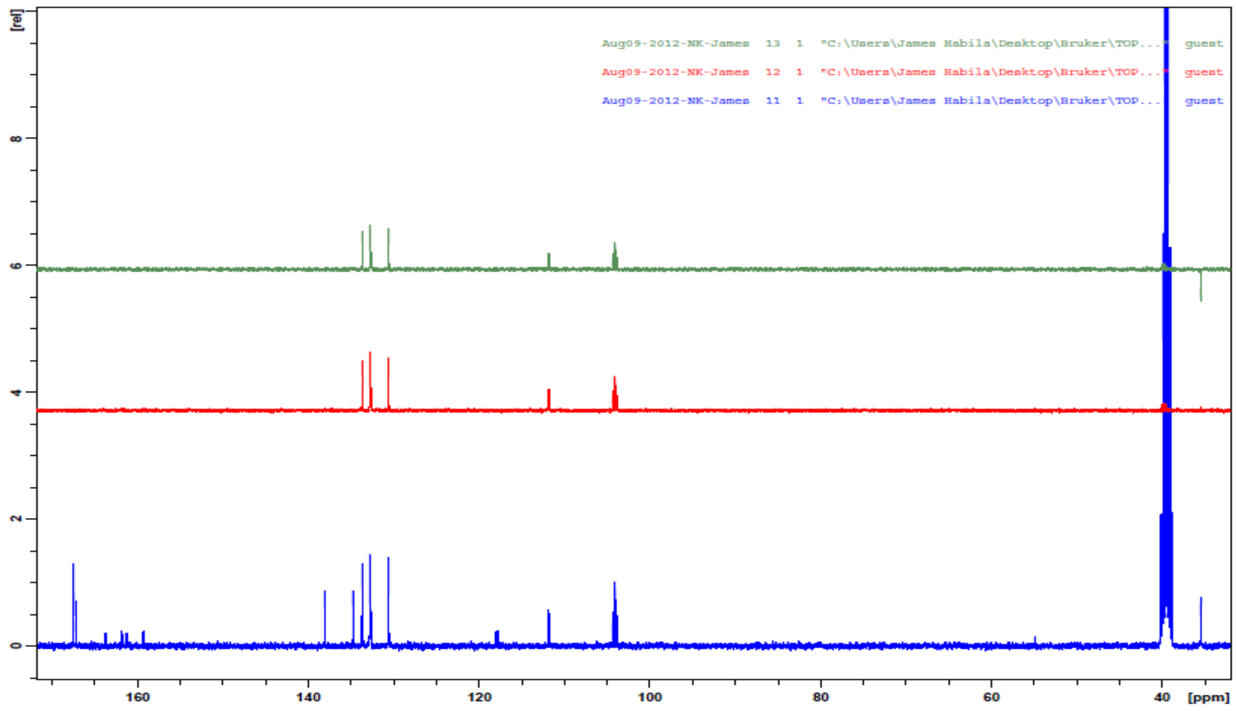

Appendix 35: DEPT spectrum of Z_7

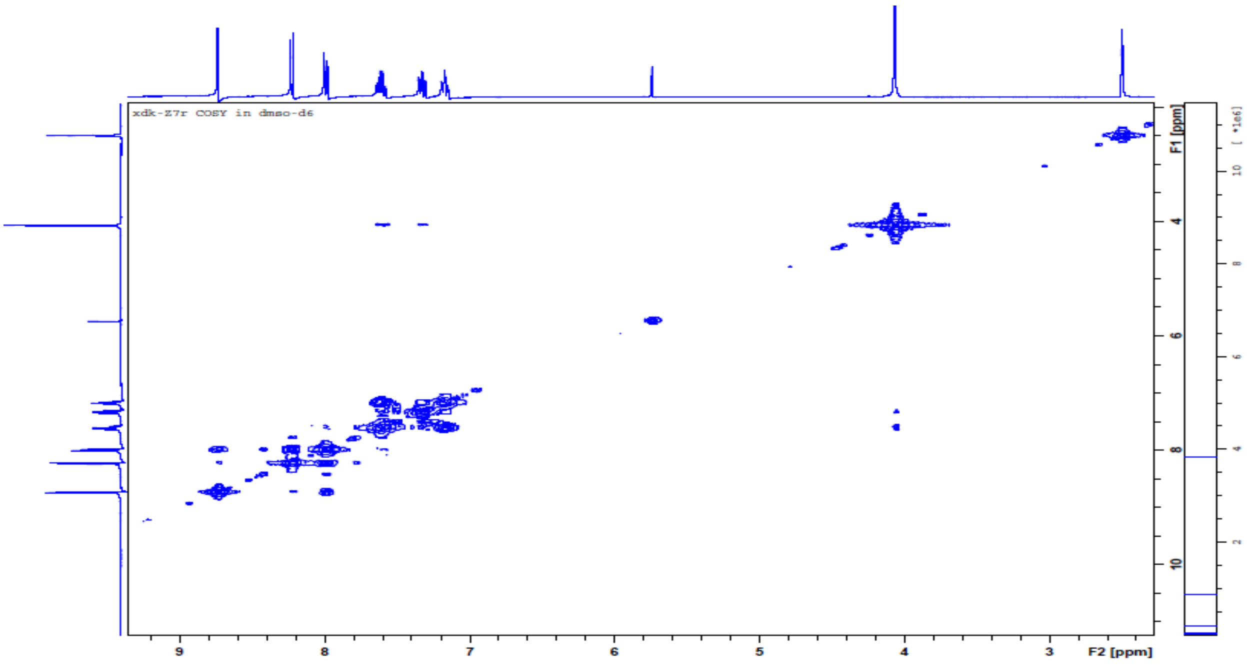

Appendix 36: COSY spectrum of Z_7

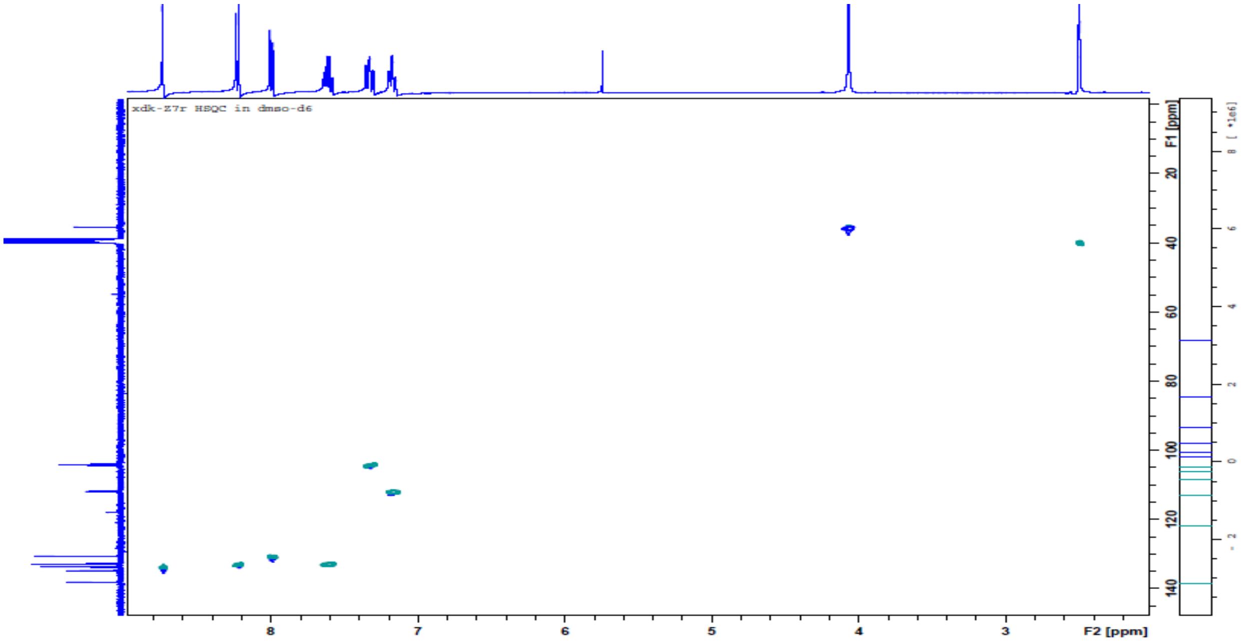

Appendix 37: HSQC spectrum of Z_7

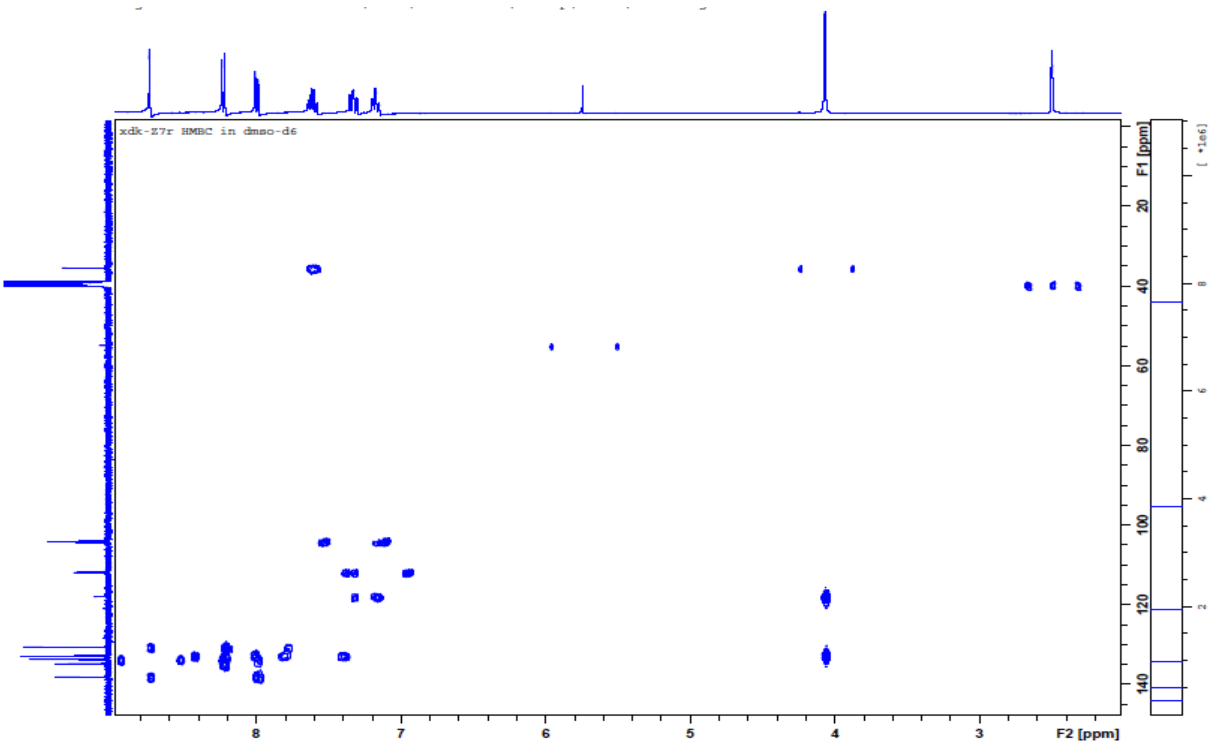

Appendix 38: HMBC spectrum of Z_7

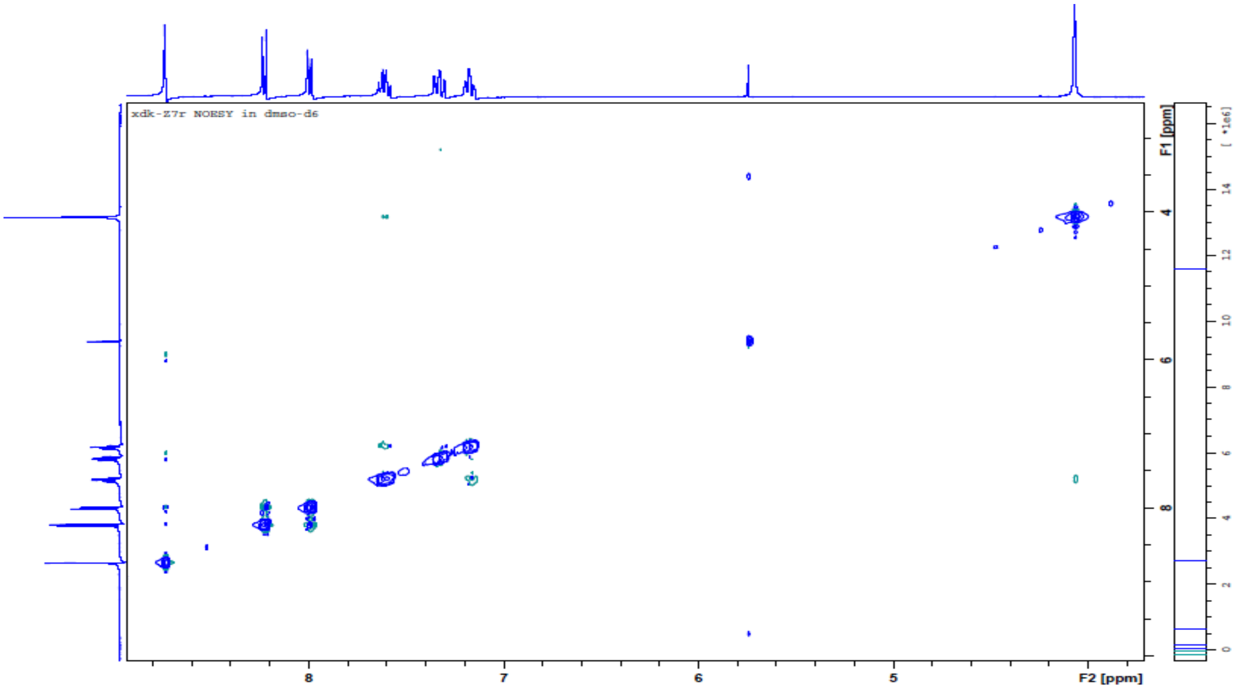

Appendix 39: NOESY spectrum of Z_7

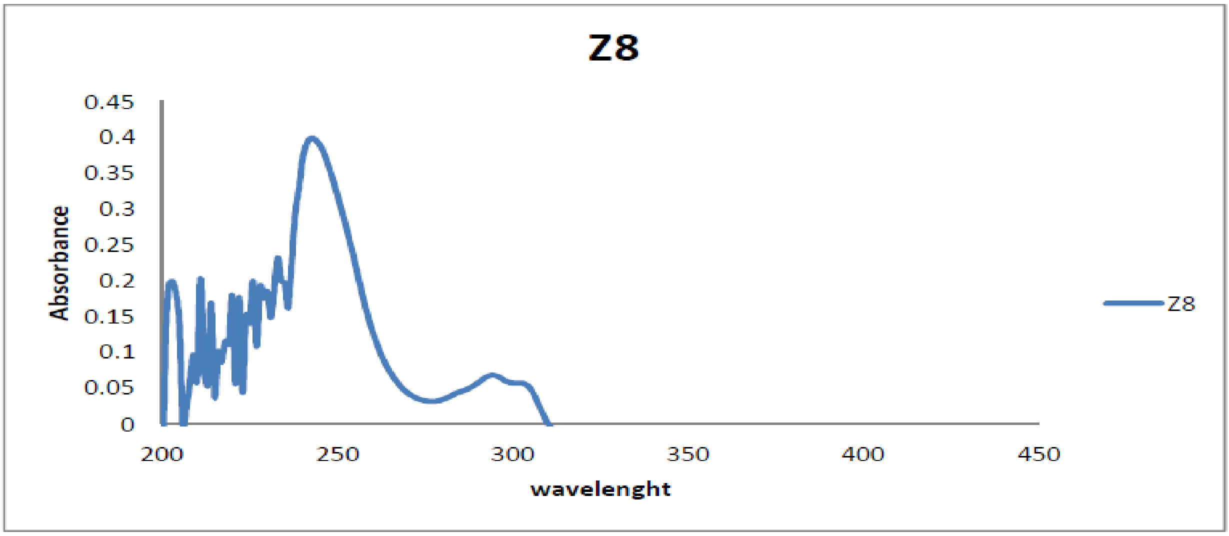

Appendix40: UV spectrum of Z_8

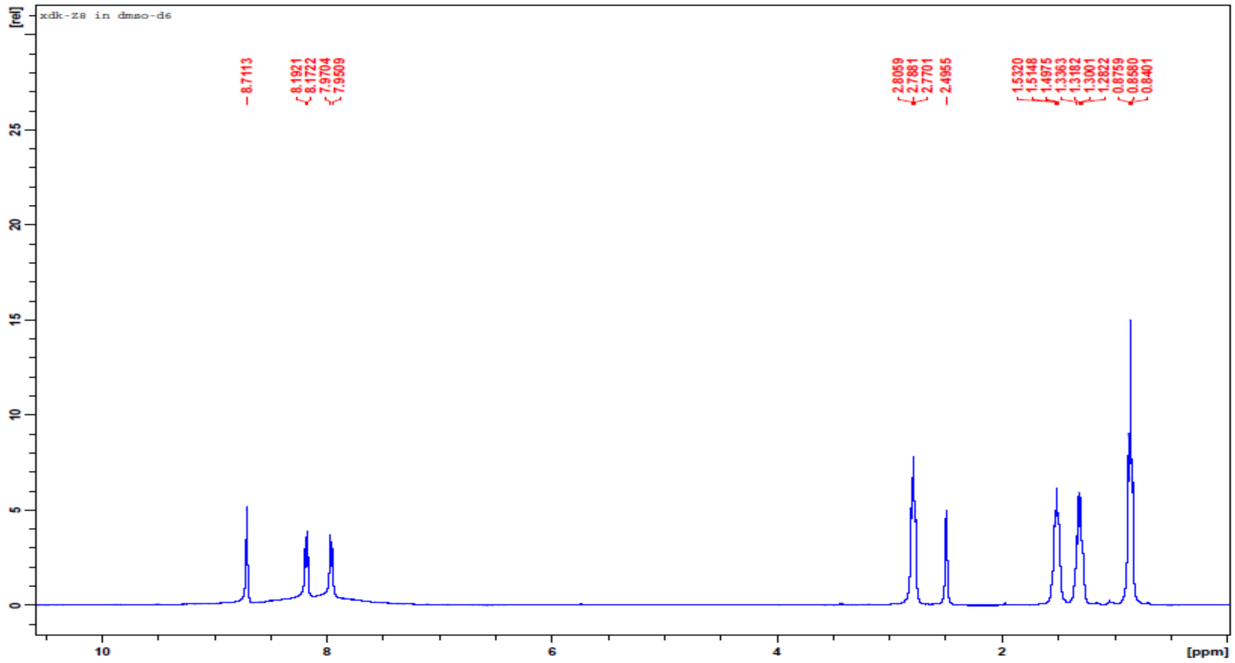

Appendix 41: ^{1}H NMR spectrum of Z_8

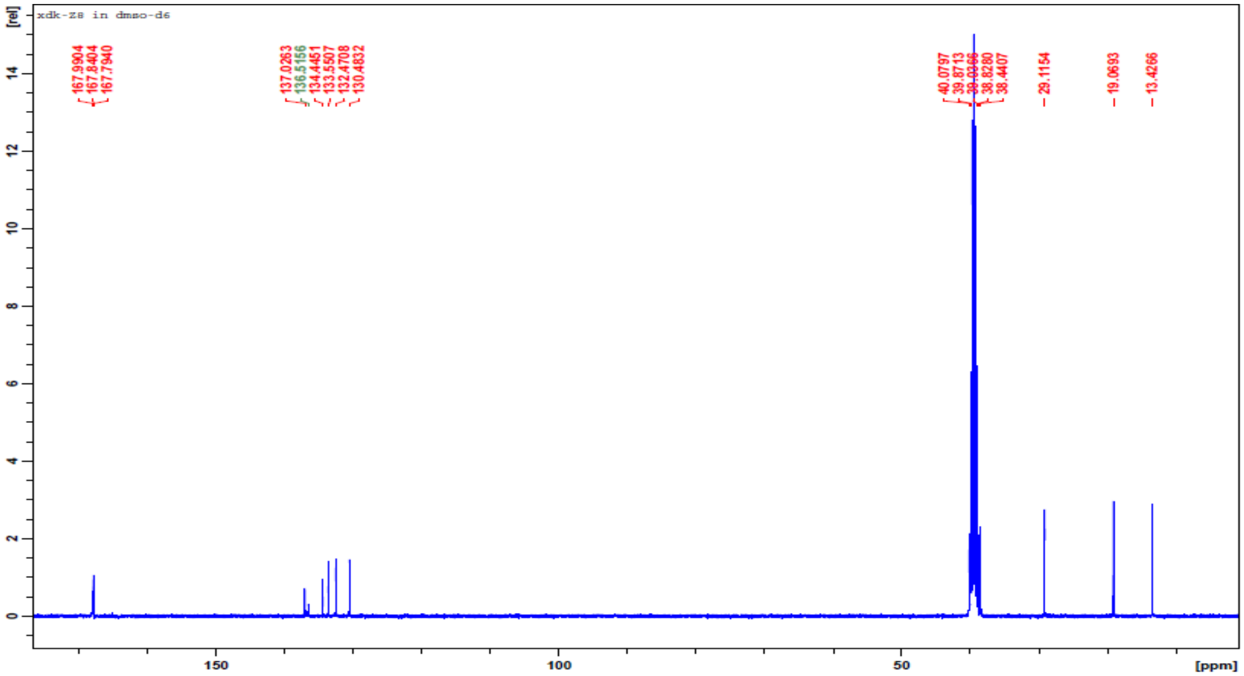

Appendix 42: ^{13}C NMR spectrum of Z_8

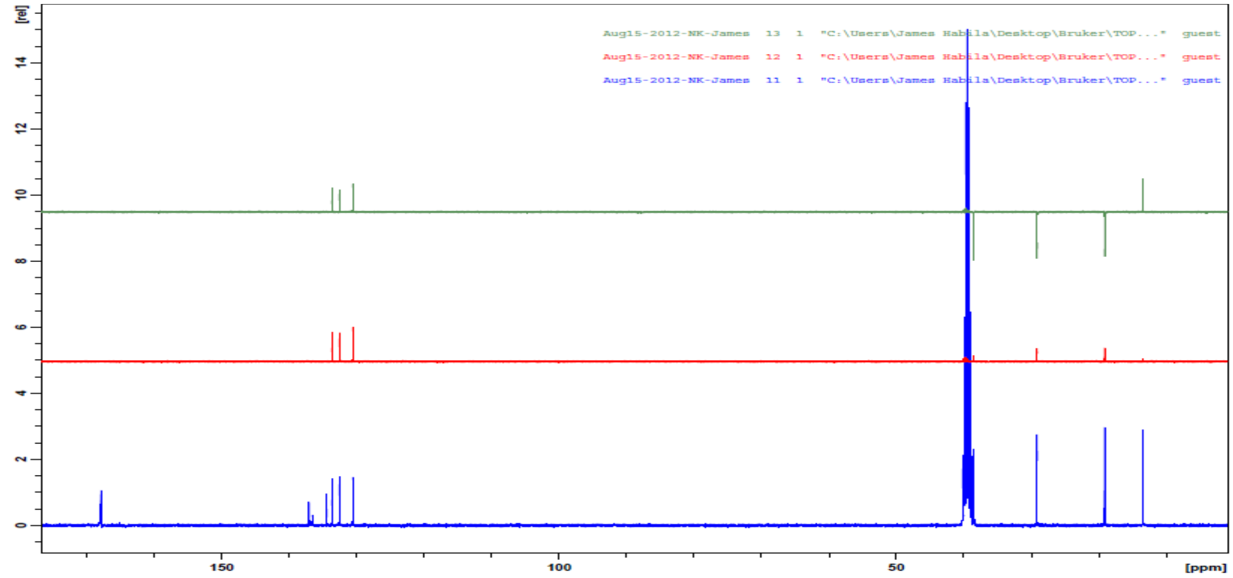

Appendix 43: DEPT spectrum of Z_8

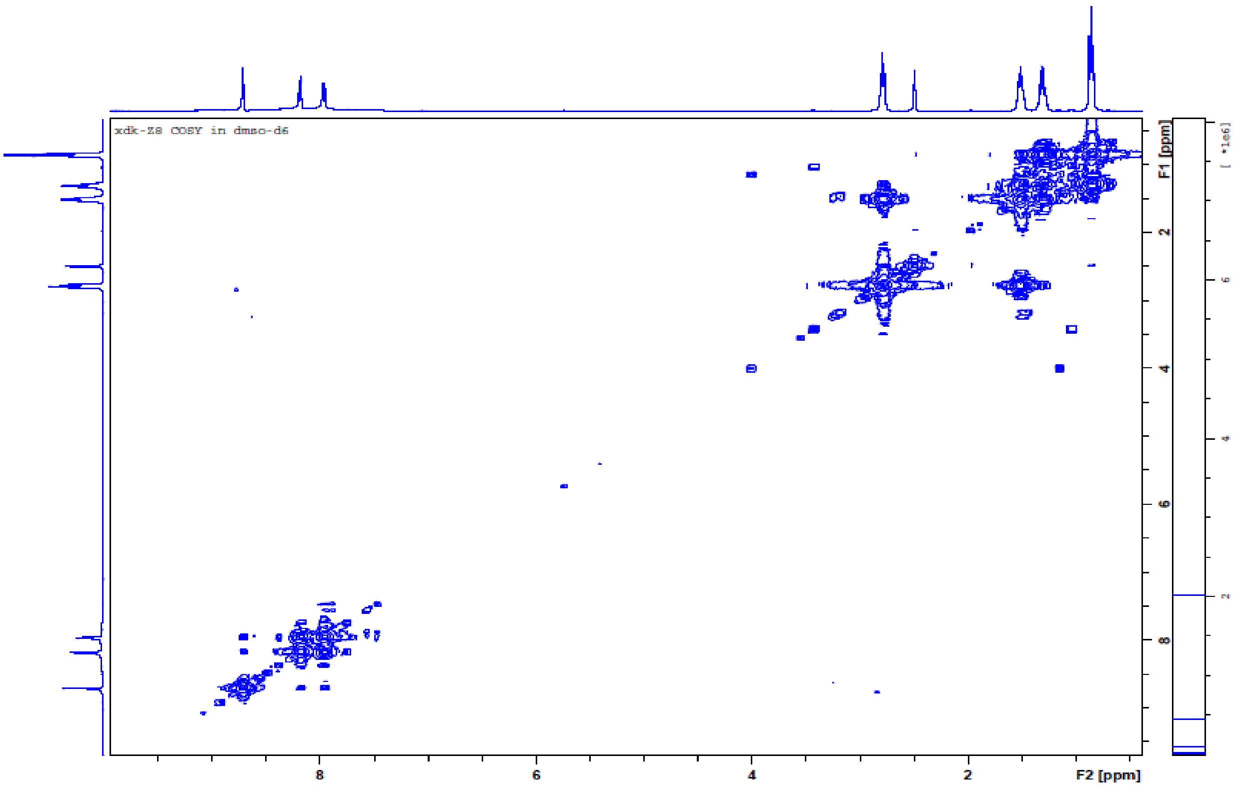

Appendix 43: COSY spectrum of Z_8

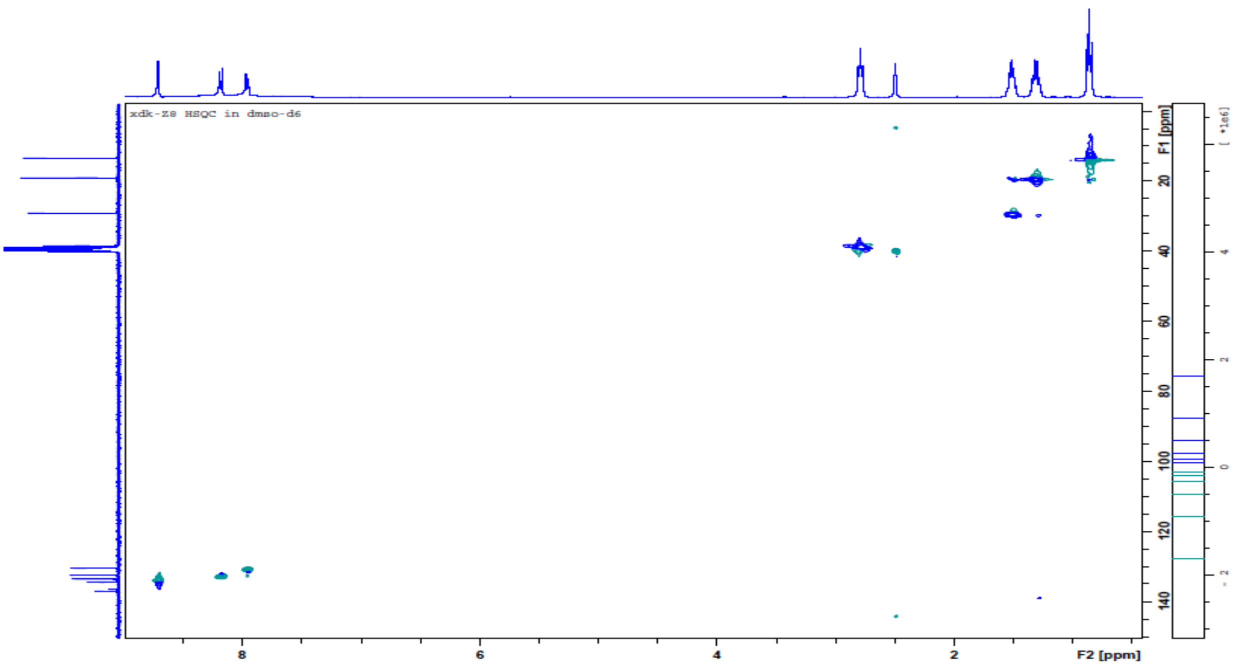

Appendix 44: HSQC spectrum of Z_8

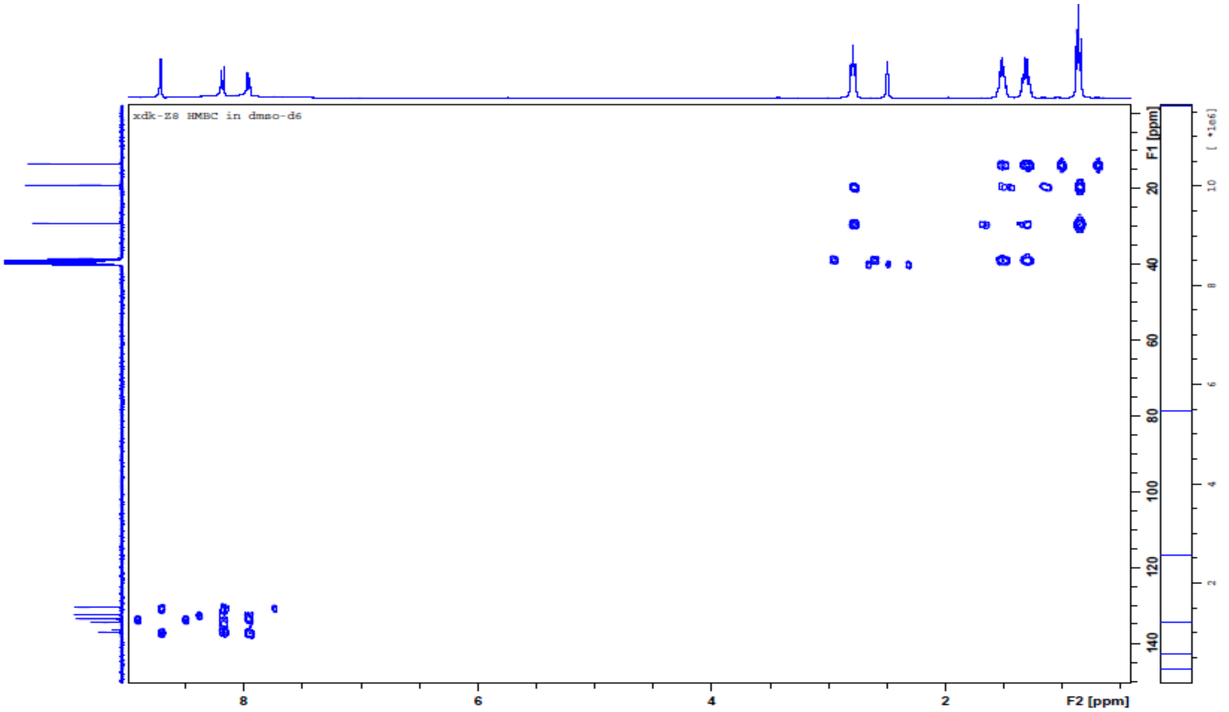

Appendix 45: HMBC spectrum of Z_8

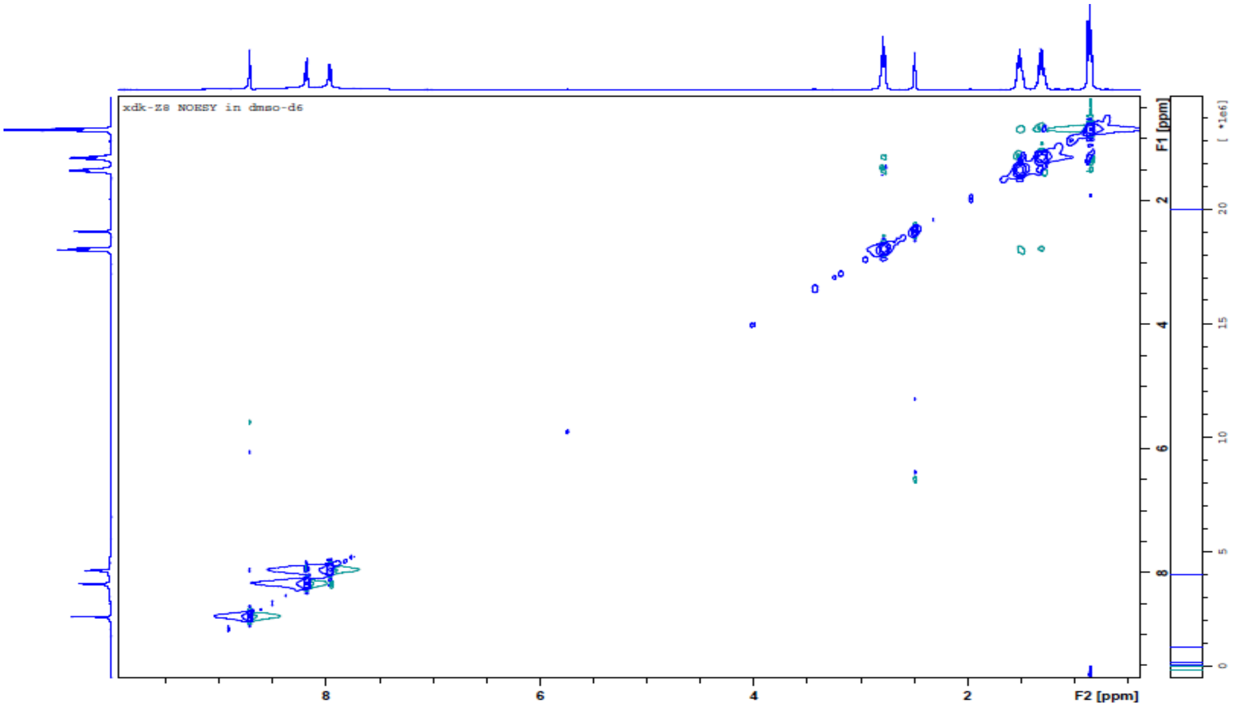

Appendix 46: NOESY spectrum of Z_8

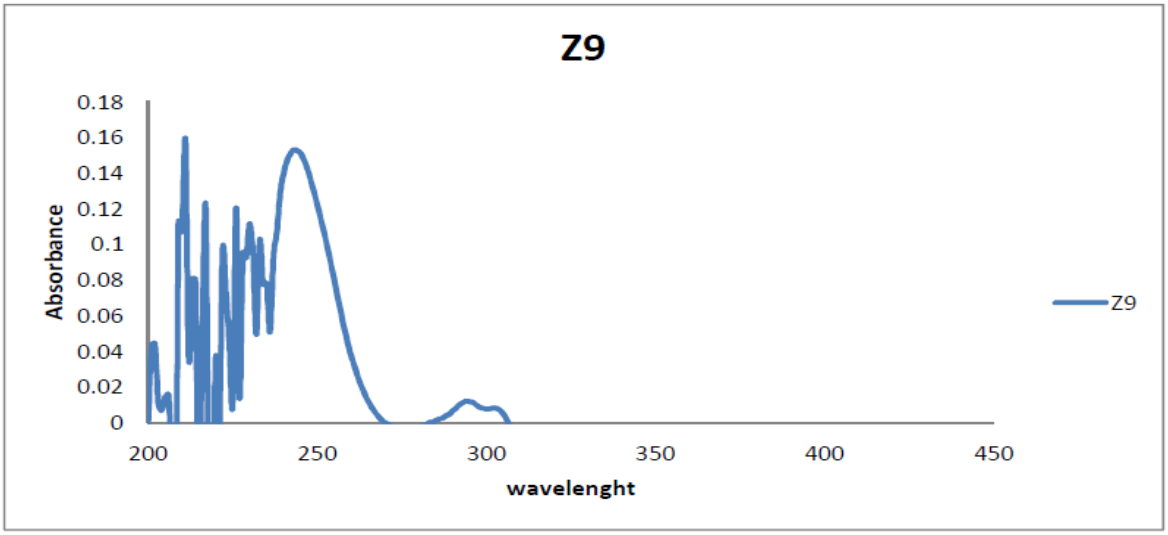

Appendix 47: UV spectrum of Z_9

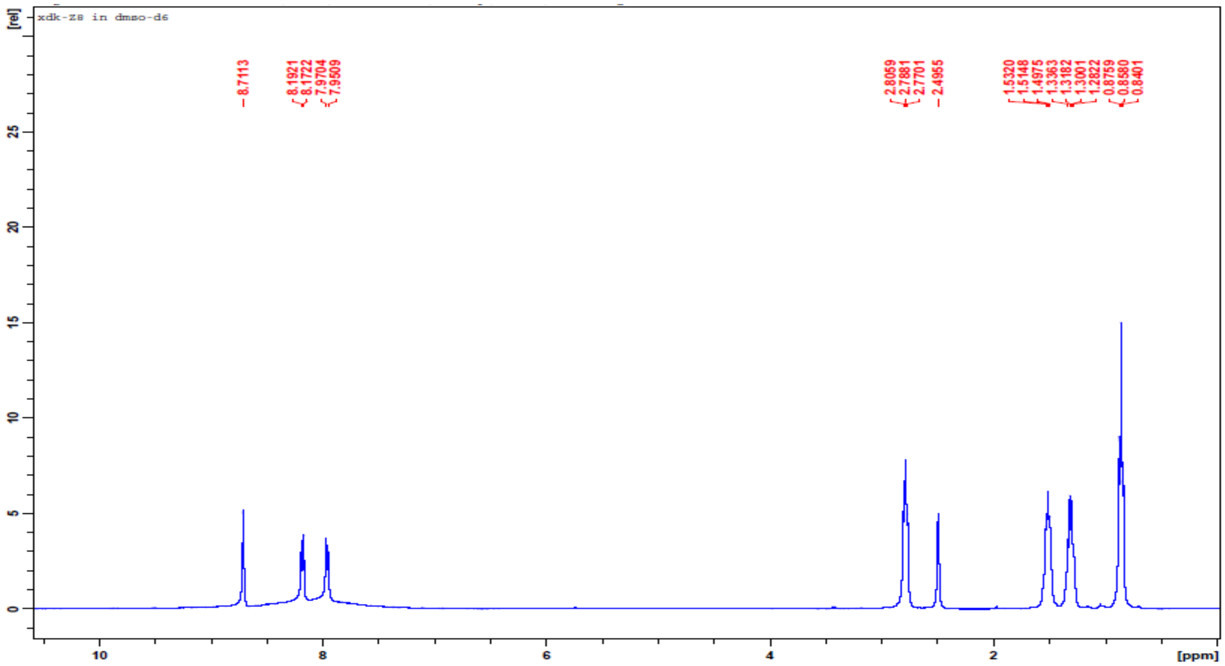

Appendix 48; ^{1}H NMR spectrum of Z_9

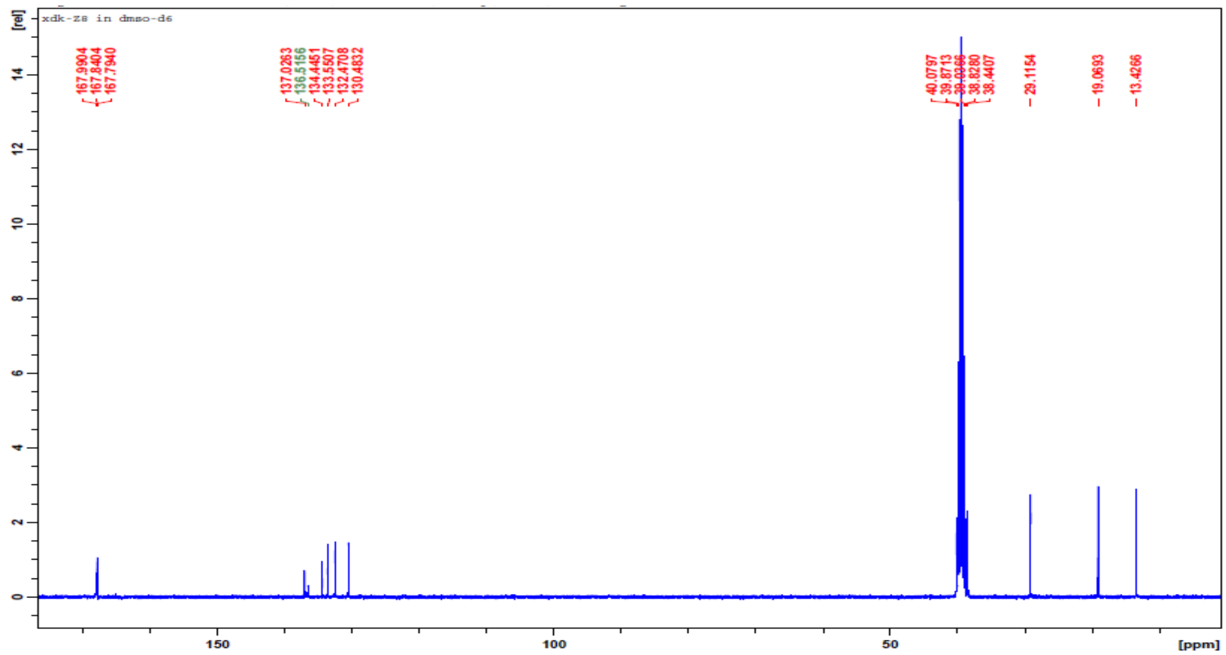

Appendix 49: ^{13}C NMR spectrum of Z_9

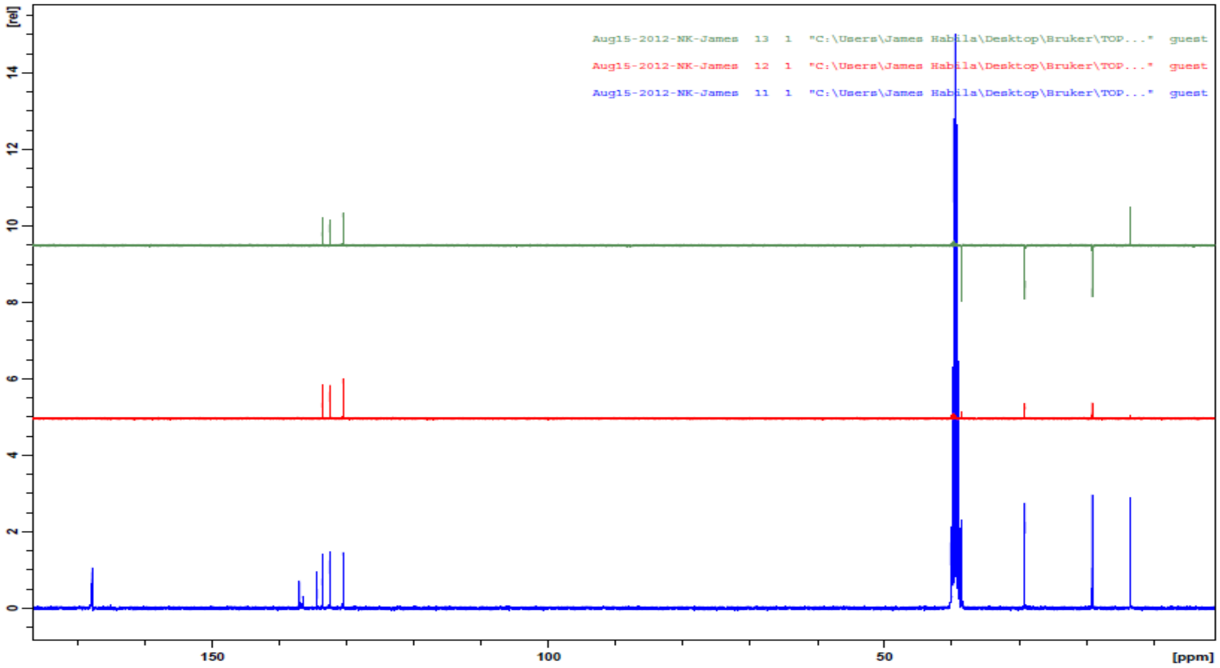

Appendix 50: DEPT spectrum of Z_9

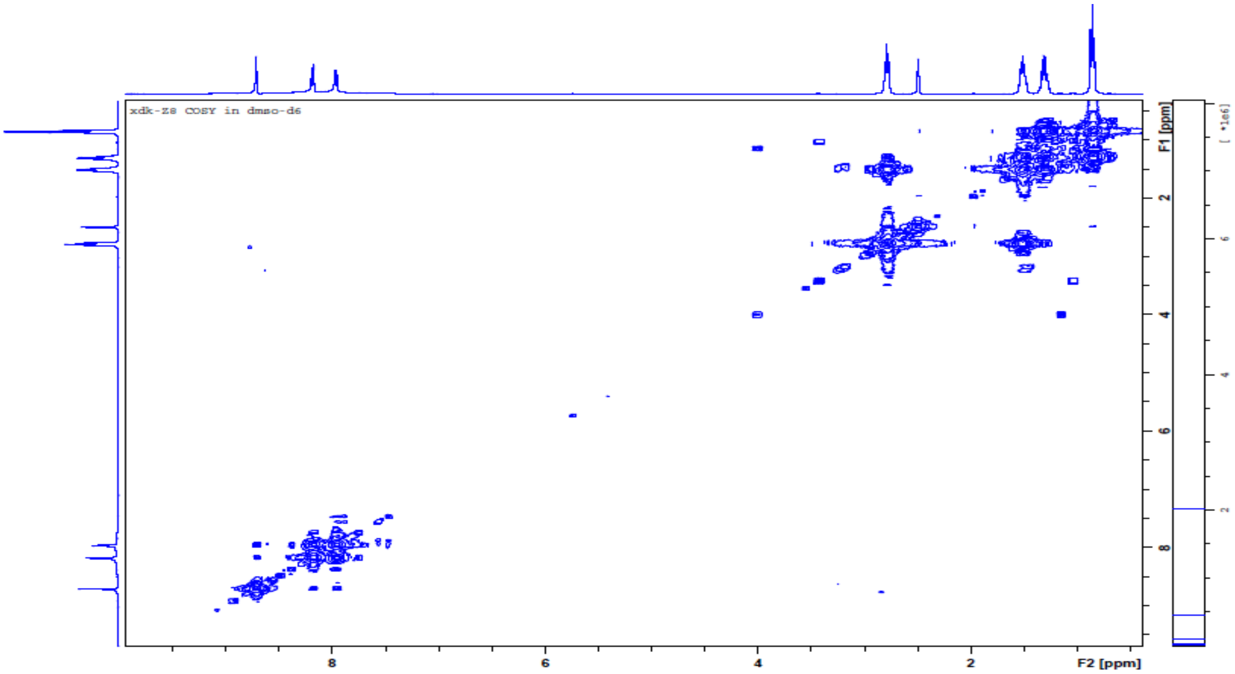

Appendix 51; COSY spectrum of Z_9

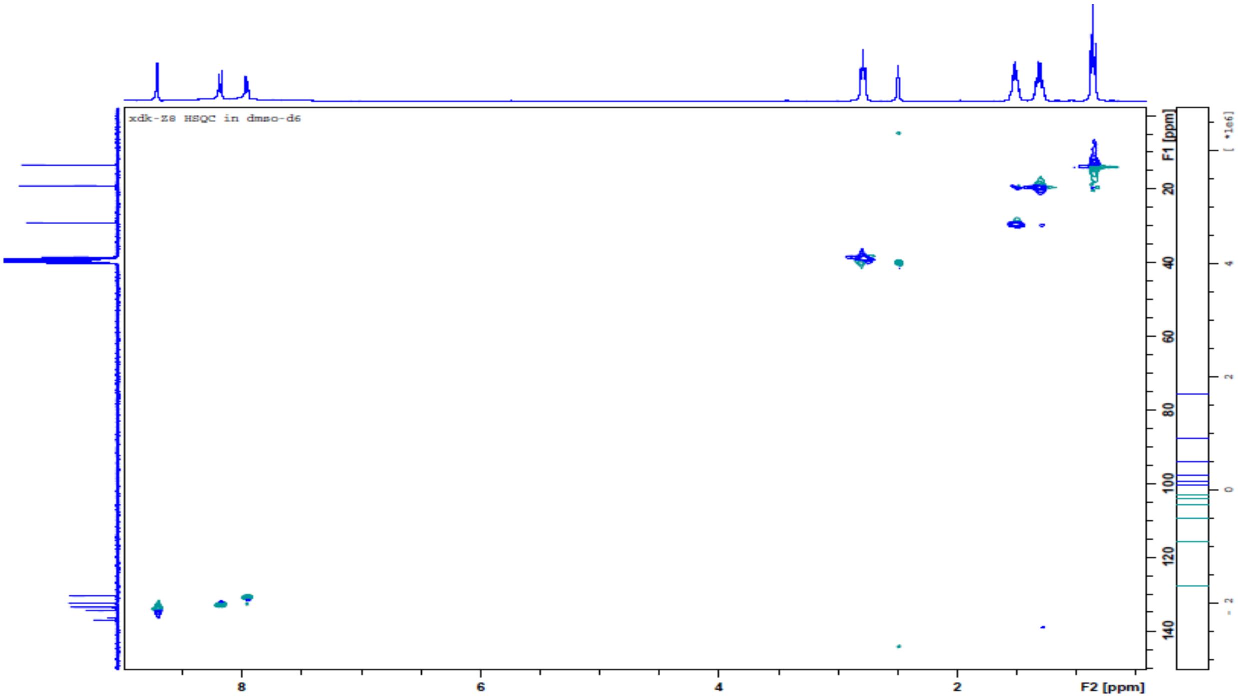

Appendix 52: HSQC spectrum of Z_9

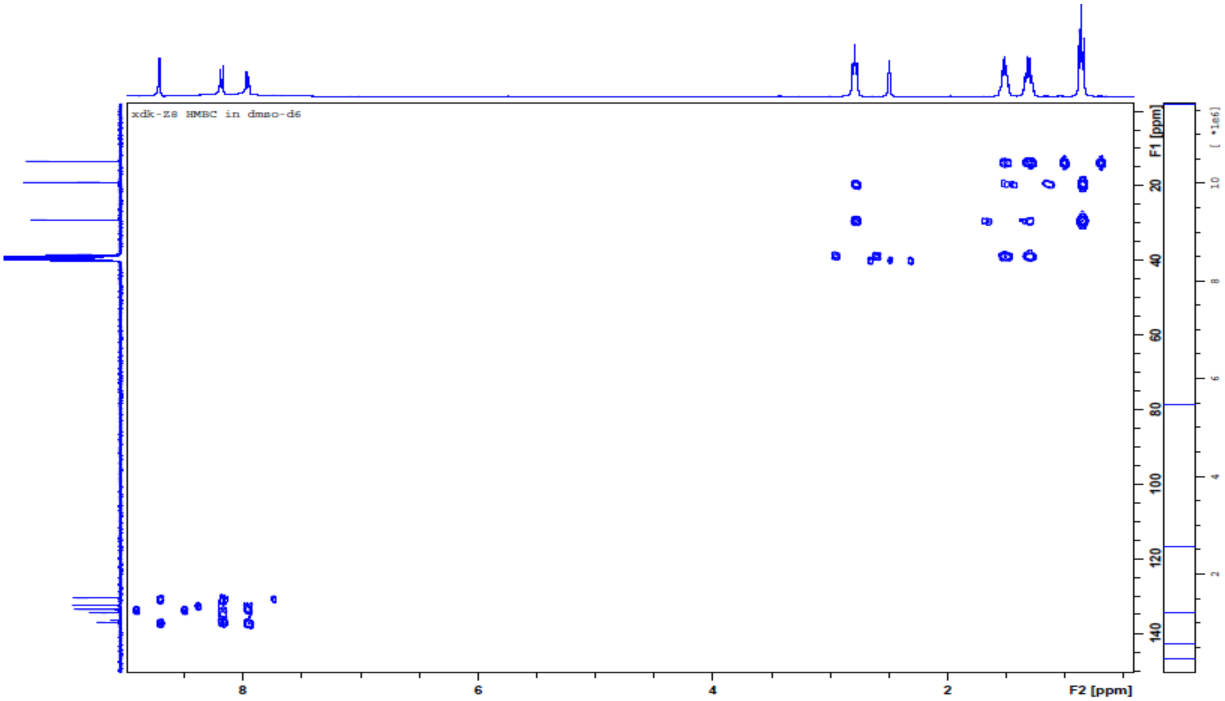

Appendix 53: HMBC spectrum of Z_9

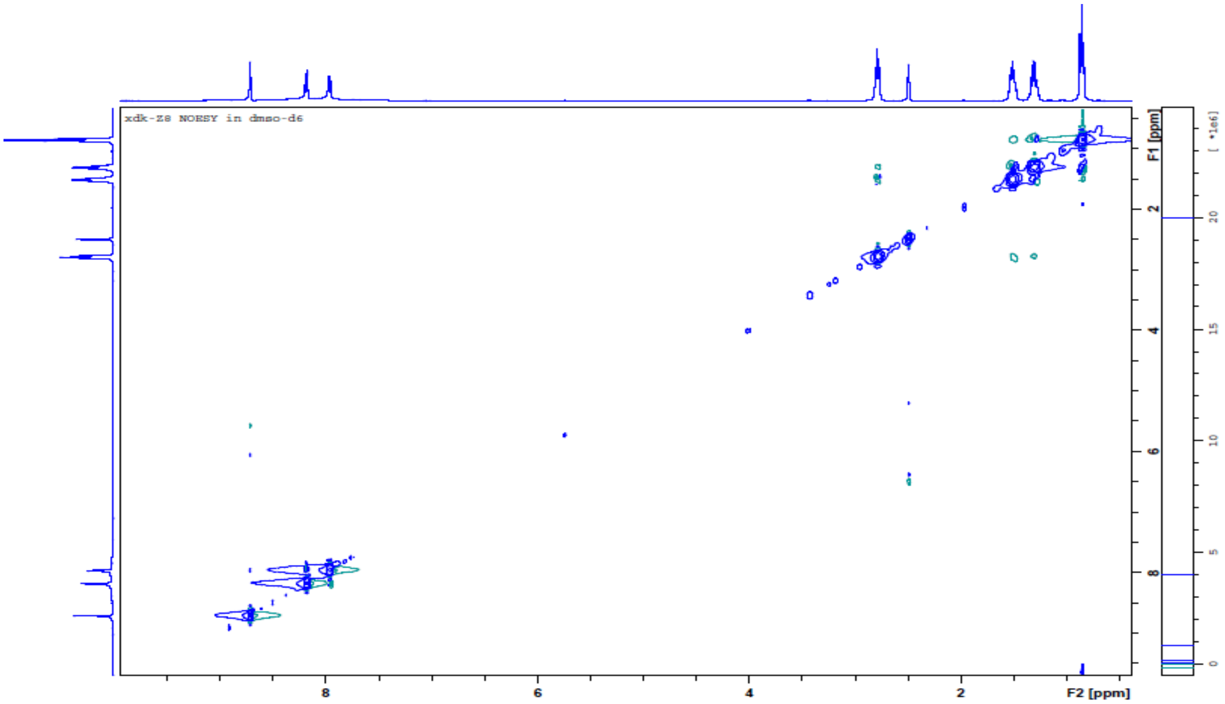

Appendix 54: NOESY spectrum of Z_9

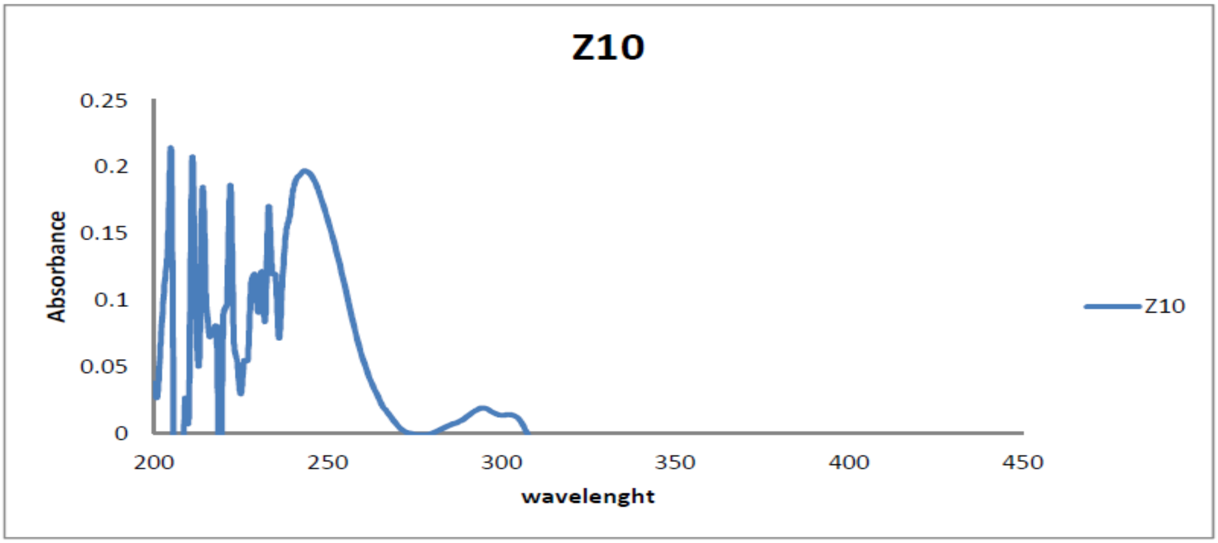

Appendix 55: UV spectrum of Z_{10}

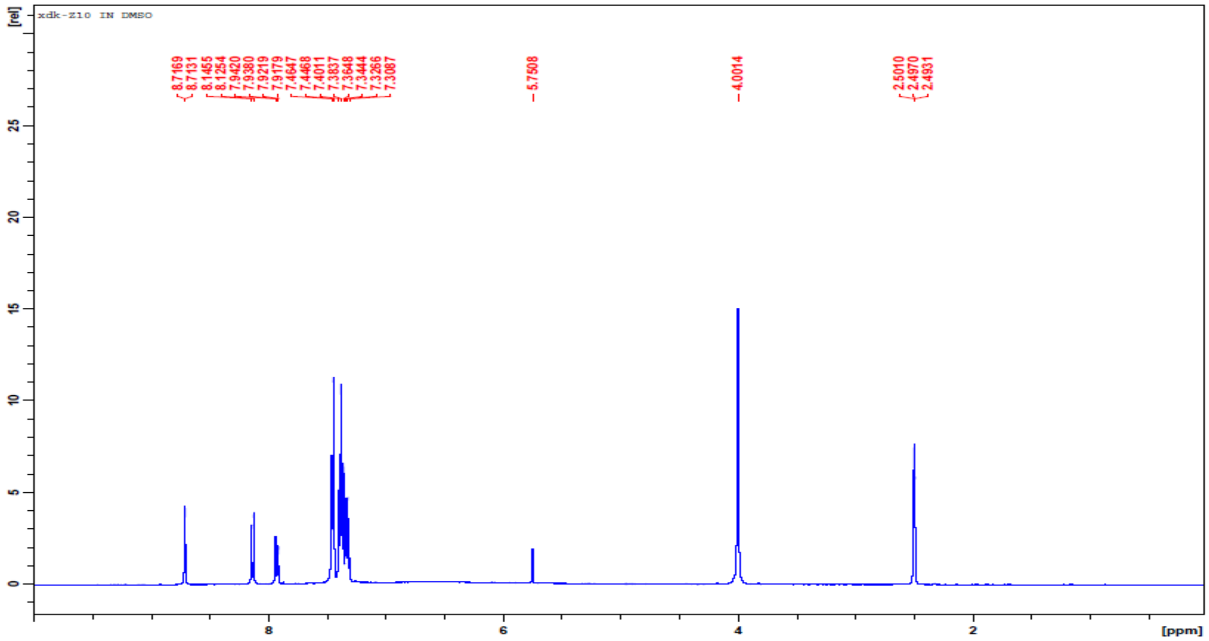

Appendix 56: ^{1}H NMR spectrum of Z_{10}

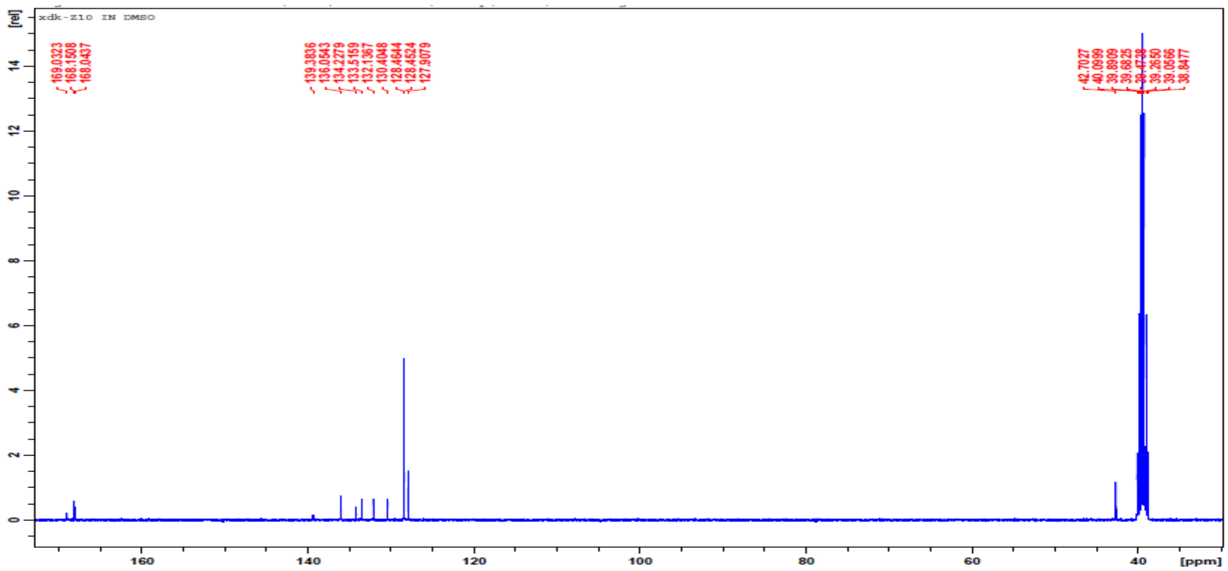

Appendix 57: ^{13}C NMR spectrum of Z_{10}

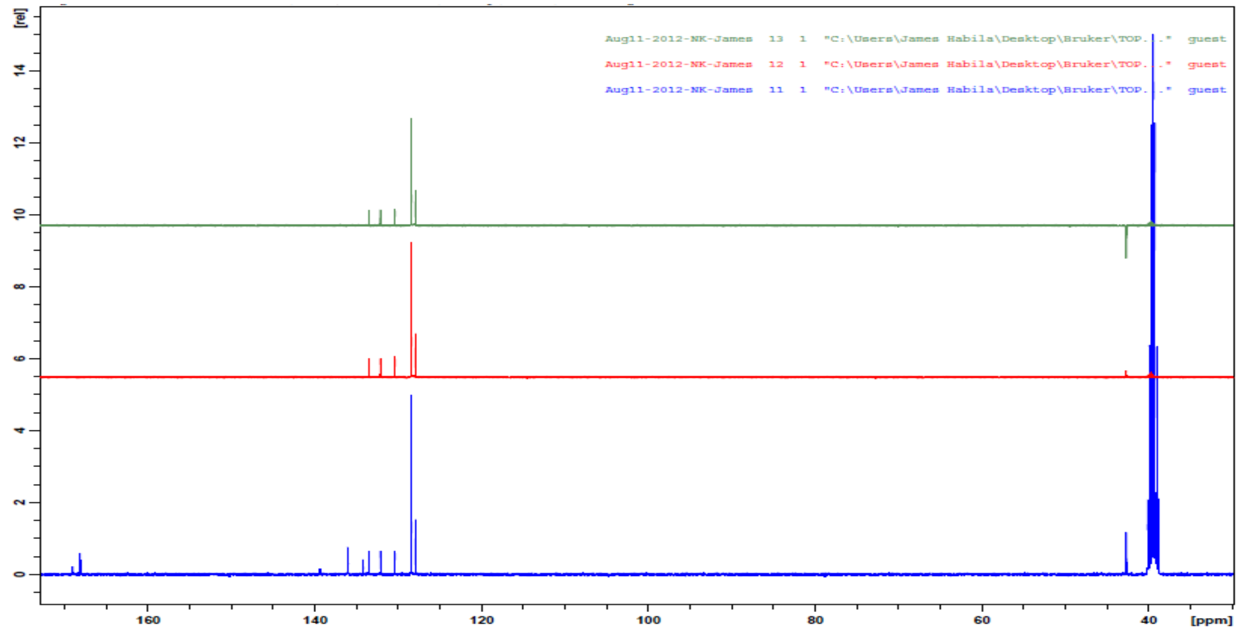

Appendix 58: DEPT spectrum of Z_{10}

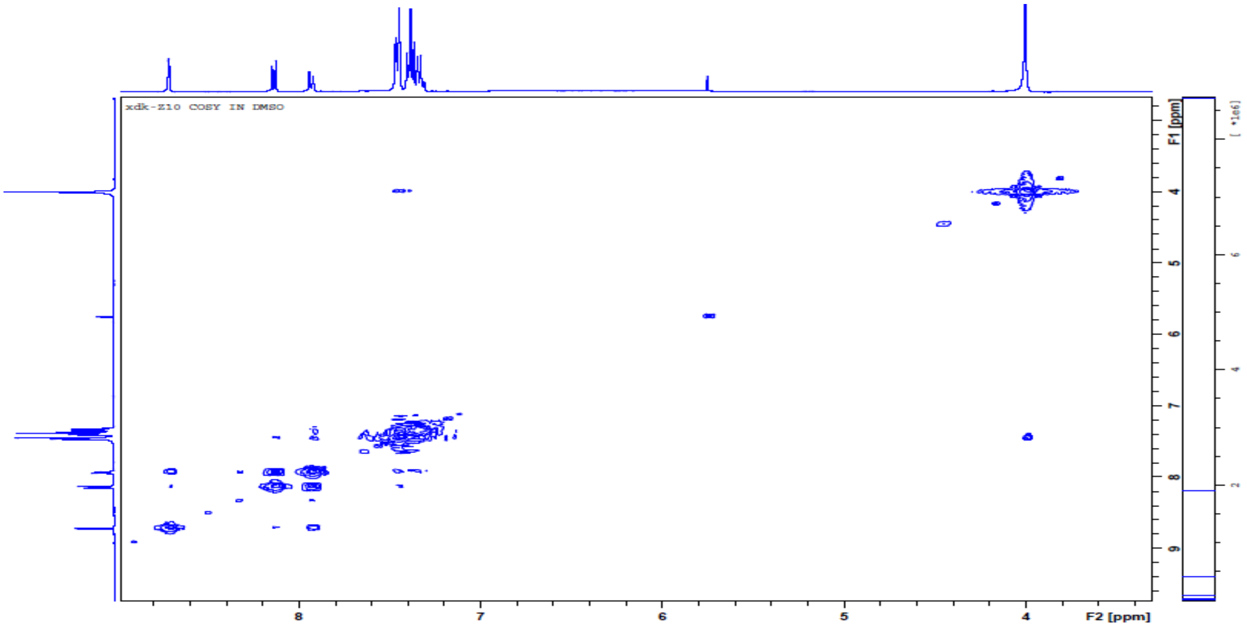

Appendix 59: COSY spectrum of Z_{10}

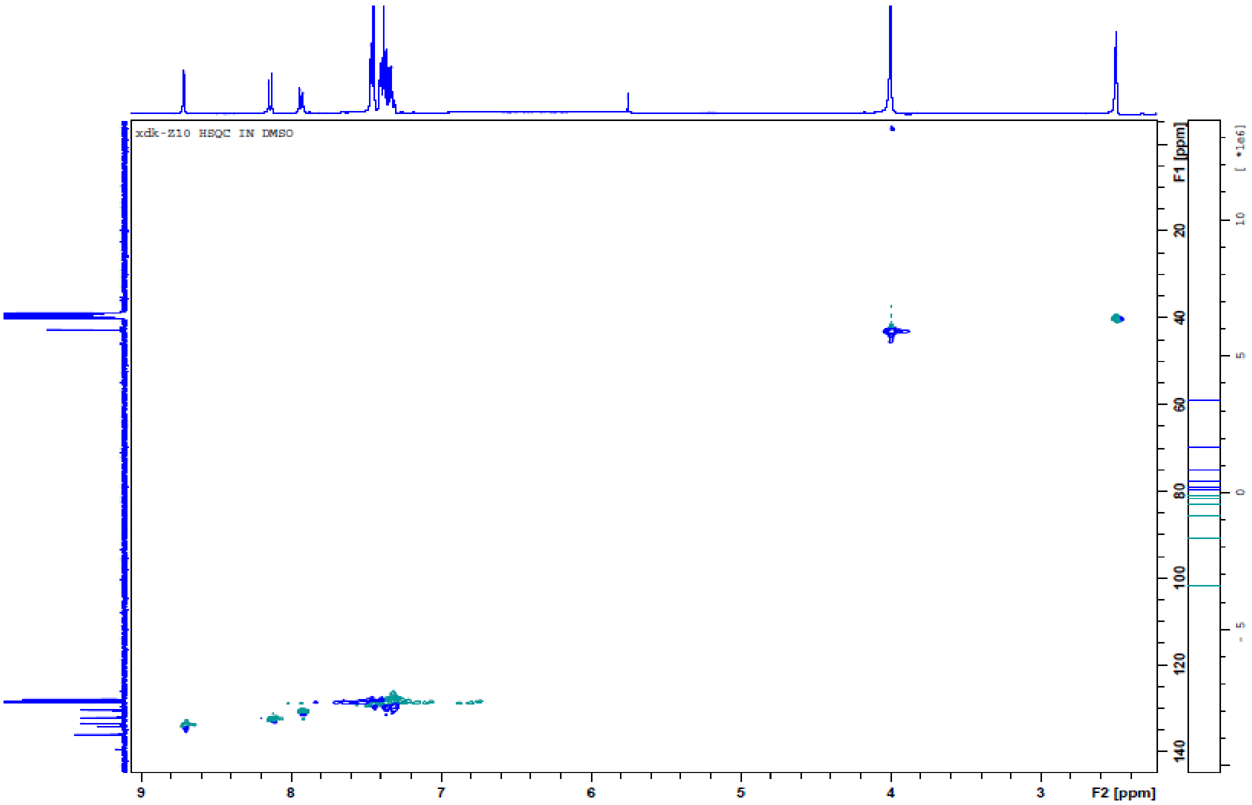

Appendix 60: HSQC spectrum of Z_{10}

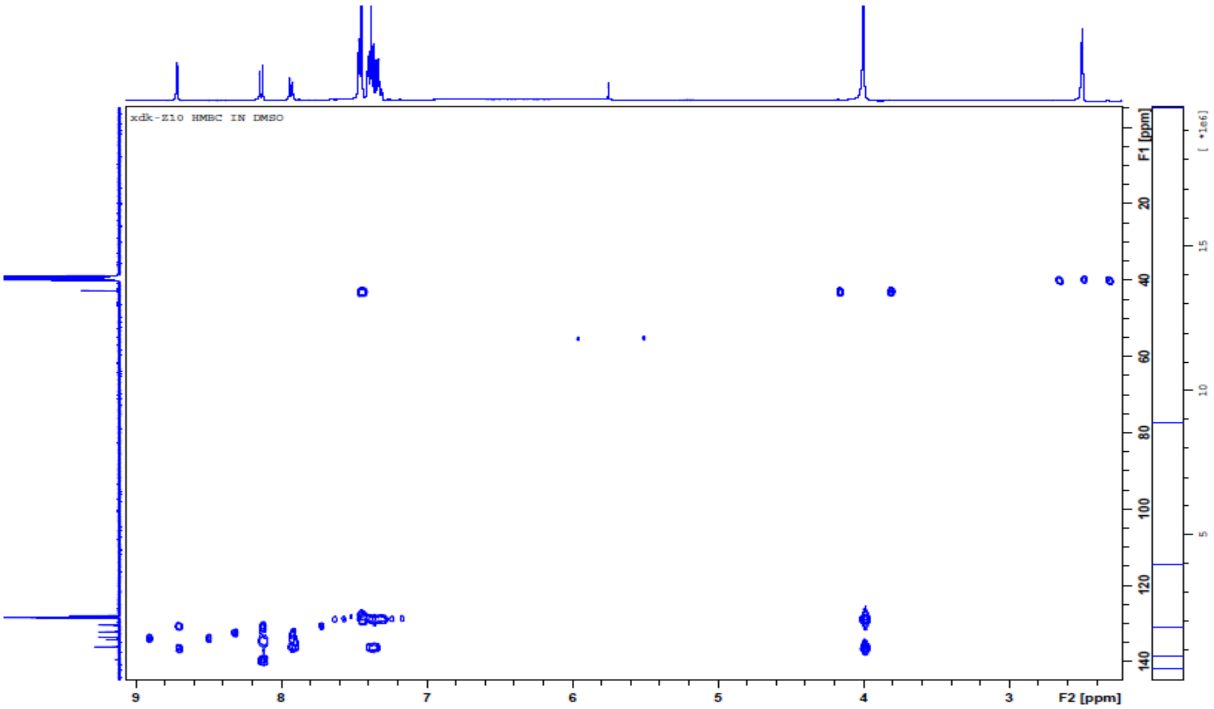

Appendix 61: HMBC spectrum of Z_{10}

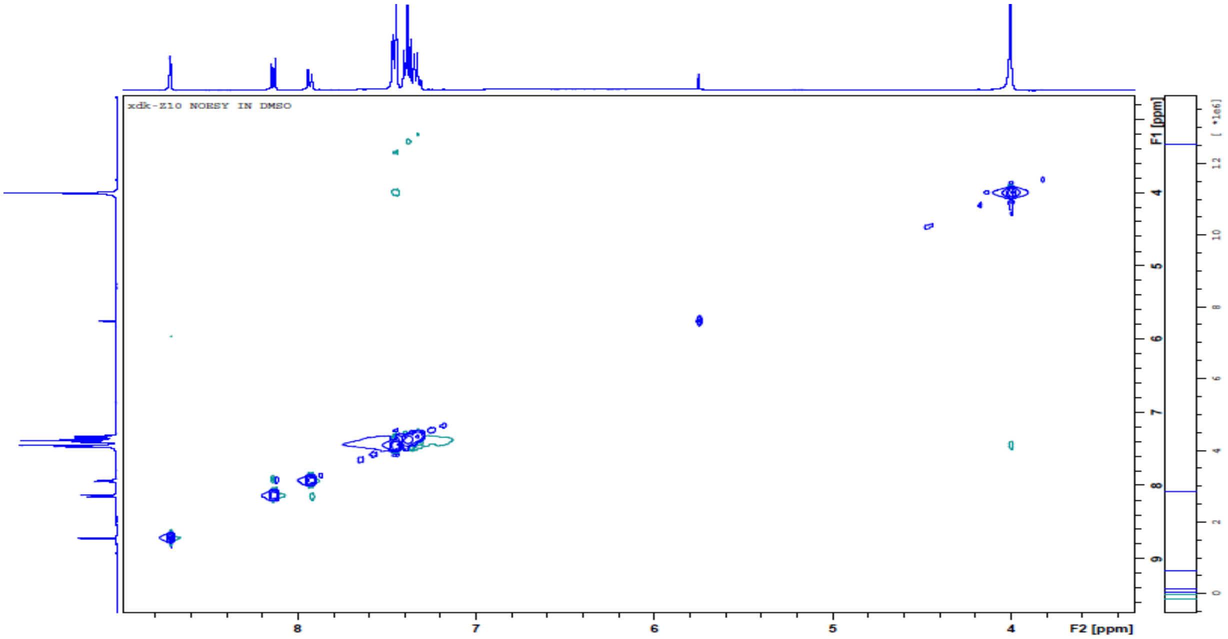

Appendix 62: NOESY spectrum of Z_{10}

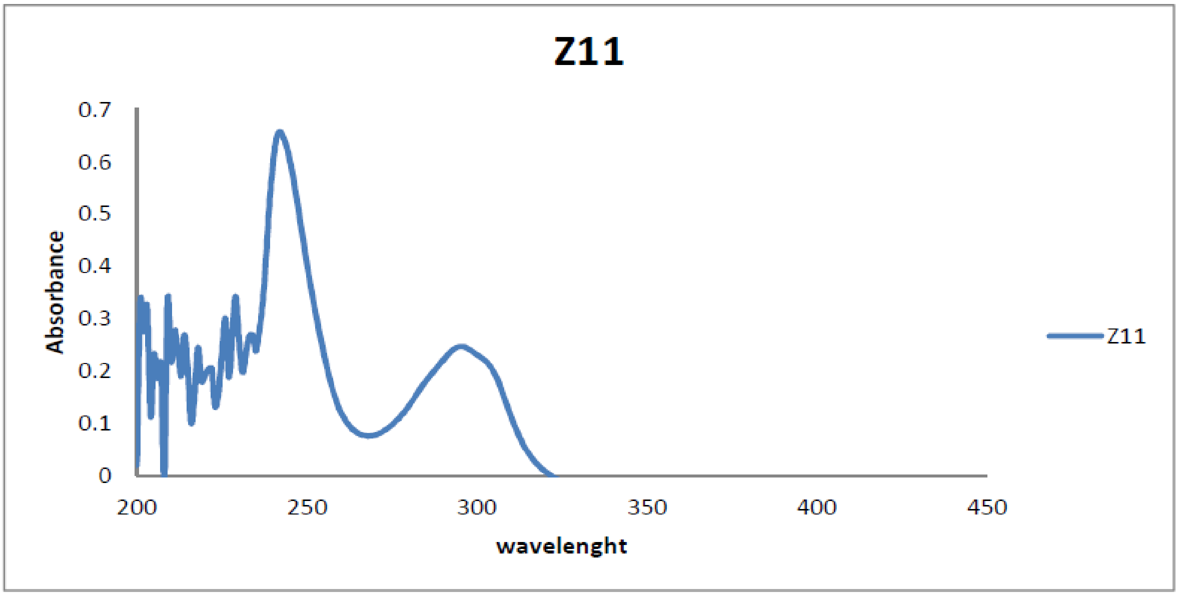

Appendix 63: UV spectrum of Z_{11}

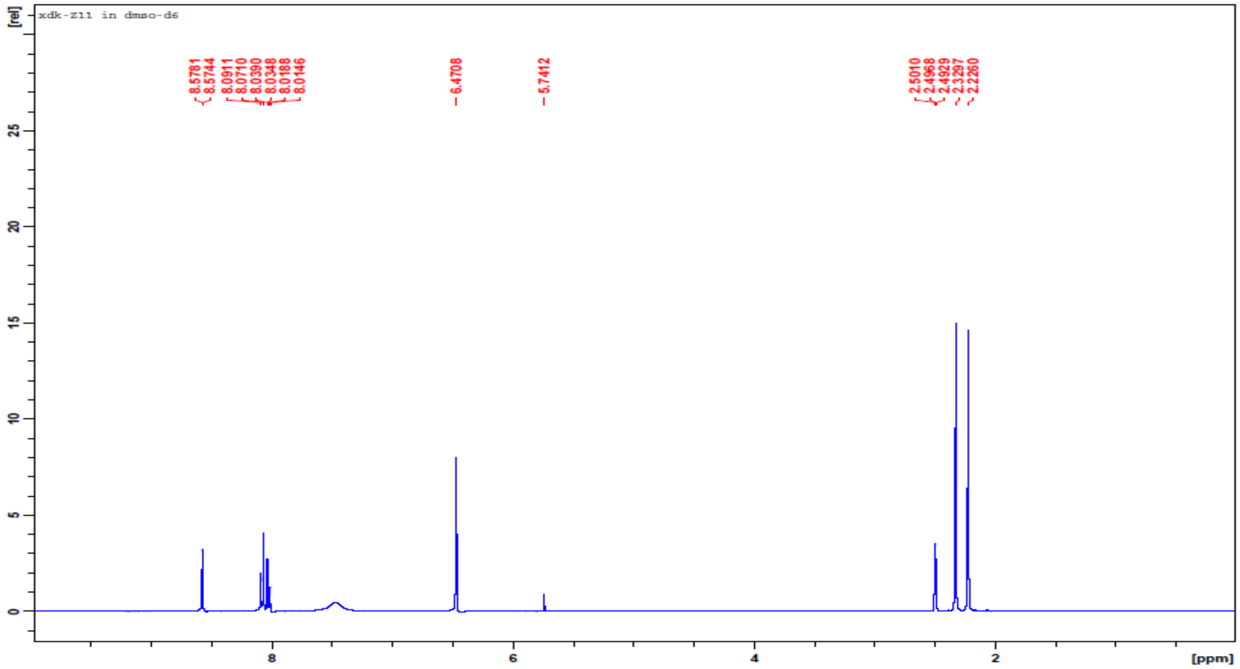

Appendix 64; ^{1}HNMR spectrum of Z_{11}

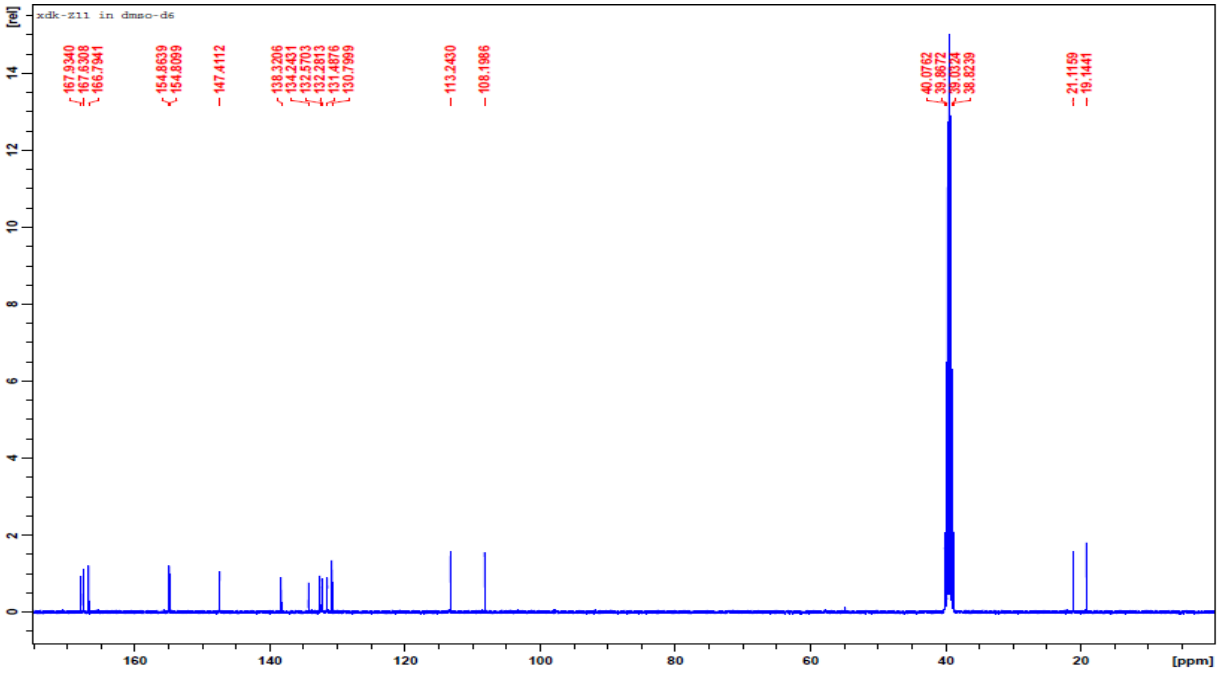

Appendix 65: ^{13}C NMR spectrum of Z_{11}

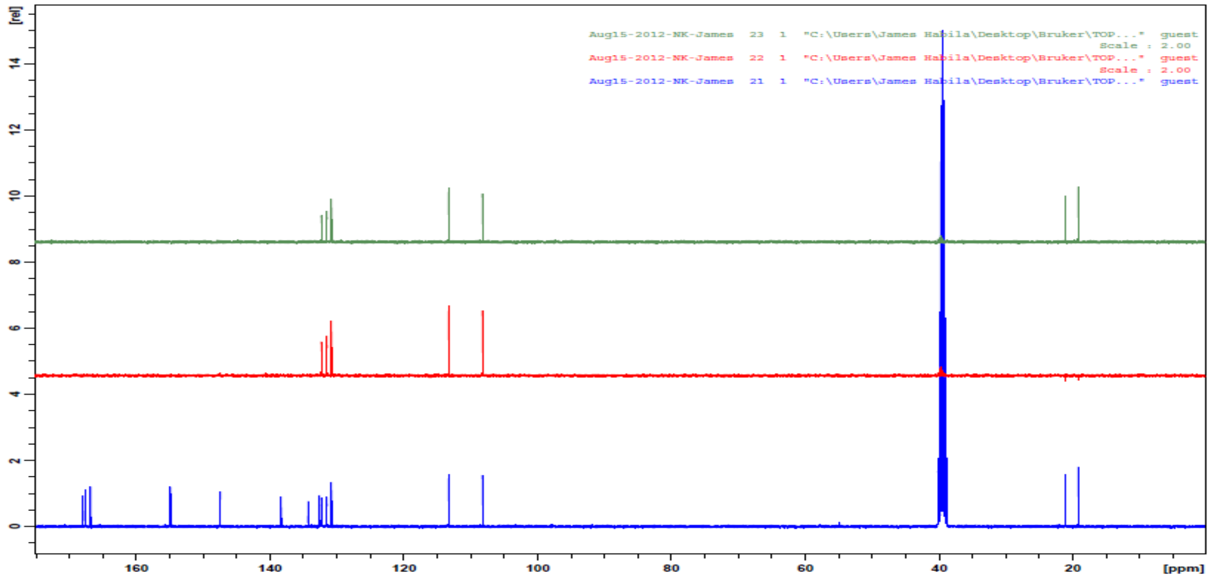

Appendix 66: DEPT spectrum of Z_{11}

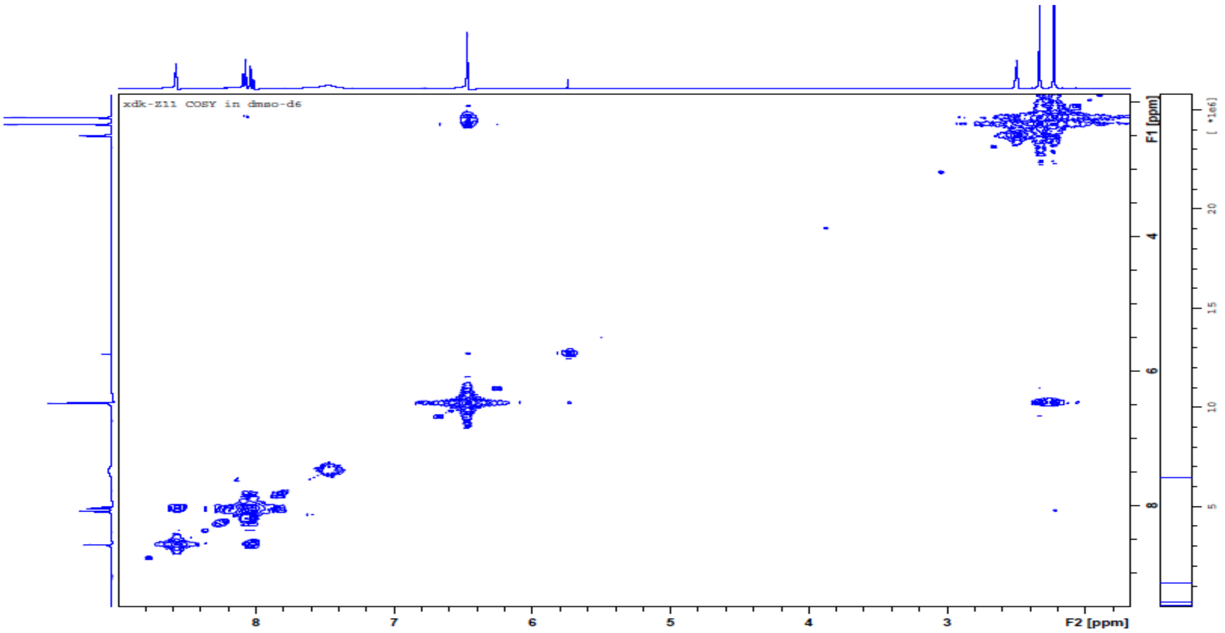

Appendix 67: COSY spectrum of Z_{11}

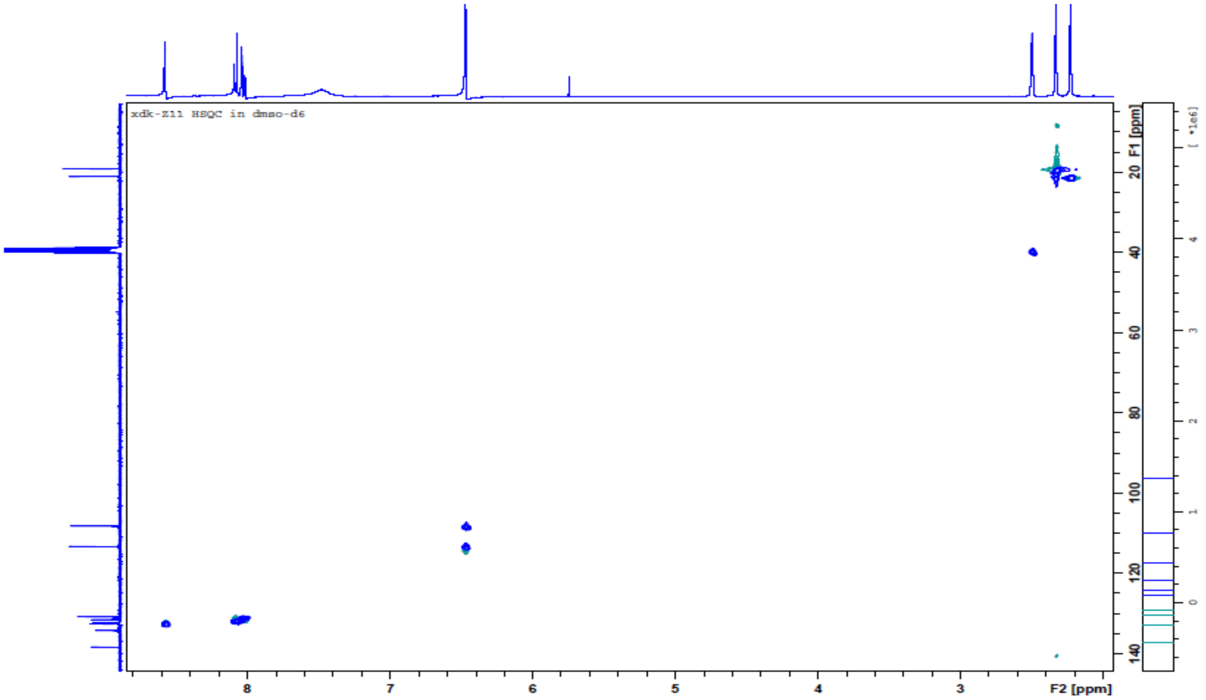

Appendix 68: HSQC spectrum of Z_{11}

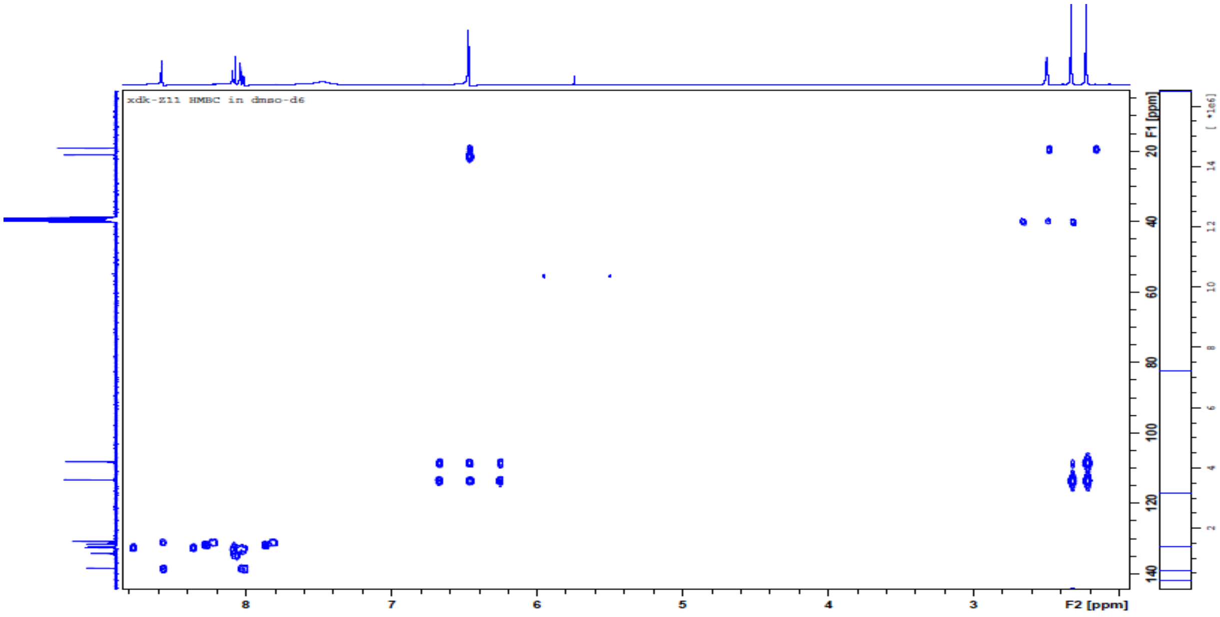

Appendix 69: HMBC spectrum of Z_{11}

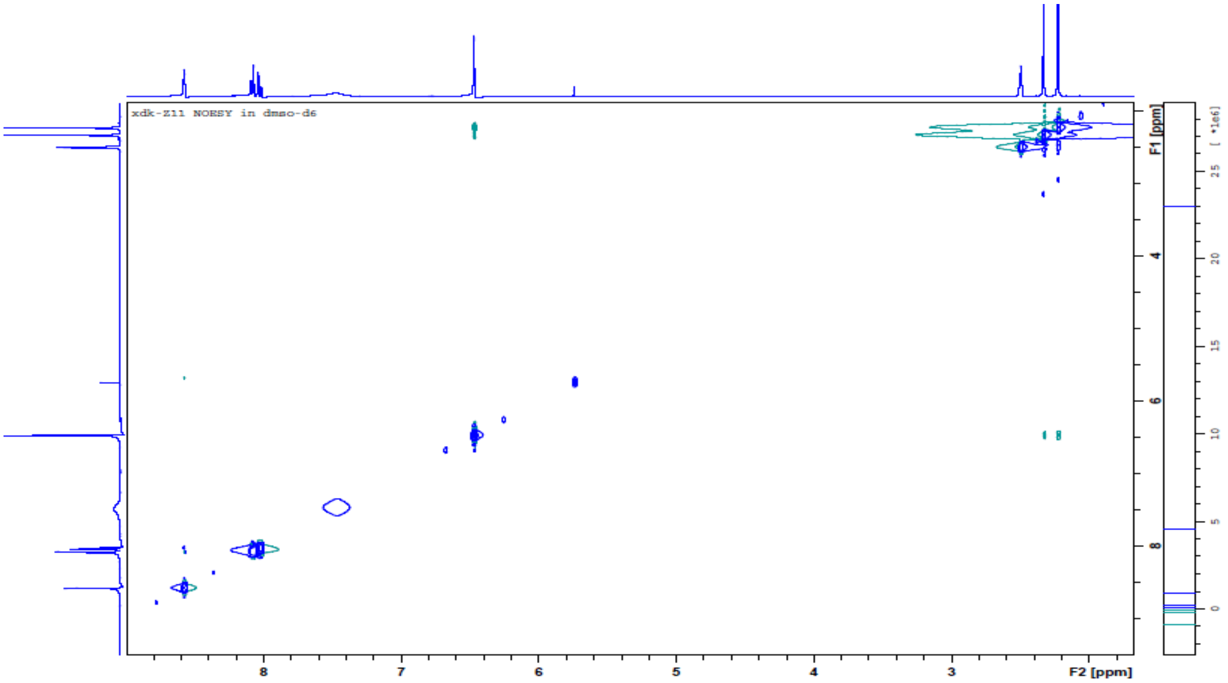

Appendix 70: NOESY spectrum of Z_{11}

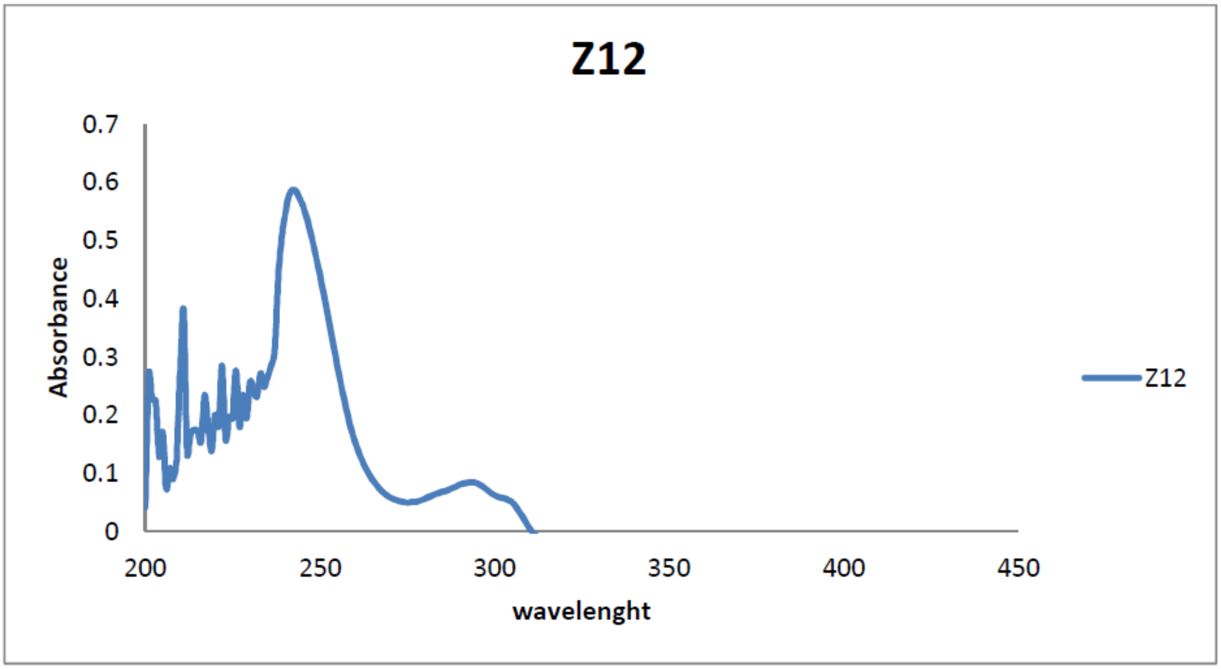

Appendix 71: UV spectrum of Z_{12}

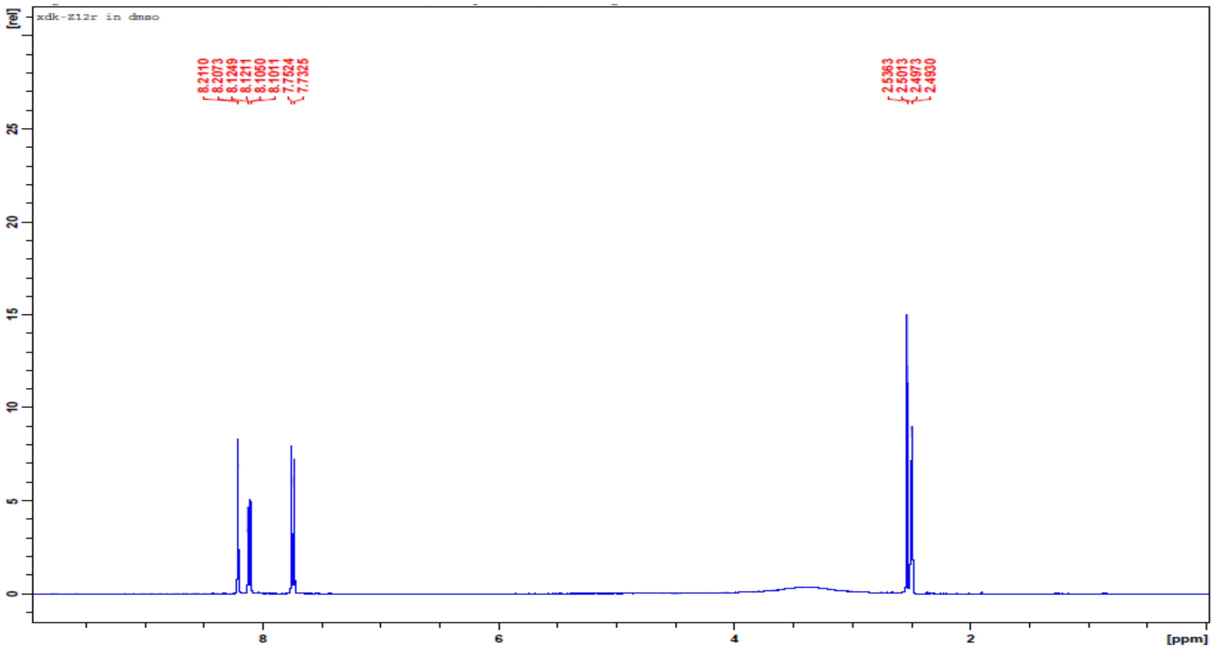

Appendix 72; ^{1}HNMR spectrum of Z_{12}

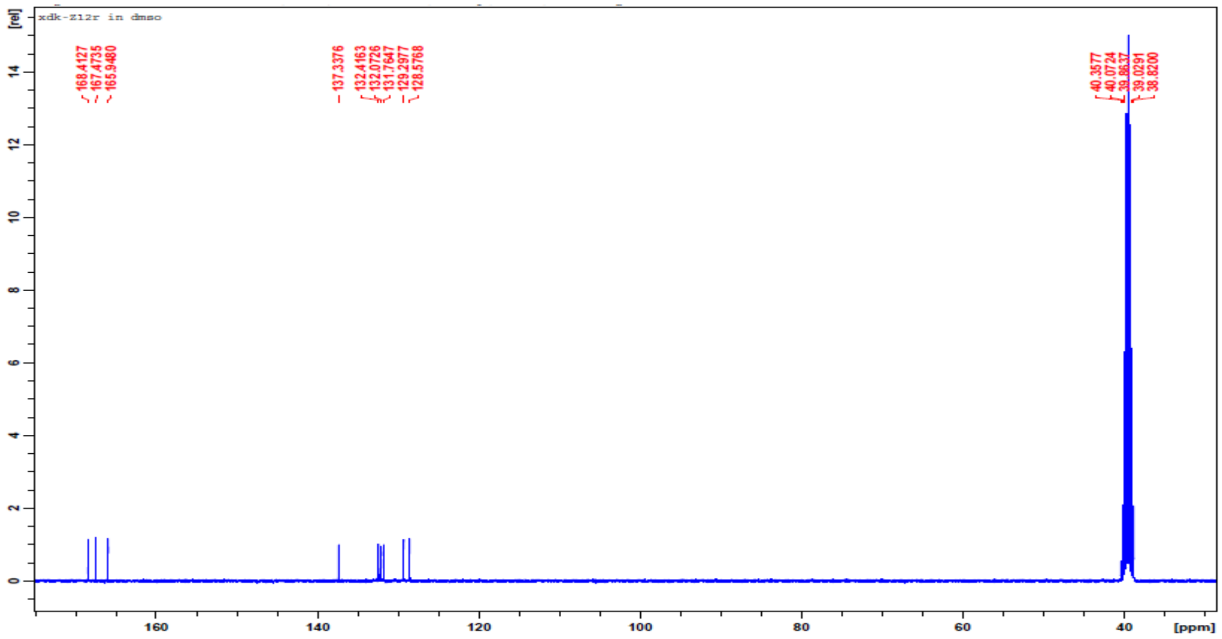

Appendix 73: ^{13}C NMR spectrum of Z_{12}

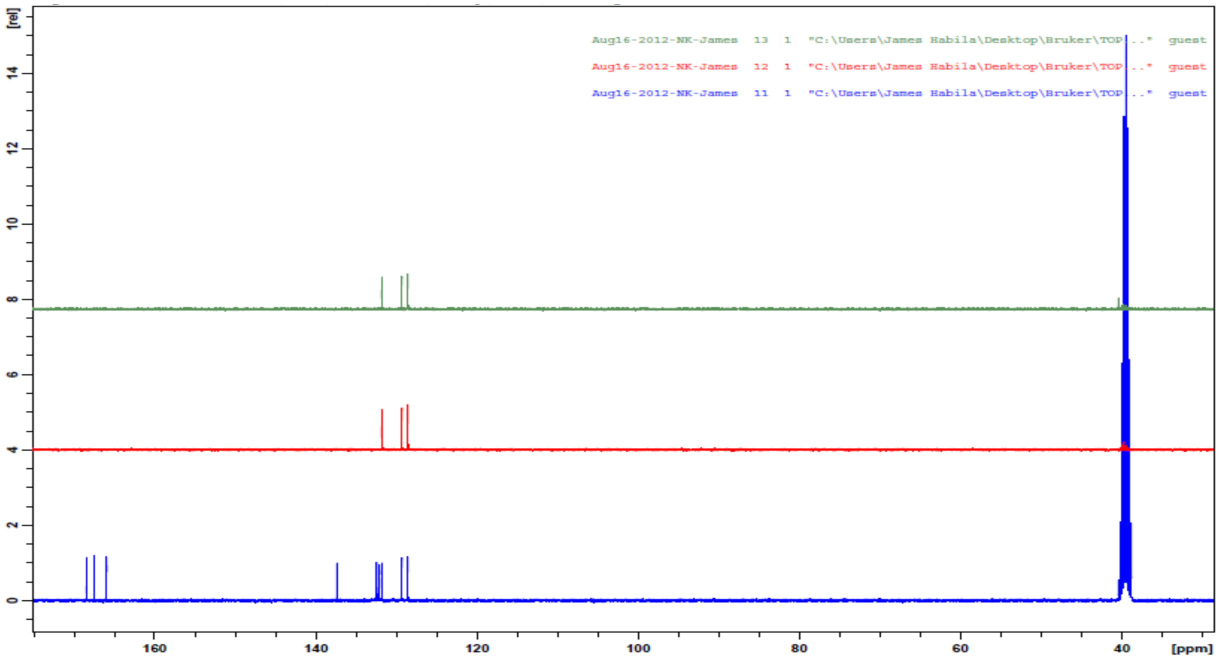

Appendix 74: DEPT spectrum of Z_{12}

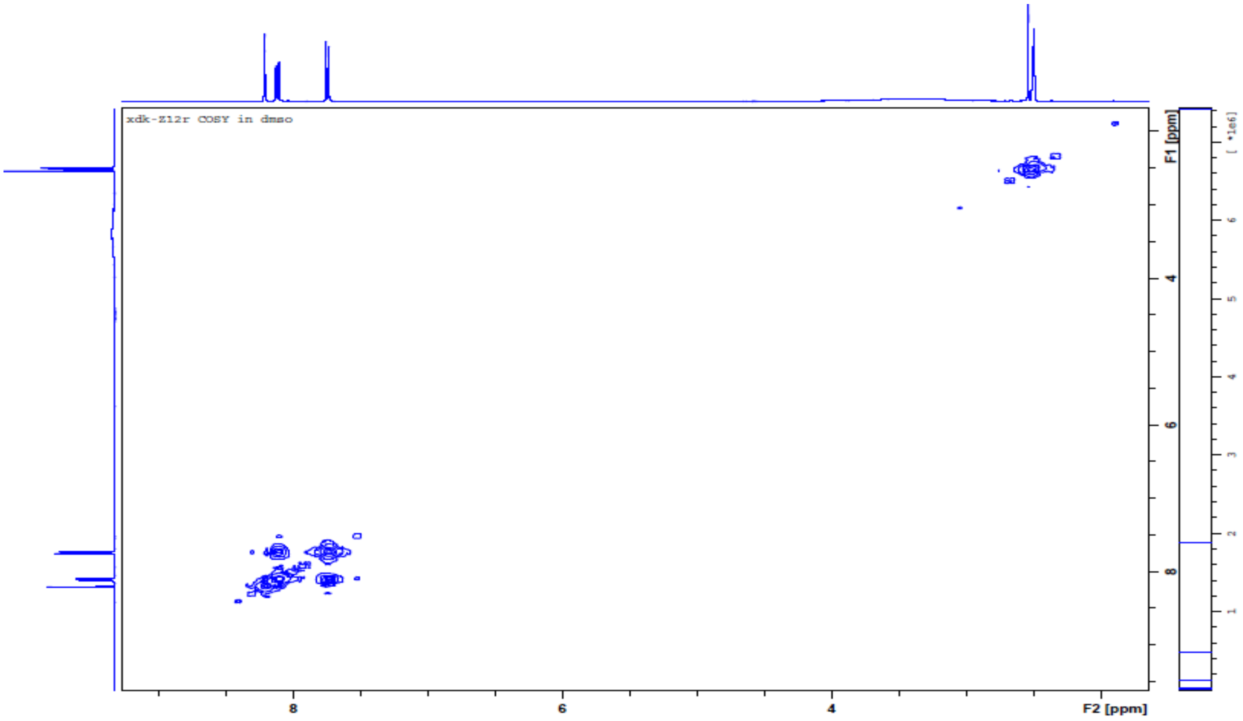

Appendix 75: COSY spectrum of Z_{12}

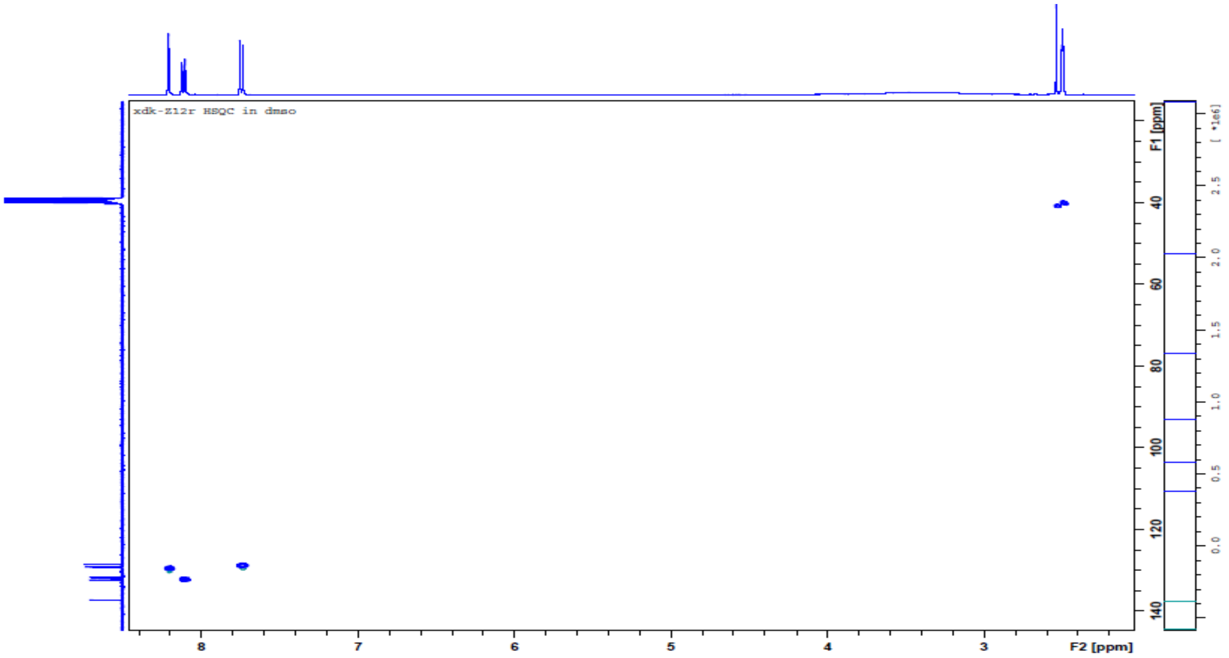

Appendix 76: HSQC spectrum of Z_{12}

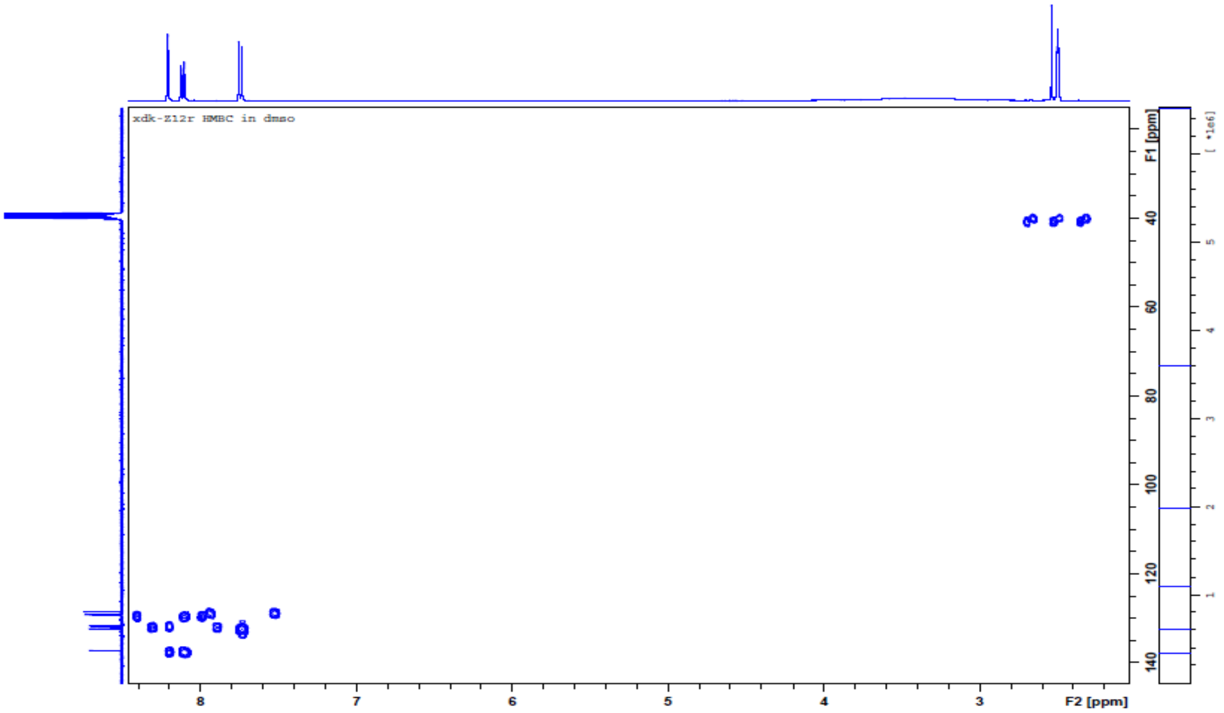

Appendix 77: HMBC spectrum of Z_{12}

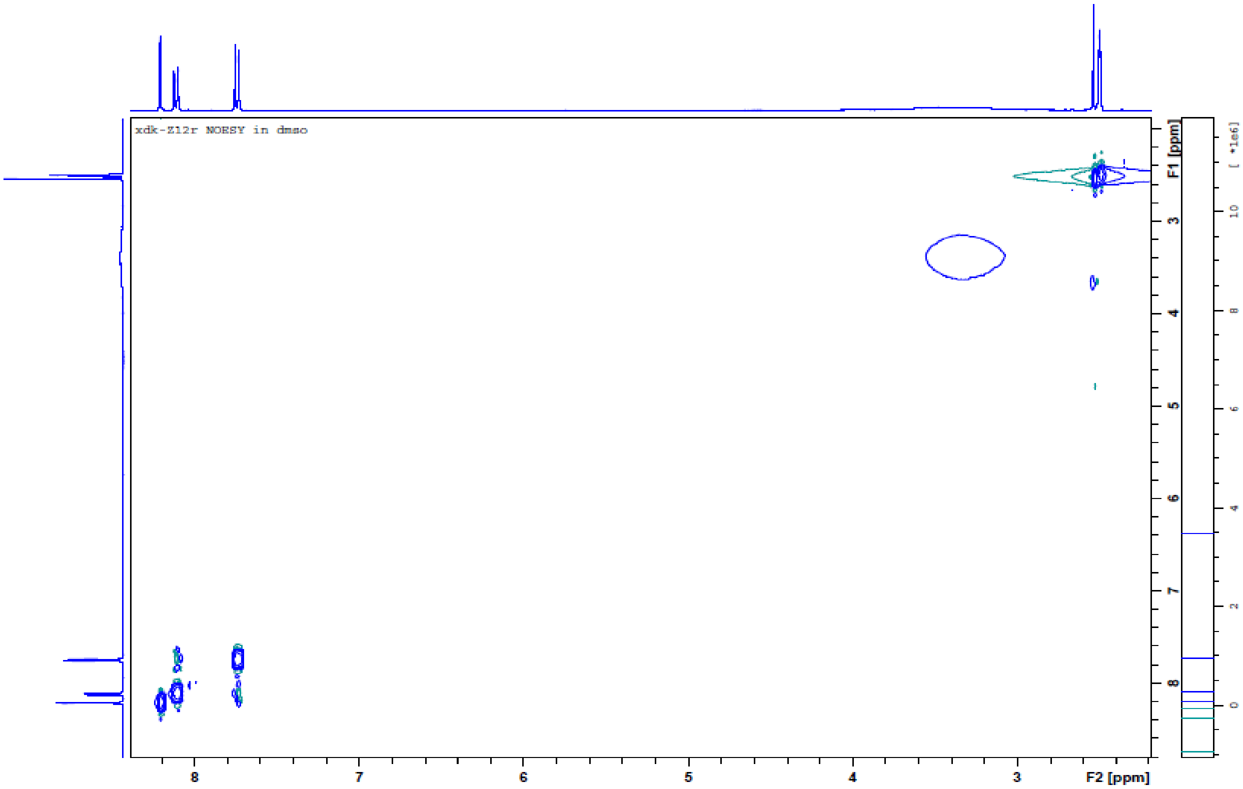

Appendix 78: NOESY spectrum of Z_{12}
